# 3,8-Disubstituted Pyrazolo[1,5-a]quinazoline as GABA_A_ Receptor Modulators: Synthesis, Electrophysiological Assays, and Molecular Modelling Studies

**DOI:** 10.3390/ijms251910840

**Published:** 2024-10-09

**Authors:** Letizia Crocetti, Gabriella Guerrini, Fabrizio Melani, Maria Paola Mascia, Maria Paola Giovannoni

**Affiliations:** 1 Neurofarba, Pharmaceutical and Nutraceutical Section, University of Florence, Via Ugo Schiff 6, 50019 Sesto Fiorentino, Italy; letizia.crocetti@unifi.it (L.C.); fabrizio.melani@unifi.it (F.M.); mariapaola.giovannoni@unifi.it (M.P.G.); 2 CNR-Institute of Neuroscience, Cagliari, Cittadella Universitaria, 09042 Monserrato, Italy; mariapaola.mascia@cnr.it

**Keywords:** GABA_A_ receptor modulators, pyrazolo[1,5-a]quinazoline, molecular docking, molecular dynamics, hierarchical cluster analysis (HCA), electrophysiological studies

## Abstract

As a continuation of our study in the field of GABA_A_ receptor modulators, we report the design and synthesis of new pyrazolo[1,5-a]quinazoline (PQ) bearing at the 8-position an oxygen or nitrogen function. All the final compounds and some intermediates, showing the three different forms of the pyrazolo[1,5-a]quinazoline scaffold (5-oxo-4,5-dihydro, -4,5-dihydro, and heteroaromatic form), have been screened with an electrophysiological technique on recombinant GABA_A_R (α1β2γ2-GABA_A_R), expressed in *Xenopus laevis* oocytes, by evaluating the variation in produced chlorine current, and permitting us to identify some interesting compounds (**6d**, **8a**, **8b**, and **14**) on which further functional assays were performed. Molecular modelling studies (docking, minimization of complex ligand–receptor, and MD model) and a statistical analysis by a Hierarchical Cluster Analysis (HCA) have collocated these ligands in the class corresponding to their pharmacological profile. The HCA results are coherent with the model we recently published (Proximity Frequencies), identifying the residues γThr142 and αHis102 as discriminant for the agonist and antagonist profile.

## 1. Introduction

The GABA_A_ receptor (GABA_A_R) belongs to the LGIC (Ligand-Gated Ion Channel) family arranged in a pentameric fashion. When the neurotransmitter GABA (γ-aminobutyric acid) interacts with the orthosteric binding sites in the receptor, the opening of the channel allows the influx of the chloride ions, driving the inhibitory neurotransmission and inducing postsynaptic hyperpolarization. Five subunits belonging to different families (α, β, γ, ρ, δ, ε, π, θ) concur to form the pentamer with various isoforms (α1-6, β1-3, γ1-3, ρ1-3, δ, ε, π, θ) and by convention, each subunit has a principal (+, plus) and a complementary side (−, minus). The most representative and prevalent pentamer contains two alpha, two beta, and one gamma subunit, and shows different and specific binding sites for GABA and benzodiazepines. The neurotransmitter GABA binds the two orthosteric binding sites to the interface between the α−/β+ subunits in the extracellular domain (ECD), thus suggesting that the two GABA-binding sites may not be functionally equivalent, with one having a threefold higher affinity for GABA than the other [1].

The ‘classic benzodiazepines’ (such as diazepam) bind the canonical site at the interface of the α+/γ− subunits in the ECD, even if three low-affinity binding sites located in the β/α and γ/β interface transmembrane domain (TMD) [2,3] and one between the α+/β− interface (the so-called low-affinity diazepam binding site) [2,4] and defined as the “pyrazoloquinolinone binding site” [5] have been evidenced and confirmed by the cryogenic electron microscopy (cryo-EM). The most significant GABA_A_ receptors present in the SNC and binding the ‘classic benzodiazepines’ are α1β2γ2-GABA_A_Rs, the most numerous population of receptors, followed by α2β2γ2- and α3β2γ2- and α5β2γ2-GABA_A_Rs. The α4β2γ2- and α6β2γ2-GABA_A_R do not bind the classic benzodiazepines since these alpha isoforms show an arginine101 (R101) instead of a histidine101 (H101), a fundamental residue involved in the agonist (diazepam, alprazolam) or antagonist (flumazenil) binding. On the other hand, these last two types of receptors (α4β2γ2- and α6β2γ2-GABA_A_R) could be involved in the potential therapeutic action of ‘pyrazoloquinolinones’ on neuropsychiatric disorders, as reported since the first decade of the 2000s [6,7,8,9,10,11].

Continuing our research on pyrazoloquinazolines (PQs) as ‘benzodiazepine receptor ligands’ [12], we report here the synthesis of new pyrazolo[1,5-a]quinazoline derivatives bearing at the 8-position an oxygen or nitrogen function, never inserted before, as well as other different groups/atoms to evaluate the ability of these heteroatoms to interact, through their lone pair, with receptor protein. All the final compounds and some intermediates, showing the three different forms of the pyrazolo[1,5-a]quinazoline scaffold (5-oxo-4,5-dihydro, -4,5-dihydro, and heteroaromatic form), have been screened with an electrophysiological technique on recombinant GABA_A_R (α1β2γ2-GABA_A_R), expressed in *Xenopus laevis* oocytes, by evaluating the variation in produced chlorine current, and where possible, a pharmacological profile has been defined (agonist/antagonist); then, molecular modelling studies (docking, minimization of complex ligand–receptor, and MD model) and a statistical analysis have been performed to further validate the model we recently published [13] by comparing the biological results and the prediction data.

## 2. Results and Discussion

### 2.1. Chemistry

**Scheme 1:**
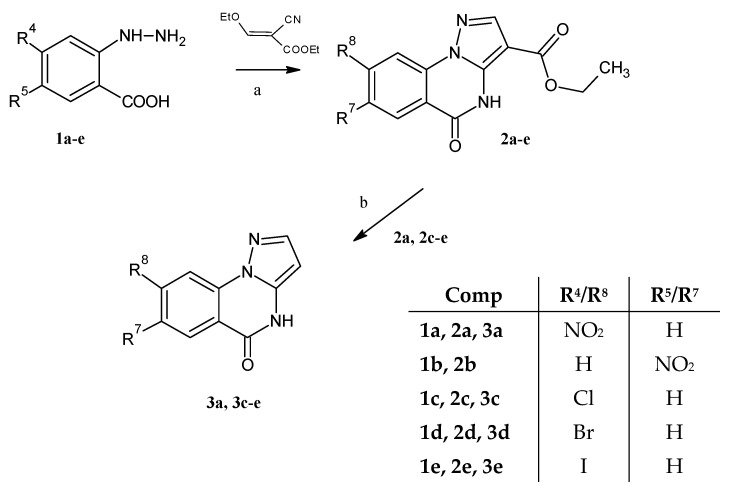
Reagents and conditions: (**a**) DMF, sodium acetate, reflux temperature, 2–5 h; (**b**) HCl for obtaining **3a**, reflux temperature; H_3_PO_4_ for obtaining **3c**–**e** at 150 °C.

**Scheme 2:**
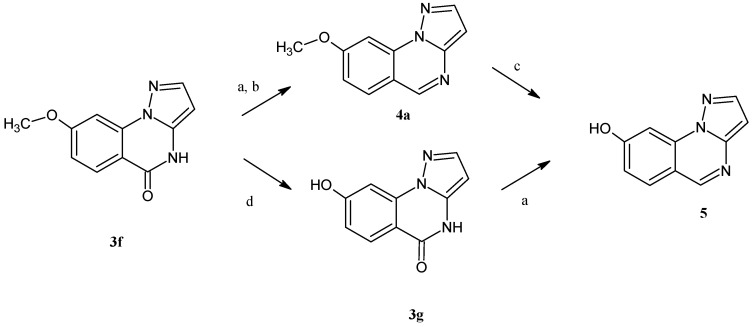
Reagents and conditions: (**a**) LiAlH_4_/THF abs., 50–60 °C; (**b**) toluene, Pd/C 10%, reflux temperature; (**c**) glacial AcOH/HBr 33% (1:1), reflux temperature; (**d**) H_3_PO_4_, 130 °C.

**Scheme 3:**
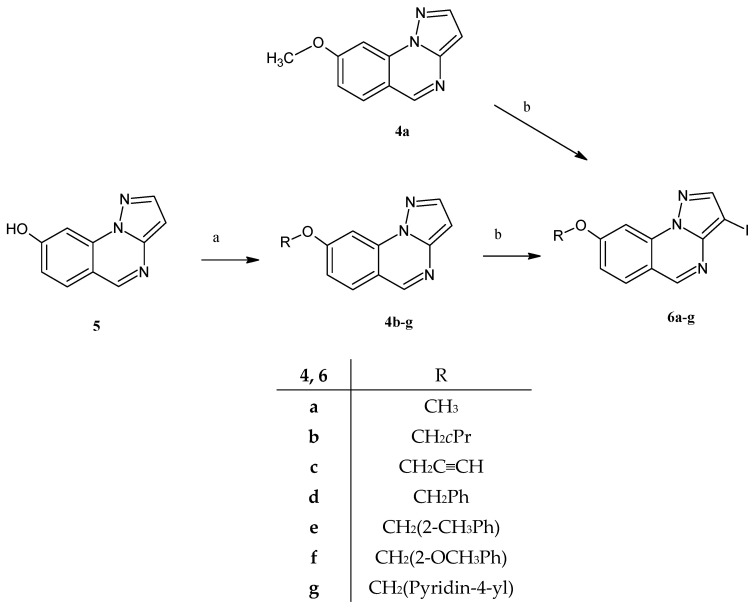
Reagents and conditions: (**a**) DMF abs., K_2_CO_3_, RX, 50–60 °C, 3–6 h; (**b**) DCM, NIS, 40 °C, 1 h.

**Scheme 4:**
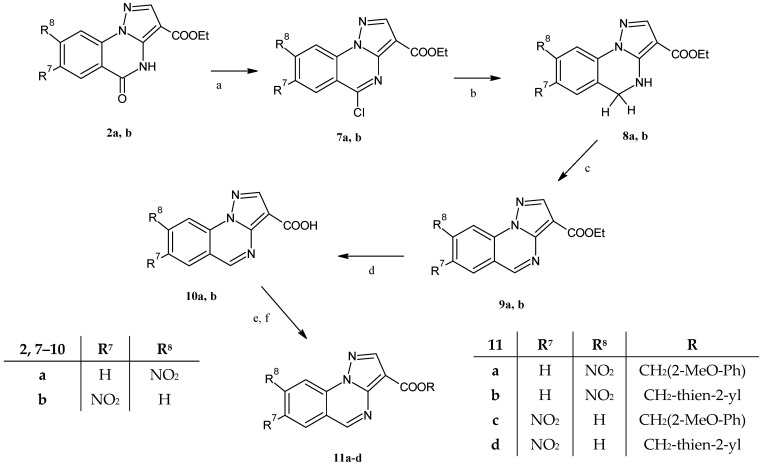
Reagents and conditions: (**a**) POCl_3_/PCl_5_, reflux temperature, 5 h; (**b**) EtOH/DCM (1:2), NaBH_4_, room temperature, 2 h; (**c**) toluene, Pd/C 10%, reflux temperature, 10 h; (**d**) HCl 6M/AcOH (1:3), 100 °C, 20 h; (**e**) SOCl_2_, reflux temperature, 1 h; (**f**) DCM, suitable alcohol, 50 °C; 2–5 h.

**Scheme 5:**
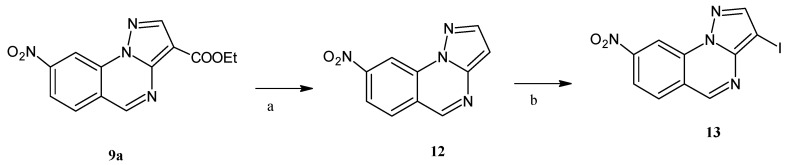
Reagents and conditions: (**a**) HCl, reflux temperature, 12 h; (**b**) DCM, NIS, 40–50 °C, 3 h.

**Scheme 6:**
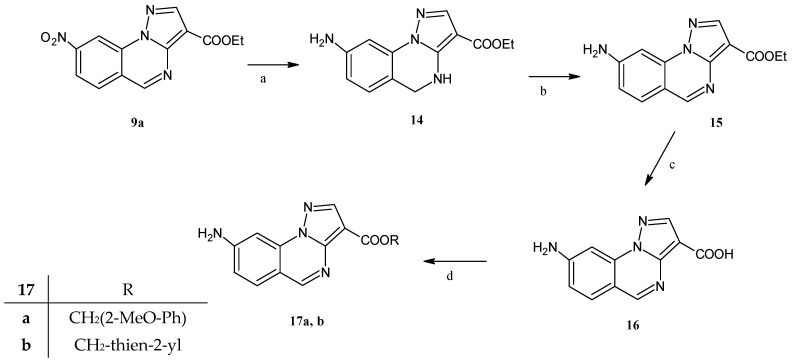
Reagents and conditions: (**a**) MeOH, HCOONH_4_, Pd/C 10%, room temperature, 4 h; (**b**) toluene, Pd/C 10%, reflux temperature; (**c**) NaOH 10% solution, 100 °C, and 8 h, then HCl; (**d**) (i) SOCl_2_, reflux temperature, 1 h, and (ii) DCM, suitable alcohol, 50 °C, 2–5 h.

**Scheme 7:**
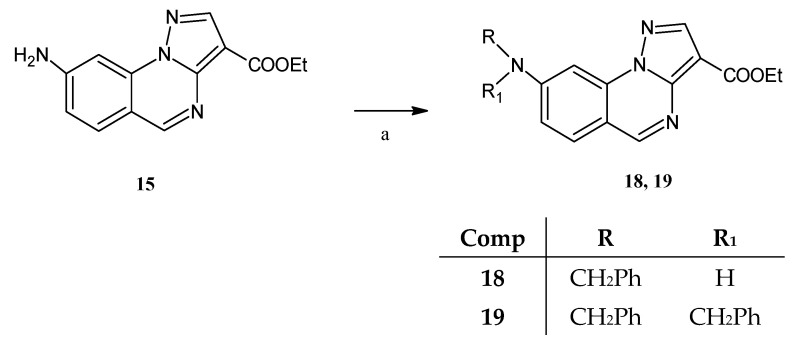
Reagents and conditions: (**a**) benzyl bromide, K_2_CO_3_ anhydrous, DMF anhydrous, 80 °C, 2 h.

The synthesis of the 5-oxo-4,5-dihydropyrazolo[1,5-a]quinazoline and of the corresponding 4,5-dehydro derivatives is depicted in Scheme 1, Scheme 2, Scheme 3, Scheme 4, Scheme 5, Scheme 6 and Scheme 7.

The cyclization reaction of the commercial 2-hydrazinyl benzoic acids (**1a**–**e**), variously substituted at position 4 or 5 (**1a** = 4-NO_2_, **1b** = 5-NO_2_, **1c** = 4-Cl, **1d** = 4-Br, **1e** = 4-I) with ethyl 2-cyano-3-ethoxyacrilate in DMF, gave the corresponding ethyl 5-oxo-4,5-dihydropyrazolo[1,5-a]quinazoline carboxylate substituted at the 8-position (**2a**, [14] **2c [12]**, **2d**–**e**), or 7-position (**2b**). The next decarboxylation at position 3 was made in conc. HCl for compound **2a** and H_3_PO_4_ for compounds **2c**–**e**, affording compounds **3a**, already obtained by Zhang with a different synthetic route [15], and **3c**–**e** (Scheme 1).

The previously synthesized 8-methoxypyrazolo[1,5-a]quinazolin-5(4H)-one **3f** [16] was chosen as the starting product for obtaining compound **5**, Scheme 2. Following the already reported method [16], **3f** was aromatized by treatment with LiAlH_4_ in anhydrous THF and then with Pd/C 10% to achieve **4a** [16], which, in turn, was subjected to demethylation with AcOH/HBr 33%, giving the final product **5** in low yield and in a very long time. Thus, to improve this reaction, we first transformed by demethylation with H_3_PO_4_ compound **3f** in 8-hydroxypyrazolo[1,5-a]quinazolin-5(4H)-one at **3g** and then, the reduction of the lactam function with LiAlH_4_ in anhydrous THF gave the desired compound **5** directly, without the need to use Pd/C in toluene.

Scheme 3 depicts the 8-O-alkylation of the 8-hydroxypyrazolo[1,5-a]quinazoline **5**, in the standard conditions (DMF/K_2_CO_3_/RX) to obtain compounds **4b**–**g**; the further iodination with NIS/DCM at position 3 of **4b**–**g** and **4a** [16] gave the final 3-iodopyrazolo[1,5-a]quinazoline 8-alkyl/8-alkyl(hetero)aryl **6a**–**g**.

The ethyl 8-nitro and the 7-nitro-5-oxo-4,5-dihydropyrazolo[1,5-a]quinazoline carboxylates **2a** and **2b**, respectively, were subjected to aromatization (Scheme 4) through a synthetic way already exploited in our laboratory. The transformation of **2a** and **2b** in the corresponding 5-chloro derivative with POCl_3_/PCl_5_ (**7a** and **7b [14]**) and the subsequent C-Cl bond reduction with NaBH_4_/DCM/EtOH gave the corresponding 4,5-dihydropyrazolo[1,5-a]quinazoline **8a** and **8b** in a good yield. The further treatment with Pd/C toluene at reflux permitted the aromatization of the pyrimidine ring of the scaffold, easily affording the ethyl 8/7-nitropyrazolo[1,5-a]quinazoline-3-carboxylate **9a** and **9b**. The next acid hydrolysis (HCl 6M/AcOH, 1:3) of the ethyl ester function of **9a,b** gave the corresponding 3-carboxylic acid **10a,b**, which underwent the standard esterification reaction (SOCl_2_, DCM/suitable alcohol) to obtain the final 3-(hetero)arylalkyloxy carbonyl derivatives (**11a**–**d**).

The ethyl 8-nitropyrazolo[1,5-a]quinazoline-3-carboxylate **9a** was decarboxylated in conc. HCl at reflux temperature, and the easily obtained 3-H derivate **12** was transformed into the corresponding 3-iodo derivative **13** using NIS/DCM, Scheme 5.

Finally, Scheme 6 depicts the synthetic route to obtain the esters **17a**,**b**, bearing the amino group at position 8. The starting product **9a** was treated with ammonium formate in methanol and Pd/C, affording the ethyl 8-amino-4,5-dihydropyrazolo [1,5-a]quinazoline-3-carboxylate **14**, which, by treatment with toluene/Pd/C at reflux, was converted into compound **15**; the latter was subjected to hydrolysis in an alkaline medium, and after the standard work-up, the 3-carboxylic acid **16** was recovered. Treatment with thionyl chloride and then with the suitable alcohol in DCM permitted us to obtain the 3-(2-methoxybenzyloxy)carbonyl- and the 3-(2-thienylmethoxy)carbonyl- derivatives **17a**,**b**. Compound **15** was also alkylated in standard conditions with benzyl bromide, obtaining N-benzyl (**18**) and N, N-dibenzyl derivatives (**19**), respectively, as shown in Scheme 7.

### 2.2. Biological Evaluation

As anticipated in the Introduction, the compounds were screened through electrophysiological techniques on recombinant α1β2γ2L-GABA_A_ receptors expressed in *Xenopus laevis* oocytes. The effects of compounds tested at 1–100 µM on the modulation of GABAA receptor function were assessed, and products for which no change in the chlorine current was recorded were also tested in the presence of the agonist lorazepam to determine whether they did not effectively bind the receptor or they exhibited an antagonist profile. Following this preliminary screening, we observed that all the 5-oxo-4,5-dihydro derivatives (compounds **2a**–**e**, **3a**, and **3c**–**g**), as well as the aromatic pyrazolo[1,5-a]quinazolines lacking the substituent at position 3 (**4a**–**g**, **5**, and **12**), do not bind the GABA_A_ receptor, suggesting that on the one hand the dihydro form is not appropriate for the target, and on the other hand that the substituent at position 3 is needed for the activity. Also, the 8-amino PQs **18** and **19**, N-benzyl- and N, N-dibenzyl derivatives, respectively, do not bind the GABA_A_ receptor, indicating that position 8 does not permit the presence of bulky substituents.

**Figure 1:**
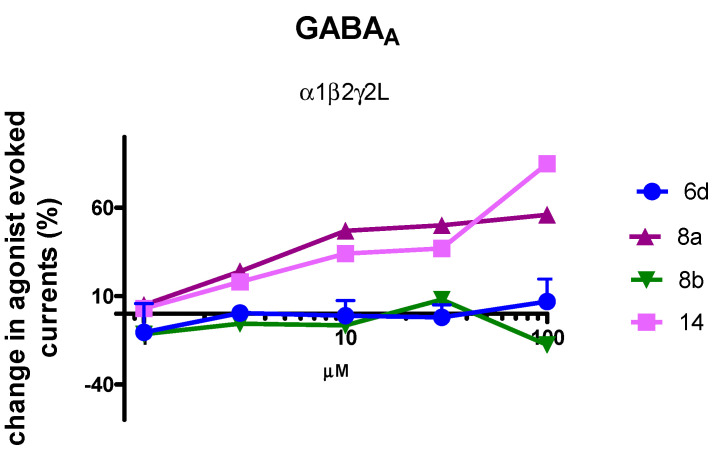
Concentration–response curves of compounds on GABA-induced Cl^−^ currents in *Xenopus laevis* oocytes expressing recombinant α1β2γ2L-GABA_A_ receptors. Data are expressed as the percentage modulation of the response induced by GABA at EC5-10 values and are the mean ± SEM of values obtained from two to nine oocytes.

**Figure 2:**
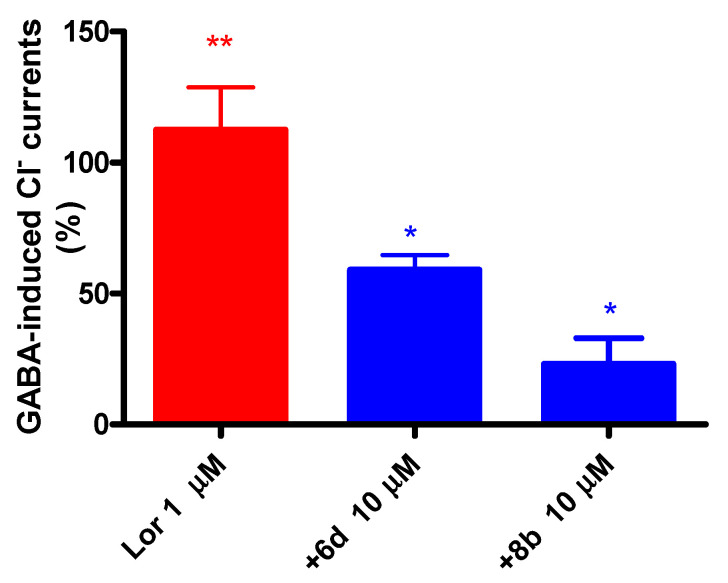
Compound **6d** and **8b** antagonized the potentiation of GABA-induced Cl^−^ currents by lorazepam in *Xenopus laevis* oocytes expressing recombinant α1β2γ2L-GABA_A_ receptors. Data are expressed as the percentage modulation of the response induced by GABA at EC5-10 values and are the mean ± SEM of values obtained from four oocytes. * *p* < 0.05. ** *p* < 0.01.

Instead, the remaining final compounds substituted at positions 3 and 7/8 show a certain ability to bind the GABA_A_ receptor. The most interesting electrophysiological results were obtained for compounds **6d**, **8a**, **8b**, and **14** (Figure 1). Among the 3-iodo derivatives, compound **6d**, which does not modulate the chlorine current, was evaluated for its ability to antagonize the full agonist lorazepam (1 µM) and its antagonist profile is evident in Figure 2. All other compounds bearing an alkoxy (**6a**–**c)** or a benzyloxy group (**6e**–**f**) and the 8-nitro **13** generally modulate the GABAA current in a discontinued manner, providing nonsignificant results (see Figure S1 panel **A**, Supporting Information, which depicts the profile of all compounds). Moving to the 3-ester derivatives bearing a nitro or an amino group at the 7/8-position of the pyrazolo[1,5-a]quinazoline scaffold (**8a**,**b**, **9a**,**b**, **11a**–**d**, **14**, **15**, **17a**,**b** (Figure S1 panel **B**, Supporting Information)), some interesting results emerge from electrophysiological assays for the two isomers’ ester derivatives **8a** and **8b** (8-nitro and 7-nitro derivatives, respectively). Compound **8a** enhances the chlorine current in the recombinant α1β2γ2L-GABA_A_R, reaching +57% at 100 µM; on the contrary, the 7-nitro isomer **8b** is an α1β2γ2L-GABA_A_R antagonist, Figure 1. Among the 8-amino derivatives (**14**, **15**, **17a,b**, Figure S1 panel **C**, Supporting Information), the ethyl 8-amino-4,5-dihydropyrazolo[1,5-a]quinazoline-3-carboxylate **14** emerges, enhancing the chlorine current until +85% at 100 µM (Figure 1). Finally, the aromatic analogue of **14** (compound **15**) as well as the 3-aryl(hetero)alkyl ester derivatives **17a,b** are able to modulate the chlorine current only weakly or not at all.

**Figure 3:**
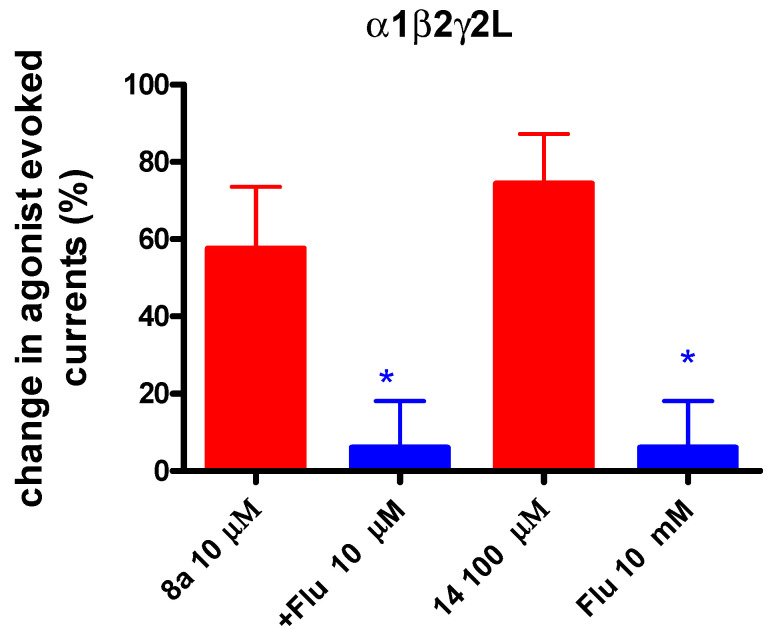
Compound **8a** and **14** antagonized by flumazenil in the potentiation of GABA-induced Cl^−^ currents by lorazepam in *Xenopus laevis* oocytes expressing recombinant α1β2γ2L-GABA_A_ receptors. Data are expressed as the percentage modulation of the response induced by GABA at EC5-10 values and are the mean ± SEM of values obtained from four oocytes. * *p* < 0.05.

The functional assay for **6d**, **8a**, **8b**, and **14** (Figure 2 and Figure 3) confirms the antagonist profile at the α1β2γ2L-GABA_A_R for **6d** and **8b**, since these two compounds reduce the potentiation of the lorazepam-induced chlorine current (Figure 2). The agonists’ profile at the α1β2γ2L-GABA_A_R of **8a** and **14** was then confirmed since their induced chlorine currents were reduced to 6% and to about 20%, respectively, by the antagonist flumazenil (Figure 3).

**Figure 4:**
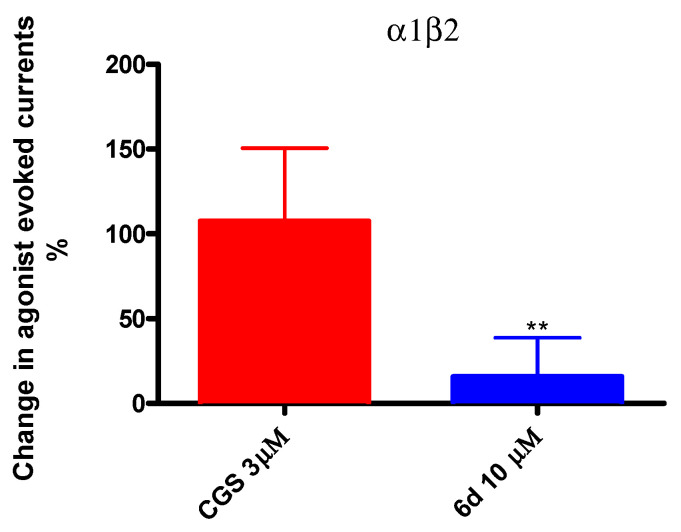
Compound **6d** reduced the potentiation of GABA-induced Cl^−^ currents by CGS 9895 in Xenopus oocytes expressing recombinant α1β2 GABAA receptors. Data are expressed as the percentage potentiation of the response induced by GABA at the EC5-10 value and are the mean + S.E.M of values obtained from 3 to 6 oocytes. ** *p* < 0.01.

As is well known, in addition to the high-affinity benzodiazepine binding site, the pentamer αβγ-GABA_A_R also shows a benzodiazepine low-affinity site, located in the extracellular domain at the α+/β− interface [2,4,17]. The pyrazoloquinolinone CGS 9895 is a null modulator (antagonist) at the high-affinity benzodiazepine binding site, but it is also able to act as a positive allosteric modulator [7]. According to several reports [8,11], it is considered that drugs acting at the “non-canonical” α+/β− low-affinity binding site might display potential clinical relevance; for example, they could be beneficial for long-term epilepsy treatment since they could interact with a broader variety of GABA_A_ receptor subtypes such as δ, ε, and π subunit-containing GABA_A_R. Therefore, we decided to further investigate compound **6d** in order to highlight a possible interaction with this ‘non-canonical’ Bz site. The data obtained with GABA_A_ receptors devoid of the γ subunit (α1β2) (Figure 4) indicate that, indeed, compound **6d** (10 µM) reduces in a statistically significant manner the potentiation of the GABA_A_ receptor induced by CGS 9895 (3 µM) (about −85%), suggesting that it binds the α+/β− low-affinity site too and might display potential clinical relevance.

### 2.3. Molecular Modelling Studies

To explain the different behaviour of the new ligands, the distances of the hydrogen bonds between the ligands and the amino acids of the active site were measured. The calculation was performed on 20 compounds, **6a**–**g**, **8a,b**, **9a,b**, **11a**–**d**, **14**, **15**, and **17a,b**.

The hydrogen bond lengths were measured on the structures, completely relaxed (i.e., at a minimum potential energy), of the ligand–receptor complex. The position of the ligand in the ligand–receptor complex was identified with the automatic docking programme AUTODOCK [18]. From AUTODOCK, it is possible to obtain more than one probable position (i.e., complex conformation) depending on the molecular flexibility. Thus, when this occurred, all the probable conformations were completely relaxed before measuring the hydrogen bond lengths. Lengths (H-acceptor atom) expressed in Angstroms (Å) measured in the various relaxed conformations of the ligand–receptor complexes are reported in Table 1 (as can be seen for compounds **6e**, **6f**, **9b**, **11b**, **11c**, **11d**, **17a**, and **17b**, the measurements were performed on multiple conformations).

The residues involved in hydrogen bond interaction with most compounds are αHis102, γThr142, and αSer205. The relatively “short” mean (m) hydrogen bond length for each residue, 2.19Å, 2.17Å, and 2.16Å, respectively, indicates strong bonds, while the relatively large standard deviation (sd) (0.28, 0.34, and 0.29, respectively) suggests a significant difference in bond strength between the ligands under consideration.

**Figure 5:**
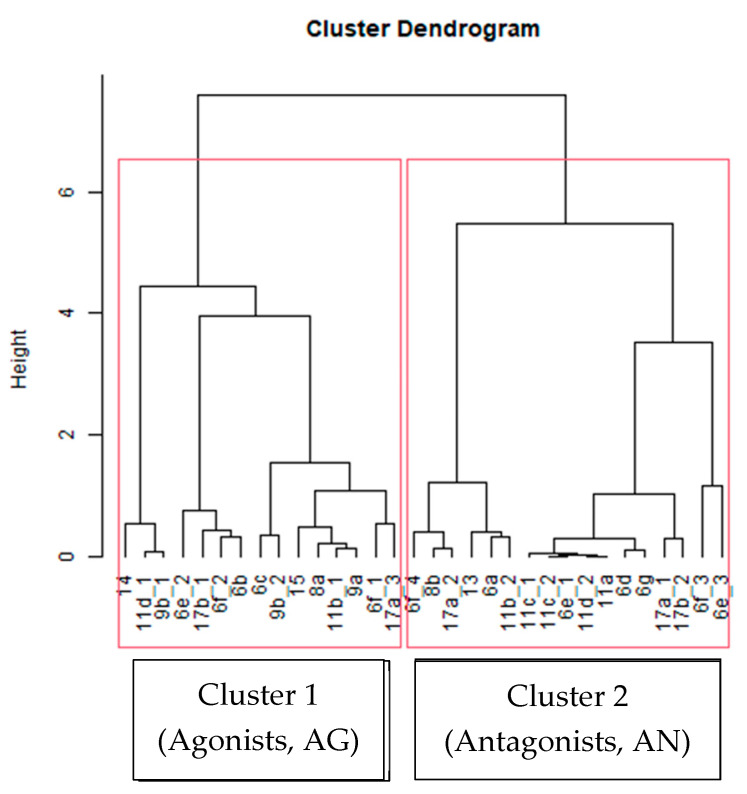
HCA for ligand (conformations)–amino acid (αHis102, γThr142, αSer205) hydrogen bond lengths (method of WARD.D2 and Euclidean distance).

Using the hydrogen bond lengths between ligands and the amino acids αHis102, γThr142, and αSer205 as variables for a Hierarchical Cluster Analysis (HCA) [19], it was possible to classify the ligands into two clusters (Figure 5).

HCA was performed using, as a similarity parameter, the Euclidean distance calculated in the three-dimensional space generated by the three variables (hydrogen bond lengths). WARD.D2 was chosen as the aggregation method.

The use of other or a greater number of variables (lengths) did not lead to an improvement in the classification; indeed, in some cases, classification errors occurred.

Biological results discussed above demonstrate that compounds **8a** and **14** exhibit an agonist profile, while compounds **8b** and **6d** act as antagonists. Supposing that a similar interacting force with amino acids in the binding site drove the binding of an agonist or antagonist, we could speculate that Cluster 1 (where **8a** and **14** are) gathered ligands whose conformations interact with the binding site as agonists and Cluster 2 (where **8b** and **6d** are) gathered ligands whose conformations bind the site as the antagonist. Thus, the HCA classification indicates a correspondence with a probable profile. Moreover, it is to be noted that for the ligands for which Autodock generates more conformations, the collocation could be non-univocal.

In Table 2, we report the HCA classification (agonist/antagonist (AG/AN), agreeing to Cluster 1 or 2) for the new 20 compounds and the correspondence with the biological results is reported in columns A and B. The value 1 in the A column indicates that the HCA collocation is in accordance with the biological test, while the same value in column B indicates that, at least, one conformation agrees with biological results; otherwise, the value 0 indicates no accordance. If columns A and B are empty, the biological results are unclear. The accuracy of the HCA ranges from about 60 to 75%, considering that not all poses of each compound are correctly located by the statistical method. From the HCA results, it could be speculated that the hydrogen bond lengths among αHis102, γThr142, αSer205, and ligands permit the discrimination of agonists from antagonists. Agonists show a stronger hydrogen bond interaction (shorter bond) with γThr142 than antagonists; the same thing happens with αHis102, while with αSer205, the bond length between agonists and antagonists is similar, and thus not relevant.

**Figure 6:**
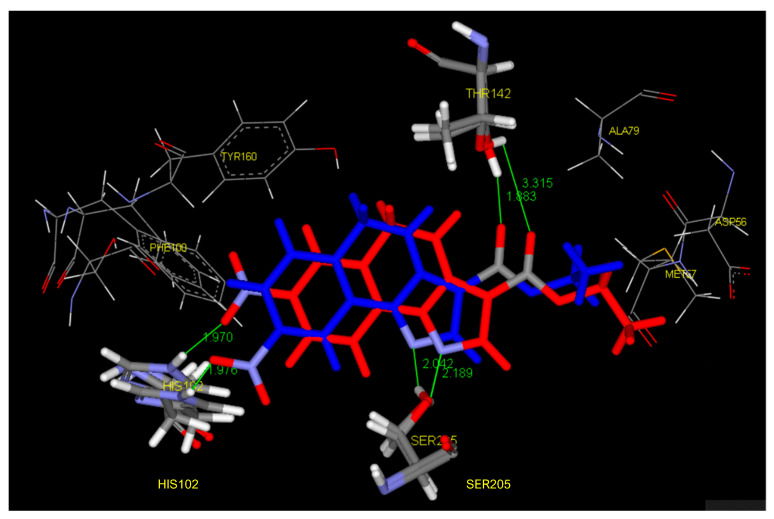
Hydrogen bonds among the agonist **8a** (blue), the antagonist **8b** (red), and γThr142, αHis102, and αSer205 in the binding site.

For the sake of clarity, the images of the minimized ligand–receptor complexes of the most interesting ligands (**6a**, **8a**, **8b**, and **14**) are reported below. The minimized ligand–receptor complexes of the two isomers **8a** and **8b** (8-nitro- and 7-nitro-4,5-dihydropyrazolo[1,5-a]quinazolin-3-carboxylate, respectively), to which the electrophysiological assays assigned an agonist (**8a**) and antagonist profile (**8b**), gave interesting information that allowed us to explain their different profiles. Figure 6 reports the complex ligand–receptor of the two isomers **8a** (blue) and **8b** (red), where the hydrogen bonds with γThr142, αHis102, and αSer205 are clearly evident.

In particular, compound **8a** (collocated in agonist class) engages with γThr142 and αHis102 strong hydrogen bonds (length 1.88 Å and 1.98 Å, respectively) through the oxygen carbonyl group of the ethyl ester moiety at the 3-position and the oxygen of the 8-nitro group. Compound **8b** (collocated in antagonist class) engages only a strong hydrogen bond with αHis102 (length 1.97 Å) through the 7-nitro group oxygen atoms; the carbonyl oxygen of the ester function does not engage the hydrogen bond with γThr142 (length 3.32 Å) probably because the whole molecule is shifted in the site since the driving force of the accommodation is the hydrogen bond engaged by the 7-nitro group with αHis102.

**Figure 7:**
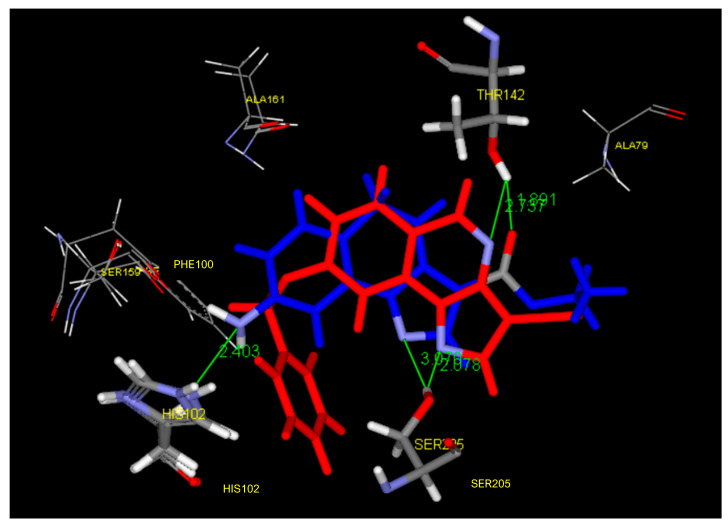
Hydrogen bonds among the agonist **14** (blue), the antagonist **6d** (red), and γThr142, αHis102, and αSer205 in the binding site.

Also, the agonist compound **14** (blue in Figure 7) in the minimized complex shows strong hydrogen bonds with γThr142 (length 1.89 Å) and weak hydrogen bonds (length 2.4 Å) with αHis102, while the antagonist **6d** (red in Figure 7) forms a very weak hydrogen bond with γThr142 (length 2.74 Å) and no hydrogen bond with αHis102. The Proximity Frequency (PF) values calculated by the molecular dynamics simulation (60 ns) [13] (see Supporting Information) for compounds **8a** and **8b** have highlighted that the agonist **8a** shows a Proximity Frequency with αVal203-γThr142 higher than the antagonist **8b.** These results are coherent with PFs’ model in which the agonist compounds were simultaneously close to the αVal203 and γThr142 amino acids (with a frequency of 37% compared to the frequency of 16% found by the antagonist compounds) while the antagonist compounds were simultaneously close to the αHis102, αTyr160, and γTyr58 amino acids (with a frequency of 35% against a frequency of 13% for agonist compounds).

## 3. Materials and Methods

### 3.1. Chemistry

All melting points were determined on a Büchi apparatus (New Castle, DE, USA) and are uncorrected. Extracts were dried over Na_2_SO_4_, and the solvents were removed under reduced pressure. Merck F-254 commercial plates (Merck, Durham, NC, USA) were used for analytical TLC to follow the course of reactions. Silica gel 60 (Merck 70-230 mesh, Merck, Durham, NC, USA) was used for column chromatography. ^1^H-NMR, and ^13^C-NMR, spectra were recorded on an Avance 400 instrument (Bruker Biospin Version 002 with SGU, Bruker Inc., Billerica, MA, USA). Chemical shifts (δ) are in parts per million (ppm) approximated by the nearest 0.01 ppm, using the solvent as an internal standard. Coupling constants *(J)* are in Hz; they were calculated by Top Spin 3.1 and approximated by 0.1 Hz. Data are reported as follows: the chemical shift, multiplicity (exch, exchange; br, broad; s, singlet; d, doublet; t, triplet; q, quartet; m, multiplet; or a combination of those, e.g., dd), integral, assignments, and coupling constant. All new compounds had a purity >95%; microanalyses indicated by symbols of the elements were performed on a Perkin-Elmer 260 elemental analyzer for C, H, and N, and they were within ±0.4% of the theoretical values.

**General procedure for the synthesis of compounds 2d**–**e:** The starting hydrazine (1d-e) (1.0 mmol) was reacted with ethyl 2-cyano-3-ethoxyacrylate (1.0 mmol) in abs. DMF (3 mL), and sodium acetate (1.1 mmol) was added. The reaction was maintained at reflux temperature until the starting material disappeared in TLC (eluent toluene/ethyl acetate/acetic acid, 8:2:1, *v*/*v*/*v*). After the addition of ice and water to the final solution, the formed precipitate was filtered and washed with water, ethanol, and diethyl ether. The recrystallization with ethanol gave a pure product.

**Ethyl 8-bromo-5-oxo-4,5-dihydropyrazole[1,5-a]quinazolin-3-carboxylate, 2d**

From hydrazine 1d; yield = 61%; mp = 227–228 °C. ^1^H-NMR (400 MHz, CDCl_3_) δ 1.39 (t, 3H, CH_2_*CH_3_*, *J* = 7.2 Hz), 4.38 (q, 2H, *CH*_2_CH_3_, *J* = 7.2 Hz), 7.62 (dd, 1H, H7, *J* = 8.4 Hz, *J* = 1.6 Hz), 8.05 (s, 1H, H2), 8.17 (d, 1H, H6, *J* = 8.4 Hz), 8.37 (d, 1H, H9, *J* = 1.2 Hz), 9.57 (bs, 1H, NH, exch.). IR cm^−^^1^ 3172, 1703, 1681. Anal. C_13_H_10_N_3_O_3_Br (C, H, N).

**Ethyl 8-iodo-5-oxo-4,5-dihydropyrazole[1,5-a]quinazolin-3-carboxylate, 2e**

From hydrazine 1e; yield = 58%; mp = 207–209 °C. ^1^H-NMR (400 MHz, DMSO-d_6_) δ 1.27 (t, 3H, CH_2_*CH*_3_, *J* = 6.8 Hz), 4.27 (q, 2H, *CH*_2_CH_3_, *J* = 6.8 Hz), 7.91–7.85 (m, 2H, H7, and H6), 8.16 (s, 1H, H2), 8.38 (s, 1H, H9), 11.65 (bs, 1H, NH, exch.). IR cm^−^^1^ 3154, 1702, 1680. Anal. C_13_H_10_N_3_O_3_I (C, H, N).

**General procedure for the synthesis of compounds 3a, and 3c**–**e:** To 0.6 mmol of the starting ethyl ester, 2a was decarboxylated by using conc. HCl (30 mL) at reflux temperature, while 2c-e were treated with polyphosphoric acid (2.6 mmol) and maintained at 150 °C. When the starting material disappeared, evaluating by TLC (eluent toluene/ethyl acetate/methanol, 8:2:1.5, *v*/*v*/*v*), the reaction (suspension or solution) was added to ice/water, and if a precipitate formed, this was worked up as usual; otherwise, the solution was extracted with ethyl acetate; the final solid was purified by recrystallization by a suitable solvent.

**8-Nitropyrazolo[1,5-a]quinazolin-5(4H)-one, 3a** [15]

By treatment of 2a with conc. HCl; from water. Yield = 65%; mp = >300 °C. ^1^H-NMR (400 MHz, DMSO-d_6_) δ 5.99 (d, 1H, H3, *J* = 2.0 Hz), 7.84 (d, 1H, H2, *J* = 1.6 Hz), 8.30 (m, 2H, H6 and H7), 8.61 (d, 1H, H9, *J* = 1.2 Hz), 11.93 (bs, 1H, NH, exch.). Anal. C_10_H_6_N_4_O_3_ (C, H, N).

**8-Chloroxypyrazolo[1,5-a]quinazolin-5(4H)-one, 3c**

By treatment of 2c with polyphosphoric acid; from ethanol. Yield = 70%; mp = 279–281 °C. ^1^H-NMR (400 MHz, DMSO-d_6_) δ 5.88 (d, 1H, H3, *J* = 1.6 Hz), 7.49 (dd, 1H H7, *J* = 8.8 Hz, *J* = 2 Hz), 7.78 (d, 1H, H2, *J* = 1.6 Hz), 8.00 (d, 1H, H9, *J* = 1.2 Hz), 8.09 (d, 1H, H6, *J* = 8.8), 12.25 (bs, 1H, NH, exch.). IR cm^−^^1^ 3134, 1681. Anal. C_10_H_6_N_3_OCl (C, H, N).

**8-Bromoxypyrazolo[1,5-a]quinazolin-5(4H)-one, 3d**

By treatment of 2d with polyphosphoric acid; from ethanol. Yield = 72%; mp = 268–270 °C. ^1^H-NMR (400 MHz, DMSO-d_6_) δ 5.90 (d, 1H, H3, *J* = 1.6 Hz), 7.64 (dd, 1H H7, *J* = 8.8 Hz, *J* = 2 Hz), 7.78 (d, 1H, H2, *J* = 1.6 Hz), 8.01 (d, 1H, H6, *J* = 8.8), 8.16 (d, 1H, H9, *J* = 1.2 Hz), 12.25 (bs, 1H, NH, exch.). Anal. C_10_H_6_N_3_OBr (C, H, N).

**8-Iodoxypyrazolo[1,5-a]quinazolin-5(4H)-one, 3e**

By treatment of 2e with polyphosphoric acid; from ethanol. Yield = 71%; mp = >300 °C. ^1^H-NMR (400 MHz, DMSO-d_6_) δ 5.88 (d, 1H, H3, *J* = 2.0 Hz), 7.78 (m, 3H; H2, H6, and H7), 8.36 (d, 1H, H9, *J* = 1.2 Hz), 12.21 (bs, 1H, NH, exch.). ESI-MS calcd. for C_10_H_6_N_3_OI, 311.07; found: m/z 328.10 [M + H]^+^. Anal. C_10_H_6_N_3_OI (C, H, N).

**8-Hydroxypyrazolo[1,5-a]quinazolin-5(4H)-one, 3g:** In a sealed tube, compound **4a** [16] (0.6 mmol) was treated with phosphoric acid at 120 °C; after monitoring by TLC (eluent toluene/ethyl acetate/methanol, 8:2:1.5, *v*/*v*/*v*), the final suspension was treated with water and the precipitate, filtered, was purified by ethanol. Yield = 65%; mp = >300 °C. ^1^H-NMR (400 MHz, DMSO-d_6_) δ 5.82 (d, 1H, H3, *J* = 1.6 Hz), 6.84 (dd, 1H H7, *J* = 8.4 Hz, *J* = 2 Hz), 7.35 (d, 1H, H9, *J* = 2.4 Hz), 7.72 (d, 1H, H2, *J* = 1.6 Hz), 7.93 (d, 1H, H6, *J* = 8.4), 10.88 (bs, 1H, OH, exch.), 11.90 (bs, 1H, NH, exch.). Anal. C_10_H_7_N_3_O_2_ (C, H, N).

**8-Hydroxypyrazolo[1,5-a]quinazoline, 5**

From **3g**, 8-hydroxypyrazolo[1,5-a]quinazolin-5(4H)-one, pathway *a* in Scheme 2: Compound **3g** (0.35 mmol) was suspended in THF abs (15 mL) heated at 50–60 °C and a LiAlH_4_ pellet (1:1.5) was added and maintained at refluxed temperature for 8 h. Monitoring the reaction by TLC (toluene/ethyl acetate/methanol, 8:2:1.5, *v*/*v*/*v*), it was possible to evidence the formation of the 4,5-dihydro derivative, which immediately converts into the 4,5-dehydro derivative. At the end of the reaction, after cooling to room temperature, the careful addition of ice/water caused gas evolution, and then the final solution was extracted with ethyl acetate. After the normal work-up, the residue was recovered by water. Yield 65%.

From **4a**, 8-methoxypyrazolo[1,5-a]quinazoline [16], pathway *c* in Scheme 2: Compound **4a** (0.35 mmol) was suspended in a mixture of AcOH/HBr 33% (20 mL, 1/1) and was refluxed for 72 h, monitoring the reaction by TLC (toluene/ethyl acetate/methanol, 8:2:1.5, *v*/*v*/*v*). The final solution was added to water, neutralized, extracted with ethyl acetate, and worked up as usual. The residue was recovered with water; yield 35%.

The final product **5** was purified by a chromatography column (TLC eluent), mp = 268–270 °C. ^1^H-NMR (400 MHz, DMSO-d_6_) δ 6.70 (d, 1H, H3, *J* = 1.6 Hz), 7.03 (dd, 1H H7, *J* = 8.4 Hz, *J* = 2 Hz), 7.60 (d, 1H, H9, *J* = 2.4 Hz), 7.99 (d, 1H, H6, *J* = 8.4), 8.09 (d, 1H, H2, *J* = 1.6 Hz), 8.83 (s, 1H, H5), 11.14 (bs, 1H, OH, exch.). Anal. C_10_H_7_N_3_O (C, H, N).

**General procedure for the synthesis of compounds 4b**–**g:** To 0.6 mmol of **5** in DMF abs (1.5 mL), we added anhydrous K_2_CO_3_ (1.2 mmol) and stirred for ten minutes, and then added the suitable alkylant (0.66 mmol, 1:1.1), maintaining the reaction at 80 °C. When the starting material disappeared (3–6 h), evaluated by TLC (eluent toluene/ethyl acetate/methanol, 8:2:1.5, *v*/*v*/*v*), ice/water were added and the precipitate was filtered, washed with water, and purified by recrystallization.

**8-(*c*-Propylmethoxy)pyrazolo[1,5-a]quinazoline, 4b**

Using *c*-propylbromide; yield 55%; mp = 103–105 °C, ethanol. ^1^H-NMR (400 MHz, CDCl_3_) δ 0.40 (m, 2H, CH_2_ *c*-prop); 0.70 (m, 2H, CH_2_ *c*-prop); 1.35 (m, 1H, CH_2_ *c*-prop), 4.04 (d, 2H CH_2_, *J* = 7.2 Hz), 6.73 (d, 1H, H3, *J* = 1.6 Hz), 7.12 (dd, 1H H7, *J* = 8.4 Hz, *J* = 2.0 Hz), 7.77 (d, 1H, H9, *J* = 2.4 Hz), 7.82 (d, 1H, H6, *J* = 8.4), 8.07 (d, 1H, H2, *J* = 1.6 Hz), 8.76 (s, 1H, H5). Anal. C_14_H_13_N_3_O (C, H, N).

**8-(2-Propynyloxy)pyrazolo[1,5-a]quinazoline, 4c**

Using propargyl bromide; yield 71%; mp = 151–153 °C, ethanol. ^1^H-NMR (400 MHz, CDCl_3_) δ 2.61 (t, 1H, HC≡, *J* = 2.4 Hz), 4.93 (d, 2H CH_2_, *J* = 2.4 Hz), 6.78 (d, 1H, H3, *J* = 1.6 Hz), 7.15 (dd, 1H H7, *J* = 8.4 Hz, *J* = 2.0 Hz), 7.87 (d, 1H, H6, *J* = 8.4), 7.95 (d, 1H, H9, *J* = 2.4 Hz), 8.10 (d, 1H, H2, *J* = 1.6 Hz), 8.79 (s, 1H, H5). Anal. C_13_H_9_N_3_O (C, H, N).

**8-(Benzyloxy)pyrazolo[1,5-a]quinazoline, 4d**

Using benzyl bromide; yield 66%; mp = 145–149 °C, ethanol. ^1^H-NMR (400 MHz, CDCl_3_) δ 5.32 (s, 2H CH_2_), 6.86 (d, 1H, H3, *J* = 2.0 Hz), 7.21 (dd, 1H H7, *J* = 8.4 Hz, *J* = 2.0 Hz), 7.38 (m, 1H, Ph), 7.43 (m, 2H, Ph), 7.49 (d, 2H, Ph, *J* = 7.2 Hz), 7.90 (d, 1H, H6, *J* = 8.4), 7.99 (d, 1H, H9, *J* = 2.4 Hz), 8.13 (d, 1H, H2, *J* = 1.6 Hz), 8.85 (s, 1H, H5). Anal. C_17_H_13_N_3_O (C, H, N).

**8-(2-Methylbenzyloxy)pyrazolo[1,5-a]quinazoline, 4e**

Using 2-methylbenzyl chloride; yield 66%; mp = 145–149 °C, *i*-propanol. ^1^H-NMR (400 MHz, CDCl_3_) δ 2.41 (s, 3H, CH_3_), 5.27 (s, 2H CH_2_), 6.78 (d, 1H, H3, *J* = 2.0 Hz), 7.16 (dd, 1H H7, *J* = 8.4 Hz, *J* = 2.0 Hz), 7.29 (m, 3H, Ph), 7.45 (d, 2H, Ph, *J* = 7.2 Hz), 7.85 (d, 1H, H6, *J* = 8.4), 7.99 (d, 1H, H9, *J* = 2.4 Hz), 8.10 (d, 1H, H2, *J* = 1.6 Hz), 8.79 (s, 1H, H5). Anal. C_18_H_15_N_3_O (C, H, N).

**8-(2-Methoxybenzyloxy)pyrazolo[1,5-a]quinazoline, 4f**

Using 2-methoxybenzyl chloride; yield 73%; mp = 116–118 °C, *i*-propanol. ^1^H-NMR (400 MHz, CDCl_3_) δ 3.85 (s, 3H, OCH_3_), 5.33 (s, 2H CH_2_), 6.75 (d, 1H, H3, *J* = 2.0 Hz), 6.87 (d, 1H, Ph, *J* = 8.8 Hz), 6.92 (m, 2H, Ph), 7.17 (dd, 1H H7, *J* = 8.4 Hz, *J* = 2.0 Hz), 7.48 (d, 2H, Ph, *J* = 7.2 Hz), 7.83 (d, 1H, H6, *J* = 8.4), 7.99 (d, 1H, H9, *J* = 2.4 Hz), 8.08 (d, 1H, H2, *J* = 1.6 Hz), 8.76 (s, 1H, H5). Anal. C_18_H_15_N_3_O_2_ (C, H, N).

**8-(Pyridin-4-ylmethoxy)pyrazolo[1,5-a]quinazoline, 4g**

Using 4-bromomethylpyridine hydrobromide; yield 80%; mp = 158–162 °C, *i*-propanol. ^1^H-NMR (400 MHz, CDCl_3_) δ 5.28 (s, 2H CH_2_), 6.77 (d, 1H, H3, *J* = 2.0 Hz), 7.20 (dd, 1H H7, *J* = 8.4 Hz, *J* = 2.0 Hz), 7.50 (d, 2H, Py, *J* = 5.6 Hz), 7.89 (d, 1H, H9, *J* = 1.6 Hz), 7.91 (d, 1H, H6, *J* = 8.8), 8.08 (d, 1H, H2, *J* = 1.6 Hz), 8.67 (d, 2H, Py, *J* = 6.0 Hz), 8.79 (s, 1H, H5). Anal. C_16_H_12_N_4_O (C, H, N).

**General procedure for the synthesis of compounds 6a**–**g:** To 0.5 mmol of starting material (**4a–g**) in DCM (5 mL), NIS (1.3 mmol) was added and stirred at 80 °C. When the starting material disappeared (1 h), evaluated by TLC (eluent toluene/ethyl acetate/methanol, 8:2:1.5, *v*/*v*/*v*), the solution was diluted with DCM (10 mL), and the organic layer was washed with a 5% NaOH solution; then, after the usual work-up, the residue was recovered by an ethanol/water solution and purified by recrystallization.

**3-Iodo-8-methoxypyrazolo[1,5-a]quinazoline, 6a** [16]

**3-Iodo-8-(*c*-propylmethoxy)pyrazolo[1,5-a]quinazoline, 6b**

Yield 55%; mp = 165–167 °C, ethanol. ^1^H-NMR (400 MHz, CDCl_3_) δ 0.41 (m, 2H, CH_2_ *c*-prop); 0.70 (m, 2H, CH_2_ *c*-prop); 1.35 (m, 1H, CH_2_ *c*-prop), 4.04 (d, 2H CH_2_, *J* = 7.2 Hz), 7.14 (dd, 1H H7, *J* = 8.4 Hz, *J* = 2.0 Hz), 7.74 (d, 1H, H9, *J* = 2.4 Hz), 7.85 (d, 1H, H6, *J* = 8.4), 8.09 (s, 1H, H2), 8.82 (s, 1H, H5). Anal. C_14_H_12_N_3_OI (C, H, N).

**3-Iodo-8-(2-propynyloxy)pyrazolo[1,5-a]quinazoline, 6c**

Yield 78%; mp = 179–181 °C, ethanol. ^1^H-NMR (400 MHz, CDCl_3_) δ 2.61 (t, 1H, HC≡, *J* = 2.4 Hz), 4.92 (d, 2H CH_2_, *J* = 2.4 Hz), 7.18 (dd, 1H H7, *J* = 8.4 Hz, *J* = 2.0 Hz), 7.90 (d, 1H, H6, *J* = 8.4), 7.92 (d, 1H, H9, *J* = 2.4 Hz), 8.18 (s, 1H, H2), 8.84 (s, 1H, H5). ^13^C-NMR (400 MHz, CDCl_3_) δ 29.70, 56.50, 97.28, 99.37, 116.12, 130.28, 142.97, 150.87. Anal. C_13_H_8_N_3_OI (C, H, N).

**3-Iodo-8-(benzyloxy)pyrazolo[1,5-a]quinazoline, 6d**

Yield 70%; mp = 176–178 °C, ethanol. ^1^H-NMR (400 MHz, CDCl_3_) δ 5.29 (s, 2H CH_2_), 7.20 (dd, 1H H7, *J* = 8.4 Hz, *J* = 2.0 Hz), 7.39 (m, 1H, Ph), 7.43 (m, 2H, Ph), 7.48 (d, 2H, Ph, *J* = 7.2 Hz), 7.87 (d, 1H, H6, *J* = 8.4), 7.92 (d, 1H, H9, *J* = 2.4 Hz), 8.10 (s, 1H, H2), 8.84 (s, 1H, H5). ^13^C-NMR (400 MHz, CDCl_3_) δ 70.93, 87.05, 96.62, 113.22, 116.94, 127.75, 128.57, 128.81, 130.43, 135.36, 138.18, 142.81, 151.75, 163.95. Anal. C_17_H_12_N_3_OI (C, H, N).

**3-Iodo-8-(2-methylbenzyloxy)pyrazolo[1,5-a]quinazoline, 6e**

Yield 70%; mp = 209–211 °C, *i*-propanol. ^1^H-NMR (400 MHz, CDCl_3_) δ 2.41 (s, 3H, CH_3_), 5.26 (s, 2H CH_2_), 7.18 (dd, 1H H7, *J* = 8.4 Hz, *J* = 2.0 Hz), 7.26 (m, 3H, Ph), 7.44 (d, 2H, Ph, *J* = 7.2 Hz), 7.88 (d, 1H, H6, *J* = 8.4), 7.94 (d, 1H, H9, *J* = 2.4 Hz), 8.12 (s, 1H, H2), 8.84 (s, 1H, H5). ^13^C-NMR (400 MHz, CDCl_3_) δ 18.99, 52.59, 69.58, 96.44, 113.30, 116.98, 126.24, 128.95, 129.04, 130.46, 130.66, 133.29, 137.04, 147.06, 152.13, 164.10. Anal. C_18_H_14_N_3_OI (C, H, N).

**3-Iodo-8-(2-methoxybenzyloxy)pyrazolo[1,5-a]quinazoline, 6f**

Yield 90%; mp = 154–156 °C, *i*-propanol. ^1^H-NMR (400 MHz, CDCl_3_) δ 3.90 (s, 3H, OCH_3_), 5.33 (s, 2H CH_2_), 6.94 (d, 1H, Ph, *J* = 8.8 Hz), 6.99 (t, 1H, Ph, *J* = 7.6 Hz), 7.20 (dd, 1H H7, *J* = 8.4 Hz, *J* = 2.0 Hz), 7.33 (t, 1H, Ph, *J* = 7.2 Hz), 7.48 (d, 1H, Ph, *J* = 7.6 Hz), 7.86 (d, 1H, H6, *J* = 8.4), 7.97 (d, 1H, H9, *J* = 2.4 Hz), 8.10 (s, 1H, H2), 8.83 (s, 1H, H5). Anal. C_18_H_14_N_3_O_2_I (C, H, N).

**3-Iodo-8-(pyridin-4-ylmethoxy)pyrazolo[1,5-a]quinazoline, 6g**

Yield 68%; mp = 190–192 °C, *i*-propanol. ^1^H-NMR (400 MHz, CDCl_3_) δ 5.34 (s, 2H CH_2_), 7.22 (dd, 1H H7, *J* = 8.4 Hz, *J* = 2.0 Hz), 7.48 (d, 2H, Py, *J* = 5.6 Hz), 7.90 (d, 1H, H9, *J* = 1.6 Hz), 7.92 (d, 1H, H6, *J* = 8.8), 8.10 (s, 1H, H2), 8.68 (d, 2H, Py *J* = 6.0 Hz), 8.85 (s, 1H, H5). Anal. C_16_H_11_N_4_OI (C, H, N).

**General procedure for the synthesis of compounds 7a,b:** To 0.5 mmol of starting material (**4**, **5**), POCl_3_ (3 mL) and PCl_5_ (0.5 mmol) were added and stirred at reflux temperature for 5 h. When the starting material disappeared, evaluated by TLC (eluent toluene/ethyl acetate/methanol, 8:2:1.5, *v*/*v*/*v*), the solution was evaporated under vacuum and the addition of ice/water gave a precipitate, which was filtered and used as such for the next step.

**Ethyl 8-nitro-5-chloropyrazolo[1,5-a]quinazoline-3-carboxylate, 7a**

Yield 85%; mp = 199–202 °C. ^1^H-NMR (400 MHz, DMSO-d_6_) δ 1.32 (t, 3H CH_3_, *J* = 6.8 Hz), 4.33 (q, 2H, CH_2_, *J* = 7.2 Hz), 8.47 (dd, 1H H7, *J* = 7.2 Hz, *J* = 2.0 Hz), 8.58 (d, 1H, H6, *J* = 8.8), 8.69 (s, 1H, H2), 9.01 (d, 1H, H9, *J* = 2.0 Hz). ^13^C-NMR (400 MHz, DMSO-d_6_) δ 14.77, 60.69, 106.16, 117.60, 117.93, 124.13, 130.85, 139.53, 143.85, 145.47, 146.86, 154.07, 161.43. Anal. C_13_H_9_N_4_O_4_Cl (C, H, N).

**Ethyl 7-nitro-5-chloropyrazolo[1,5-a]quinazoline-3-carboxylate, 7b**

Yield 85%; mp = 199–202 °C. ^1^H-NMR (400 MHz, DMSO-d_6_) δ 1.33 (t, 3H CH_3_, *J* = 6.8 Hz), 4.33 (q, 2H, CH_2_, *J* = 7.2 Hz), 8.62 (d, 1H, H9, *J* = 8.4), 8.69 (s, 1H, H2), 8.85 (dd, 1H H8, *J* = 8.0 Hz, *J* = 2.0 Hz), 8.96 (d, 1H, H6, *J* = 2.0 Hz). ^13^C-NMR (400 MHz, DMSO-d_6_) δ 14.77, 60.69, 106.16, 117.60, 117.93, 124.13, 130.85, 139.53, 143.85, 145.47, 146.86, 154.07, 161.43. Anal. C_13_H_9_N_4_O_4_Cl (C, H, N).

**General procedure for the synthesis of compounds 8a,b:** A solution of 0.6 mmol of starting material (**7a,b**) in ethanol/DCM (15 mL/25 mL) was added to sodium borohydride in a large excess (6.0 mmol) and the reaction was maintained at room temperature under stirring for 2 h. The final suspension was evaporated to dryness and the residue was recovered with water, filtered, and washed with water.

**Ethyl 8-nitro-4,5-dihydropyrazolo[1,5-a]quinazoline-3-carboxylate, 8a**

Yield 83%; mp = 222–224 °C, ethanol. ^1^H-NMR (400 MHz, DMSO-d_6_) δ 1.24 (t, 3H CH_3_, *J* = 6.8 Hz), 4.18 (q, 2H, CH_2_, *J* = 7.2 Hz), 4.65 (s, 2H, CH_2_), 7.31 (s, 1H NH, exch.), 7.58 (d, 1H, H6, *J* = 8.8), 7.76 (s, 1H, H2), 8.00 (dd, 1H H7, *J* = 7.2 Hz, *J* = 2.0 Hz), 8.14 (d, 1H, H9, *J* = 2.0 Hz). ^13^C-NMR (400 MHz, DMSO-d_6_) δ 14.93, 42.74, 59.62, 94.74, 108.37, 120.59, 128.20, 128.80, 134.92, 143.13, 148.73, 148.75, 162.78. IR, cm^−^^1^ (nujiol) 3373, 1674, 1527, 1350. Anal. C_13_H_12_N_4_O_4_ (C, H, N).

**Ethyl 7-nitro-4,5-dihydropyrazolo[1,5-a]quinazoline-3-carboxylate, 8b**

Yield 78%; mp = 239–241 °C, *i*-propanol. ^1^H-NMR (400 MHz, DMSO-d_6_) δ 1.24 (t, 3H CH_3_, *J* = 6.8 Hz), 4.18 (q, 2H, CH_2_, *J* = 7.2 Hz), 4.65 (s, 2H, CH_2_), 7.39 (s, 1H NH, exch.), 7.69 (d, 1H, H9, *J* = 2.0 Hz), 7.79 (s, 1H, H2), 8.22 (m, 2H, H6, and H8). ^13^C-NMR (400 MHz, DMSO-d_6_) δ 14.93, 42.70, 59.63, 93.00, 114.69, 122.05, 123.24, 125.08, 139.08, 143.93, 144.77, 149.34. IR, cm^−^^1^ (nujiol) 3373, 1674, 1527, 1350. Anal. C_13_H_12_N_4_O_4_ (C, H, N).

**General procedure for the synthesis of compounds 9a,b:** To 0.5 mmol of starting material (**8a,b**) in toluene (50 mL), we added Pd/C 10% and the reaction was maintained at refluxed temperature for 10 h, monitoring the reaction by TLC (eluent toluene/ethyl acetate/methanol, 8:2:1.5, *v*/*v*/*v*). Then, the suspension was filtered and the solution was evaporated under vacuum; the precipitate was recovered with *i*-propanol.

**Ethyl 8-nitropyrazolo[1,5-a]quinazoline-3-carboxylate, 9a**

Yield 56%; mp = 228–230 °C, ethanol. ^1^H-NMR (400 MHz, DMSO-d_6_) δ 1.32 (t, 3H CH_3_, *J* = 6.8 Hz), 4.33 (q, 2H, CH_2_, *J* = 7.2 Hz), 8.47 (dd, 1H H7, *J* = 7.2 Hz, *J* = 2.0 Hz), 8.57 (d, 1H, H6, *J* = 8.8), 8.69 (s, 1H, H2), 9.03 (d, 1H, H9, *J* = 2.0 Hz), 9.52 (s, 1H, H5). ^13^C-NMR (400 MHz, DMSO-d_6_) δ 14.85, 60.50, 110.71, 121.04, 132.30, 146.46, 156.06. Anal. C_13_H_10_N_4_O_4_ (C, H, N).

**Ethyl 7-nitropyrazolo[1,5-a]quinazoline-3-carboxylate, 9b**

Yield 82%; mp = 239–241 °C, ethanol. ^1^H-NMR (400 MHz, DMSO-d_6_) δ 1.32 (t, 3H CH_3_, *J* = 6.8 Hz), 4.32 (q, 2H, CH_2_, *J* = 7.2 Hz), 8.57 (d, 1H, H9, *J* = 8.4), 8.68 (s, 1H, H2), 8.79 (dd, 1H H8, *J* = 8.0 Hz, *J* = 2.0 Hz), 9.28 (d, 1H, H6, *J* = 2.0 Hz), 9.54 (s, 1H, H5). ^13^C-NMR (400 MHz, DMSO-d_6_) δ 14.84, 60.51, 117.05, 126.38, 129.92. Anal. C_13_H_10_N_4_O_4_ (C, H, N).

**General procedure for the synthesis of compounds 10a,b:** To a mixture of HCl conc. and AcOH (1:3, 4 mL/12 mL), 0.5 mmol of starting material (**30**–**31**) was added and maintained at reflux temperature for 20 h. The final suspension of acid was filtered and used as such for the next step.

**8-Nitropyrazolo[1,5-a]quinazoline-3-carboxylic acid, 10a**

Yield 35%; mp > 300 °C. ^1^H-NMR (400 MHz, DMSO-d_6_) δ 8.45 (d, 1H, H6, *J* = 8.8), 8.56 (d, 1H H7, *J* = 7.2 Hz), 8.62 (s, 1H, H2), 8.99 (s, 1H, H9), 9.48 (s, 1H, H5), 12.69 (bs, 1H, OH, exch.). ^13^C-NMR (400 MHz, DMSO-d_6_) δ 14.85, 60.50, 110.71, 121.04, 132.30, 146.46, 156.06. Anal. C_11_H_6_N_4_O_4_ (C, H, N).

**7-Nitropyrazolo[1,5-a]quinazoline-3-carboxylic acid, 10b**

Yield 30%; mp = >300 °C. ^1^H-NMR (400 MHz, DMSO-d_6_) δ 8.58–8.78 (m, 3H, H2, H8, and H9), 9.26 (d, 1H, H6, *J* = 2.0 Hz), 9.52 (s, 1H, H5), 12.7 (bs, 1H OH, exch.). ^13^C-NMR (400 MHz, DMSO-d_6_) δ 14.84, 60.51, 117.05, 126.38, 129.92. Anal. C_11_H_6_N_4_O_4_ (C, H, N).

**General procedure for the synthesis of compounds 11a**–**d:** Starting carboxylic acid (**10a,b**, 0.5 mmol) was treated with SOCl_2_ and maintained at reflux temperature for 1 h; after the evaporation of the excess reagent, DCM (10 mL) and the suitable alcohol (0.7 mmol) were added. The reaction was monitored by TLC (eluent toluene/ethyl acetate/acetic acid, 8:2:1, *v*/*v*/*v*) and at the end, the solvent evaporated, and the residue was recovered by ethanol and recrystallized by ethanol.

**2-Methoxybenzyl 8-nitropyrazolo[1,5-a]quinazoline-3-carboxylate, 11a**

From **10a** and 2-methoxybenzyl alcohol; yield 25%; mp = 165–166 °C. ^1^H-NMR (400 MHz, DMSO-d_6_) δ 3.81 (s, 3H, OCH_3_), 5.35 (s, 2H, CH_2_), 8.45 (d, 1H, H6, *J* = 8.8), 8.56 (d, 1H H7, *J* = 7.2 Hz), 8.73 (s, 1H, H2), 8.02 (s, 1H, H9), 9.55 (s, 1H, H5). ^13^C-NMR (400 MHz, DMSO-d_6_) δ 14.85, 60.50, 110.71, 121.04, 132.30, 146.46, 156.06. Anal. C_19_H_14_N_4_O_5_ (C, H, N).

**Thien-2-ylmethyl 8-nitropyrazolo[1,5-a]quinazoline-3-carboxylate, 11b**

From **10a** and 2-thienylmethanol; yield 25%; mp = 176–178 °C. ^1^H-NMR (400 MHz, DMSO-d_6_) δ 5.54 (s, 2H, CH_2_), 7.03 (m, 1H, H4 thiophene), 7.26 (m, 1H, H3 thiophene), 7.54 (d, 1H, H5 thiophene, *J* = 4.4 Hz), 8.48 (d, 1H, H6, *J* = 8.8), 8.57 (d, 1H H7, *J* = 7.2 Hz), 8.70 (s, 1H, H2), 9.01 (s, 1H, H9), 9.53 (s, 1H, H5). ^13^C-NMR (400 MHz, DMSO-d_6_) δ 60.44, 110.67, 121.06, 122.17, 127.31, 128.04, 129.13, 132.38, 135.56, 138.68, 146.14, 156.02, 161.64. Anal. C_16_H_10_N_4_O_4_S (C, H, N).

**2-Methoxybenzyl 7-nitropyrazolo[1,5-a]quinazoline-3-carboxylate, 11c**

From **10b** and 2-methoxybenzyl alcohol; yield 30%; mp = 176–178 °C. ^1^H-NMR (400 MHz, DMSO-d_6_) δ 3.72 (s, 3H, OCH_3_), 4.51 (s, 2H, CH_2_), 6.89 (d, 1H, Ph, *J* = 6.0 Hz), 7.16 (m, 3H, Ph), 7.52 (d, 1H, H9, *J* = 8.4), 7.68 (s, 1H, H2), 8.25 (d, 1H, H8, *J* = 8.0 Hz), 8.43 (s, 1H, H6), 9.13 (s, 1H, H5). ^13^C-NMR (400 MHz, DMSO-d_6_) δ 14.85, 60.50, 110.71, 121.04, 132.30, 146.46, 156.06. Anal. C_19_H_14_N_4_O_5_ (C, H, N).

**Thien-2-ylmethyl 7-nitropyrazolo[1,5-a]quinazoline-3-carboxylate, 11d**

From **10b** and 2-thienylmethanol; yield 20%; mp = 201–204 °C. ^1^H-NMR (400 MHz, DMSO-d_6_) δ 4.64 (s, 2H, CH_2_), 6.92 (m, 1H, H4 thiophene), 6.98 (m, 1H, H3 thiophene), 7.48 (d, 1H, H5 thiophene, *J* = 4.4 Hz), 7.55 (d, 1H, H9, *J* = 8.4 Hz), 7.69 (s, 1H, H2), 8.19 (d, 1H H8, *J* = 8.8 Hz), 8.43 (d, 1H, H6, *J* = 1.2 Hz), 9.19 (s, 1H, H5). ^13^C-NMR (400 MHz, DMSO-d_6_) δ 60.44, 110.67, 121.06, 122.17, 127.31, 128.04, 129.13, 132.38, 135.56, 138.68, 146.14, 156.02, 161.64. ESI-MS calcd. for C_16_H_10_N_4_O_4_S, 354.04; found: m/z 354.04 [M + H]^+^. Anal. C_16_H_10_N_4_O_4_S (C, H, N).

**8-Nitropyrazolo[1,5-a]quinazoline, 12:** Ethyl ester **9a** (100 mg, 0.35 mmol) was suspended in HCl conc (20 mL) and maintained at reflux temperature until the starting material disappeared, evaluated by TLC (eluent toluene/ethyl acetate/methanol, 8:2:1.5, *v*/*v*/*v*). The acid phase was extracted with ethyl acetate and washed twice with water. The evaporation to dryness gave a white residue, which was recovered by water, filtered under suction, and recrystallized by ethanol. Yield 30%; mp = 160–162 °C. ^1^H-NMR (400 MHz, DMSO-d_6_) δ 7.00 (d, 1H, H3, *J* = 2.0 Hz), 8.34 (d, 1H, H2, *J* = 2.0 Hz), 8.53 (d, 1H, H6, *J* = 8.8 Hz), 8.74 (dd, 1H, H7, *J* = 8.2 Hz, *J* = 2.4 Hz), 9.21 (d, 1H, H9, *J* = 2.0 Hz), 9.28 (s, 1H, H5). ^13^C-NMR (400 MHz, DMSO-d_6_) δ 14.85, 60.50, 110.71, 121.04, 132.30, 146.46, 156.06. Anal. C_10_H_6_N_4_O_2_ (C, H, N).

**3-Iodo-8-nitropyrazolo[1,5-a]quinazoline, 13:** To a solution of compound **12** (100 mg, 0.47 mmol) in methylene chloride (10 mL), we added NIS (1:1.1) and maintained it at 40–50 °C until the starting material disappeared in TLC (eluent toluene/ethyl acetate, 8:2, *v*/*v*). The final yellow solution was washed with water made alkaline with NaOH and, after the usual work-up, evaporated to dryness. The residue was recovered by ethanol and recrystallized by the same solvent. Yield 30%; mp = 160–162 °C. ^1^H-NMR (400 MHz, CDCl_3_) δ 7.24 (s, 1H, H2), 8.19 (d, 1H, H6, *J* = 8.8 Hz), 8.36 (d, 1H, H7, *J* = 8.2 Hz), 9.00 (s, 1H, H9), 9.29 (s, 1H, H5). ^13^C-NMR (400 MHz, DMSO-d_6_) δ 109.58, 120.48, 122.15, 132.18, 135.86, 148.32, 150.65, 153.41. Anal. C_10_H_5_N_4_O_2_I (C, H, N).

**Ethyl 8-amino-4,5-dihydropyrazolo[1,5-a]quinazoline-3-carboxylate, 14:** A solution of the starting product (**9a**, 0.7 mmol, 200 mg) in methanol (20 mL), ammonium formate (0.21 mmol, 132 mg), and a catalytic amount of Pd/C 10% was maintained at room temperature, under stirring, for 4 h. The final suspension, monitored by TLC (eluent toluene/ethyl acetate/methanol, 8:2:1.5, *v*/*v*/*v*), was filtered and evaporated to dryness and the final residue was recovered with water, filtered, and separated by a chromatography column (eluent toluene/ethyl acetate/methanol, 8:2:1.5, *v*/*v*/*v*) as the faster band running. Yield 45%; mp = 145–148 °C. ^1^H-NMR (400 MHz, DMSO-d_6_) δ 1.23 (t, 3H CH_3_, *J* = 6.8 Hz), 4.16 (q, 2H, CH_2_, *J* = 7.2 Hz), 4.32 (s, 2H, CH_2_); 5.27 (bs, 2H, NH_2_, exch.); 6.33 (dd, 1H, H7, *J* = 8.0 Hz, *J* = 2.0 Hz), 6.91 (d, 1H, H9, *J* = 2.0 Hz), 6.87 (d, 1H, H6, *J* = 8.0 Hz), 6.92 (bs, 1H, NH, exch.), 7.61 (s, 1H, H2). ^13^C-NMR (400 MHz, DMSO-d_6_) δ 14.81, 60.16, 93.57, 103.34, 110.61, 115.91, 131.48, 138.12, 145.28, 145.59, 154.04, 156.04, 162.83. IR nujiol cm^−^^1^: 3360, 3196, 1713,1587, 1263, 1111. Anal. C_13_H_12_N_4_O_2_ (C, H, N).

**Ethyl 8-aminopyrazolo[1,5-a]quinazoline-3-carboxylate, 15:** Starting material **14** (1.0 mmol) was refluxed with toluene and Pd/C 10% for 5 h, and when the final product was completely formed (evaluated by TLC; eluent toluene/ethyl acetate/methanol, 8:2:1.5, *v*/*v*/*v*), the final suspension was filtered and the solution evaporated to dryness, giving the desired compounds. Yield 55%; mp = 158–162 °C, ethanol. ^1^H-NMR (400 MHz, DMSO-d_6_) δ 1.29 (t, 3H CH_3_, *J* = 6.8 Hz), 4.26 (q, 2H, CH_2_, *J* = 7.2 Hz), 6.91 (m, 3H, H7, and NH_2_, exch.), 7.30 (s, 1H, H9), 7.84 (d, 1H, H6, *J* = 8.8 Hz), 8.42 (s, 1H, H2), 8.86 (s, 1H, H5). ^13^C-NMR (400 MHz, DMSO-d_6_) δ 14.81, 60.16, 93.57, 103.34, 110.61, 115.91, 131.48, 138.12, 145.28, 145.59, 154.04, 156.04, 162.83. IR nujiol cm^−^^1^: 3360, 3196, 1713, 1587, 1263, 1111. Anal. C_13_H_12_N_4_O_2_ (C, H, N).

**8-Aminopyrazolo[1,5-a]quinazoline-3-carboxylic acid, 16:** The starting product **15** was suspended in a NaOH 10% solution and maintained at 150 °C for 8 h. When the reaction ended, evaluated by TLC (eluent toluene/ethyl acetate/methanol, 8:2:1.5, *v*/*v*/*v*), the solution was treated with ice/water and then acidified by acetic acid until pH 5–6. The precipitate was filtered and washed with ethanol and diethyl ether. Yield 97%; mp > 300 °C. ^1^H-NMR (400 MHz, DMSO-d_6_) δ 6.71 (m, 3H, NH_2_, exch., H7), 7.26 (s, 1H, H9), 7.77 (d, 1H, H6, *J* = 8.0 Hz), 8.11 (s, 1H, H2), 8.65 (s, 1H, H5). Anal. C_11_H_8_N_4_O_2_ (C, H, N).

**General procedure for the synthesis of compounds 17a,b:** The carboxylic acid **16** (0.5 mmol) was treated with SOCl_2_ and maintained at reflux temperature for 1 h; after the evaporation of the excess reagent, DCM (10 mL) and the suitable alcohol (0.7 mmol) were added. The reaction was monitored by TLC (eluent toluene/ethyl acetate/acetic acid, 8:2:1, *v*/*v*/*v*) and at the end, the solvent evaporated, and the residue was treated with an alkaline aqueous solution and the precipitate was filtered and recrystallized by ethanol.

**2-Methoxybenzyl 8-aminopyrazolo[1,5-a]quinazoline-3-carboxylate, 17a**

From **16** and 2-methoxybenzyl alcohol; yield 20%; mp = 182–185 °C. ^1^H-NMR (400 MHz, CD_3_CN-d_3_) δ 3.85 (s, 3H, OCH_3_), 5.37 (s, 2H, CH_2_), 6.96–7.03 (m, 3H, Ph), 7.33 (m, 1H, H7), 7.44 (bs, 2H, NH_2_, exch.), 7.51 (m, 2H, H9 and Ph), 7.84 (d, 1H, H-6, *J* = 8.8), 8.43 (s, 1H, H2), 8.86 (s, 1H, H5). ^13^C-NMR (400 MHz, DMSO-d_6_) δ 14.85, 60.50, 110.71, 121.04, 132.30, 146.46, 156.06. Anal. C_19_H_16_N_4_O_3_ (C, H, N).

**Thien-2-ylmethyl 8-aminopyrazolo[1,5-a]quinazoline-3-carboxylate, 17b**

From **16** and 2-thienylmethanol; yield 30%; mp = 178–181 °C. ^1^H-NMR (400 MHz, CD_3_CN-d_3_) δ 5.49 (s, 2H, CH_2_), 6.93 (m, 3H, H7 and H3, H4 thiophene), 7.21 (m, 1H, H5 thiophene), 7.40 (m, 3H, H9, NH_2_, exch.), 7.80 (d, 1H, H6, *J* = 8.4), 8.39 (s, 1H, H2), 8.81 (s, 1H, H5). ^13^C-NMR (400 MHz, DMSO-d_6_) δ 59.91, 93.67, 102.79, 110.68, 116.00, 127.25, 127.81, 128.82, 131.32, 138.16, 139.18, 145.37, 145.92, 154.07, 156.28, 162.20. Anal. C_16_H_12_N_4_O_2_S (C, H, N).

**General procedure for the synthesis of compounds 18 and 19:** Compound **15** (0.2 mmol) was suspended in anhydrous DMF (2 mL), and anhydrous K_2_CO_3_ (0.4 mmol) was added, maintaining the suspension under stirring at room temperature for 30 min. After adding benzyl bromide (0.2 mmol), the reaction was maintained at 80 °C until the starting material disappeared. The final solution was treated with water and then extracted with DCM (10 mL × 3vv), and the organic layer was worked up as usual. The residue was purified by a chromatography column (eluent toluene/ethyl acetate/acetic acid, 8:2:1 *v*/*v*/*v*), obtaining the 8-N-benzylamino (18) and the 8-dibenzylamino derivative (19).

**Ethyl 8-benzylaminopyrazolo[1,5-a]quinazoline-3-carboxylate, 18**

The second band running, yield 25%, mp = 175–176° C. ^1^H-NMR (400 MHz, DMSO-d_6_) δ 1.28 (t, 3H, CH_3_, *J* = 6.8 Hz); 4.25 (q, 2H, CH_2_, *J* = 7.2 Hz); 4.50 (d, 2H, CH_2_, *J* = 6.0 Hz); 7.02 (d, 1H, H7, *J* = 8.8 Hz); 7.13 (s, 1H, H9); 7.21 (t, 1H benzyl, *J* = 6.0 Hz); 7.29–7.35 (m, 4H, benzyl); 7.86 (d, 1H, H6, *J* = 8.0 Hz), 8.39 (s, 1H, H2), 8.85 (s, 1H, H5). ^13^C-NMR (400 MHz, DMSO-d_6_) δ 14.82, 46.15, 60.13 110.75, 127.48, 127.63, 129.08, 130.94, 130.63, 145.30, 153.92, 154.85, 162.75. Anal. C_20_H_18_N_4_O_2_ (C, H, N).

**Ethyl 8-dibenzylaminopyrazolo[1,5-a]quinazoline-3-carboxylate, 19**

The first band running, yield 25%, mp = 168–170° C. ^1^H-NMR (400 MHz, CDCl_3_) δ 1.41 (t, 3H, CH_3_, *J* = 6.8 Hz); 4.25 (q, 2H, CH_2_, *J* = 7.2 Hz); 4.85 (s, 2H, CH_2_, *J* = 6.0 Hz); 7.02 (dd, 1H, H7, *J* = 2.8, Hz *J* = 8.8 Hz); 7.23–7.34 (m, 5H, benzyl); 7.66 (s, 1H, H9); 7.74(d, 1H, H6, *J* = 8.0 Hz), 8.41 (s, 1H, H2), 8.86 (s, 1H, H5). ^13^C-NMR (400 MHz, DMSO-d_6_) δ 14.82, 46.15, 60.13 110.75, 127.48, 127.63, 129.08, 130.94, 130.63, 145.30, 153.92, 154.85, 162.75. Anal. C_27_H_24_N_4_O_2_ (C, H, N).

### 3.2. Molecular Docking Studies

#### 3.2.1. Molecular Docking

All the 3D structures of the molecules were designed (“DS ViewerPro 6.0 Accelrys Software Inc.”, San Diego, CA, USA). The structure of the binding site was obtained from the Human α1β2γ2-GABAA receptor subtype in complex with GABA and flumazenil, conformation B (PDB ID 6D6T) [2], considering all the amino acids within a distance of about 2 nm from the structure of the flumazenil. The ligands were placed at the binding site through AUTODOCK 4.2 [18]. We collected the minimum number of ligand-binding site complex conformations to cover at least 90% of the poses found by AUTODOCK.

#### 3.2.2. Complex Potential Energy Minimization

For the potential energy minimization of the complexes’ ligand–enzyme, we used the GROMACS v5.1 programme and conducted it in vacuum [20].

The partial atomic charge of the ligand structures was calculated with CHIMERA using the AM1-BCC method, and the topology was created with ACPYPE based on the routine Antechamber. For the potential energy calculation, the AMBER99sb force field parameters were applied. A conjugate gradient algorithm for energy minimization, with the tolerance of 10.0 kJ mol^−1^ nm^−1^ (the minimization is converged when the maximum force is smaller than this value), was used.

#### 3.2.3. Molecular Dynamics Simulation

A 60 ns MD simulation was performed for all complexes using the GROMACS v5.1 programme and conducted in vacuum [20]. The partial atomic charge of the ligand structures was calculated with CHIMERA [21] using the AM1-BCC method, and the topology was created with ACPYPE [22] based on the routine Antechamber [23]. The OPLS-AA/L all-atom force field [24] parameters were applied to all the structures. To remove bad contacts, the energy minimization was performed using the steepest descent algorithm until convergence was achieved or for 50,000 maximum steps. The next equilibration of the system was conducted in two phases:

(1) A canonical NVT ensemble, a 100 ps position restraint of molecules at 300 K, was carried out using a temperature coupling thermostat (velocity rescaling with a stochastic term) to ensure the proper stabilization of the temperature [25].
(2) An isothermal isobaric NPT ensemble, a 100 ps position restraint of molecules at 300 K and 1 bar, was carried out without using barostat pressure coupling to stabilize the system. These were then followed by a 60 ns MD run at 300 K with position restraints for all protein atoms. The Lincs algorithm [26] was used for bond constraints to maintain rigid bond lengths.

The initial velocity was randomly assigned, taken from Maxwell–Boltzman distribution at 300 K, and computed with a time step of 2 fs, and the coordinates were recorded every 0.6 ns for an MD simulation of 60 ns. The conformations collected during the simulated trajectory were 100 in total. The ‘Proximity Frequencies’ (PFs) [13] with which the 100 conformations of each binding site ligand complex intercept two or more amino acids during the dynamics simulation have been calculated. The ‘Proximity Frequency’ (PF) is the frequency with which the ligand was, during the molecular dynamics simulation, at a distance of less than 0.25 nm from an amino acid of the binding site and also, simultaneously, from 2 and 3 amino acids of the binding site.

#### 3.2.4. Hierarchical Cluster Analysis (HCA)

A Hierarchical Cluster Analysis was performed by R [19] using the Euclidean distance and ward D2 minimum variance method.

### 3.3. Biological Experiment

#### 3.3.1. Expression of Human Receptor Subunits

A mixture of pCDM8-based vectors for the a1, b2, or g2L subunits of human GABAA receptors (total of 1.5 ng of DNA, comprising equal amounts of α, β, and γ subunit vectors), or an equal amount of α and β receptors for the expression of α1β2 receptors, was injected into the animal pole of X. laevis oocytes as described [27] with the use of a microdispenser (Drummond Scientific, Broomwall, PA, USA). The injected oocytes were maintained at 13 °C in sterile modified Barth’s solution [MBS: 88 mM NaCl, 1 mM KCl, 10 mM HEPES-NaOH (pH 7.5), 0.82 mM MgSO_4_, 2.4 mM NaHCO_3_, 0.91 mM CaCl_2_, 0.33 mM Ca(NO_3_)_2_] supplemented with streptomycin (10 mg/L), penicillin (10,000 U/L), gentamicin (50 mg/L), theophylline (90 mg/L), and pyruvate (220 mg/L).

#### 3.3.2. Electrophysiology

Electrophysiological measurements were performed in oocytes 2 to 4 days after DNA injection. Oocytes were placed in a rectangular chamber (volume~100 µL) and perfused at a rate of 1.7 mL/min with MBS at room temperature with the use of a roller pump (Cole-Parmer, Chicago, IL, USA) and 18-gauge polyethylene tubing (Clay Adams, Parsippany, NJ, USA). Oocytes were impaled at the animal pole with two glass electrodes (0.5 to 10 MΩ) filled with 3 M KCl and were clamped at −70 mV with the use of an oocyte clamp (model OC725C; Warner Instruments, Hamden, CT). Currents were measured and analyzed with the pClamp 9.2 software (Molecular Devices, Union City, CA, USA). GABA (Sigma, St. Louis, MO, USA) was dissolved in MBS and applied to the oocytes for 30 s. Oocytes were perfused with test drugs for 30 s either in the absence of the agonists or in their presence at the EC5-10 (the concentration of an agonist that induces a peak current equal to 5 to 10% of the maximal current elicited by the maximal concentration of the agonist). Compounds were first dissolved in DMSO at a concentration of 10 mM and then diluted in MBS to the final concentrations. In each experiment, control responses were determined before and 10/15 min after the application of the drug.

#### 3.3.3. Statistics

A statistical analysis was performed on normalized data using the Kruskal–Wallis test followed by Dunn’s post hoc test or the Mann–Whitney test using Graph Pad Prism 7 (Graph Pad Software, Inc., San Diego, CA, USA).

## Figures and Tables

**Table 1:** Hydrogen bond lengths with Amino acids involved in the ligand–receptor interaction.

	phe100 ^a^	his102 ^a^	thr142 ^a^	lys156 ^a^	ser159 ^a^	ser205 ^a^	thr207 ^a^	tyr210 ^a^
6a		2.17				2.55		
6b			2.36			2.08		
6c		2.58	2.83			2.16		
6d						2.08		
6e_1						1.97		
6e_2			2.28			2.62		
6e_3								
6f_1		2.61	2.26			2.4	1.91	
6f_2			2.05			2.16	1.98	
6f_3			2.85					
6f_4		2				1.83		
6g						2.18		
8a	2.61	1.98	1.88			2.04		
8b	2.58	1.97				2.19		
9a	2.81	1.92	1.89			1.91		
9b_1	2.59	2.03	1.92				1.92	
9b_2	2.56	2.75	2.65			1.92		2.83
11a				2.56		1.96		
11b_1		1.98	1.96			1.84		
11b_2		2.31		2.49		2.82	2.59	2.49
11c_1				2.02		1.93		2.08
11c_2						1.97		
11d_1		1.98	1.87				2.96	2.63
11d_2				1.94		1.96		
13	2.71	1.92				2.81		
14		2.46	1.89		1.92			
15		2.33	2.06		2.14	1.93		
17a_1					1.79	2.74		
17a_2	2.71	1.87				2.12		
17a_3	2.54	2.12	2.07			2.52	2.16	
17b_1			1.93			1.86	2.87	
17b_2					1.91	2.44	1.81	
								
m ^b^	2.63	2.19	2.17	2.25	1.94	2.16	2.28	2.51
sd ^c^	0.10	0.28	0.34	0.32	0.15	0.29	0.46	0.32

^a^ Hydrogen bond length, expressed in Angstrom (Å), calculated on all conformations of complex ligand-binding site. ^b^ Average of hydrogen bond length for each residue. ^c^ Standard deviation.

**Table 2:** Hierarchical Cluster Analysis (HCA) for the new compounds.

Compound	HCA ^a^	A ^b^	B ^b^
13	AN	1	1
14	AG	1	1
15	AG	0	0
11a	AN	1	1
11b_1	AG	0	1
11b_2	AN	1	
11c_1	AN	1	1
11c_2	AN	1	
11d_1	AG		
11d_2	AN		
17a_1	AN	1	1
17a_2	AN	1	
17a_3	AG	0	
17b_1	AG	0	1
17b_2	AN	1	
6a	AN	1	1
6b	AG	0	0
6c	AG	0	0
6d	AN	1	1
6e_1	AN	1	1
6e_2	AG	0	
6e_3	AN	1	
6f_1	AG		
6f_2	AG		
6f_3	AN		
6f_4	AN		
6g	AN		
8a	AG	1	1
8b	AN	1	1
9a	AG	1	1
9b_1	AG	0	0
9b_2	AG	0	
Accuracy %		64	76

^a^ Activity predicted by HCA. ^b^ Correspondence with biological results.

## Data Availability

The original contributions presented in the study are included in the article/Supplementary Material, further inquiries can be directed to the corresponding author.

## Supplementary Materials

The following supporting information can be downloaded at: https://www.mdpi.com/article/10.3390/ijms251910840/s1, References [2,13,18,28] are cited in the supplementary materials.
