# Supplementary material for: 3,8-Disubstituted Pyrazolo[1,5-a]quinazoline as GABAA Receptor Modulators: Synthesis, Electrophysiological Assays, and Molecular Modelling Studies
Source: Int J Mol Sci. 2024 Oct 9;25(19):10840. doi: 10.3390/ijms251910840 (PMC11477267; doi:10.3390/ijms251910840)
Supplement: Supplementary file 1 [file ijms-25-10840-s001.zip › ijms-3204981-supplementary.pdf]

Supporting Information

For

3,8-Disubstituted pyrazolo[1,5-a]quinazoline as $GABA_A$ receptor modulators: synthesis, electrophysiological assays and molecular modelling studies

Letizia Crocetti [1], Gabriella Guerrini [1]*, Fabrizio Melani[1], Maria Paola Mascia[2] and Maria Paola Giovannoni[1]

[1]Neurofarba, Pharmaceutical and Nutraceutical Section, University of Florence, Via Ugo Schiff 6, 50019, Sesto Fiorentino, Italy; letizia.crocetti@unifi.it (L.C.); gabriella.guerrini@unifi.it (G.G.); fabrizio.melani@unifi.it (F.M.); mariapaola.giovannoni@unifi.it (M.P.G.).

[2]CNR-Institute of Neuroscience, Cagliari, Cittadella Universitaria, 09042, Monserrato, Italy; mariapaola.mascia@cnr.it (M.P.M.).

*Correspondence: gabriella.guerrini@unifi.it (G.G.); Tel.: Tel.: +39-055-4573766

Table of contents

1. ^{1}H-NMR and ^{13}C-NMR spectra of some representative compounds
2. Electrophysiological results for final compounds **6a-g, 13, 8a,b, 9a,b, 11a-d, 13, 14, 15 and 17a,b (Figure S1)**
3. Molecular modelling studies (Proximity Frequencies evaluation, **Table S1**)

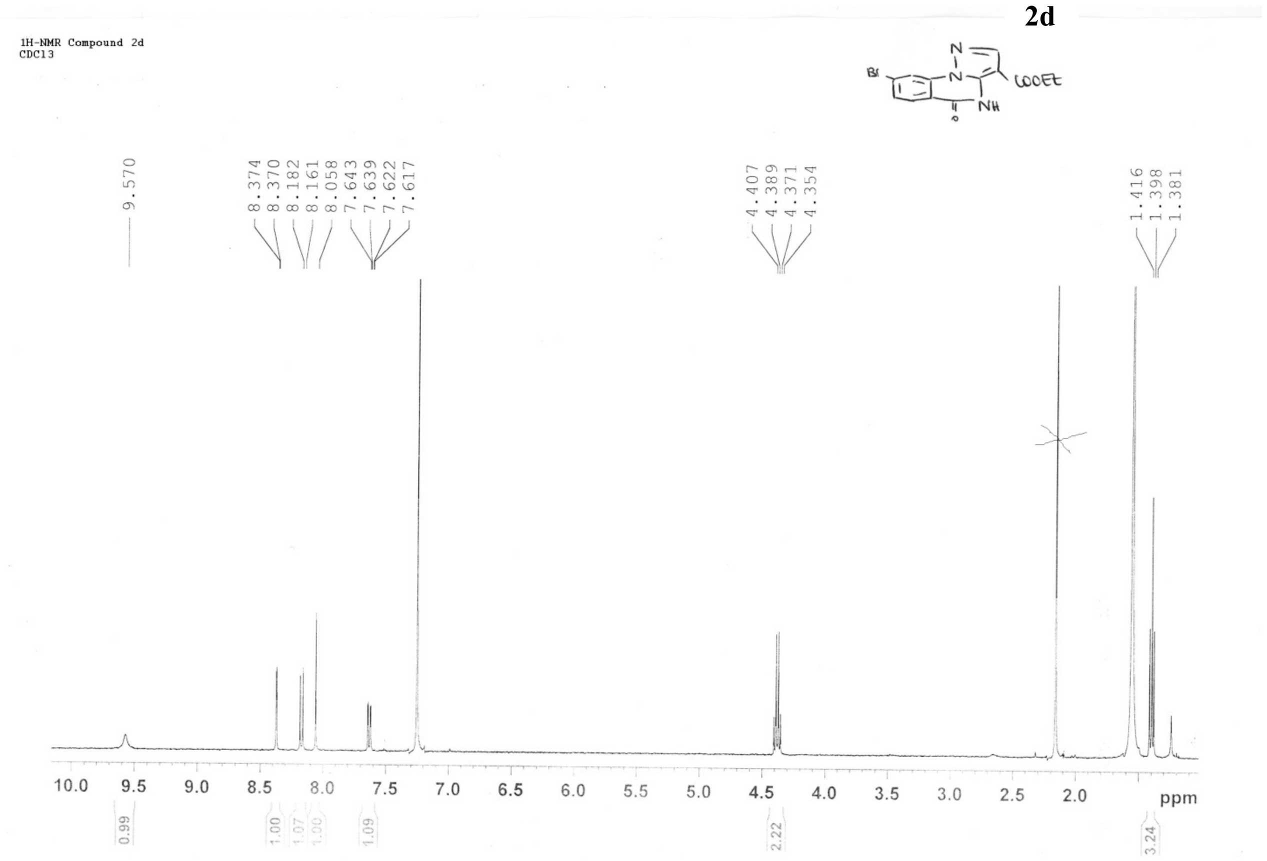
1H-NMR Compound 2d
CDCl3
2d
Br
N
N
COOEt
NH
O
9.570
8.374
8.370
8.182
8.161
8.058
7.643
7.639
7.622
7.617
4.407
4.389
4.371
4.354
1.416
1.398
1.381
10.0
9.5
9.0
8.5
8.0
7.5
7.0
6.5
6.0
5.5
5.0
4.5
4.0
3.5
3.0
2.5
2.0
ppm
0.99
1.00
1.07
1.00
1.09
2.22
3.24

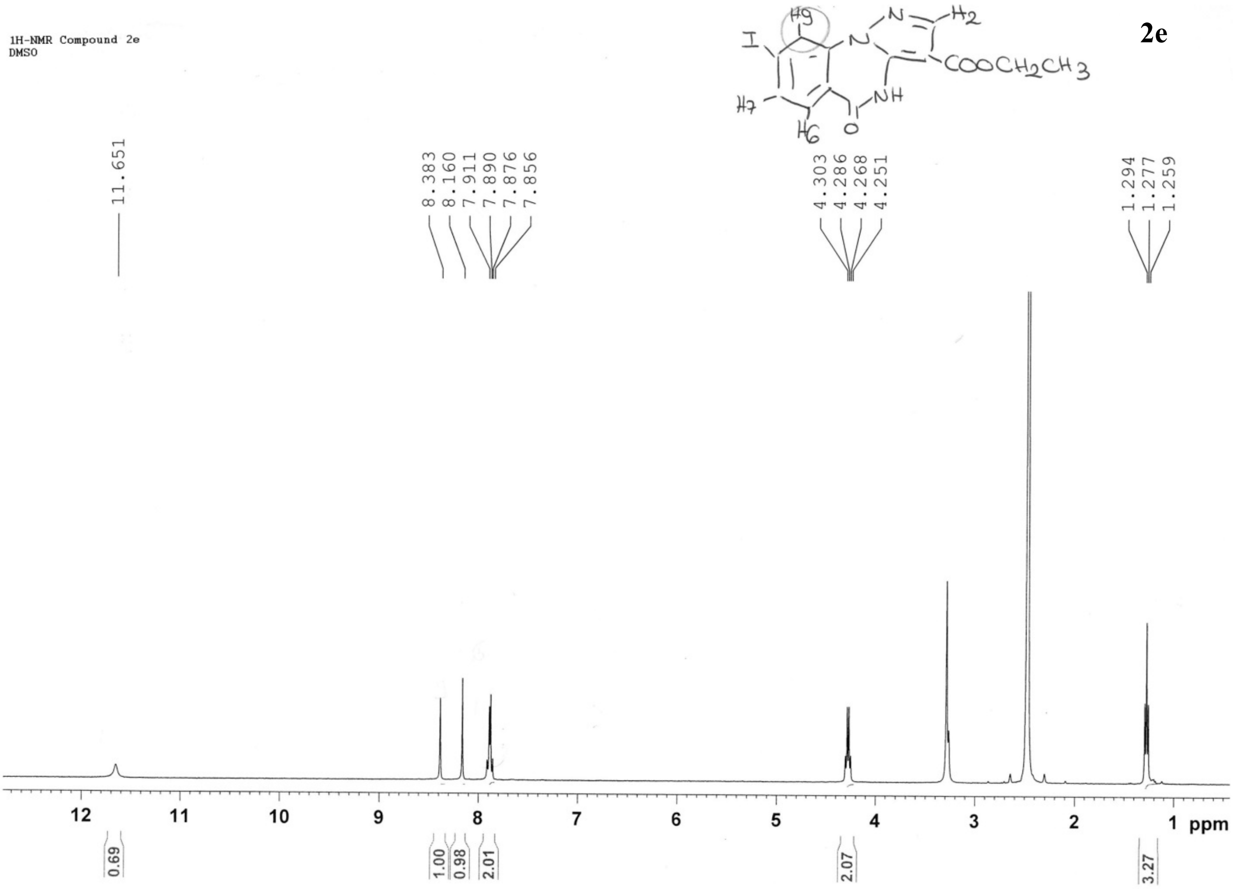
1H-NMR Compound 2e
DMSO
2e
I
H9
N
N
H2
COOCH2CH3
H7
H6
NH
O
11.651
8.383
8.160
7.911
7.890
7.876
7.856
4.303
4.286
4.268
4.251
1.294
1.277
1.259
12
11
10
9
8
7
6
5
4
3
2
1 ppm
0.69
1.00
0.98
2.01
2.07
3.27

1H-NMR Compound 3c
DMSO

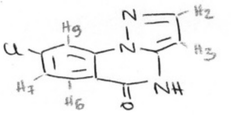

3c

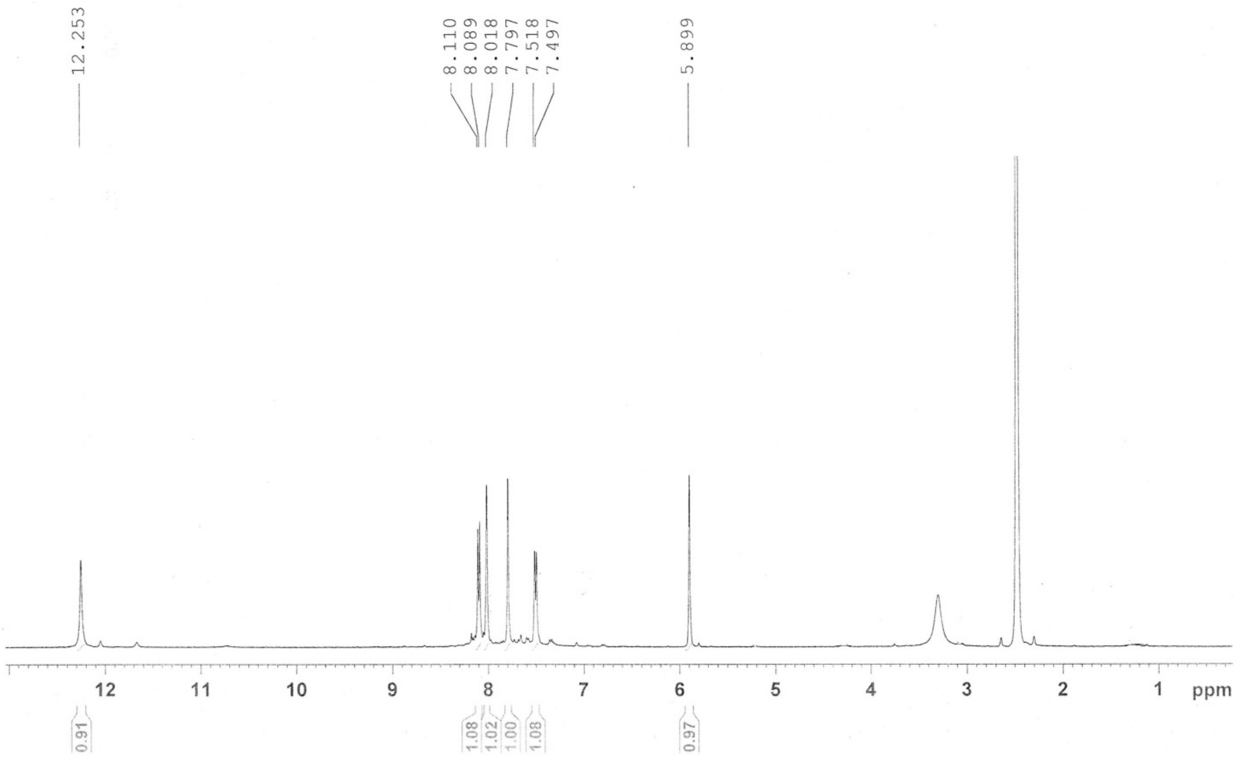

1H-NMR Compound 3e
DMSO

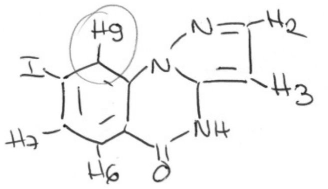

3e

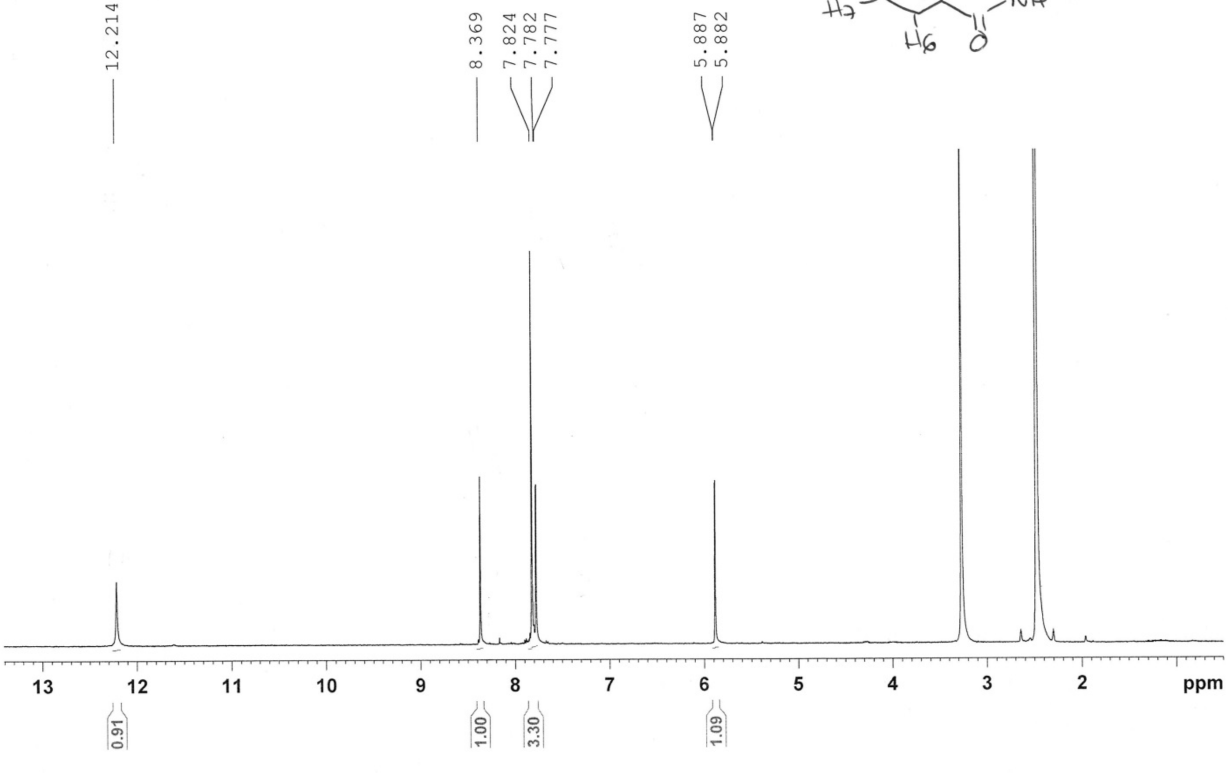

3g

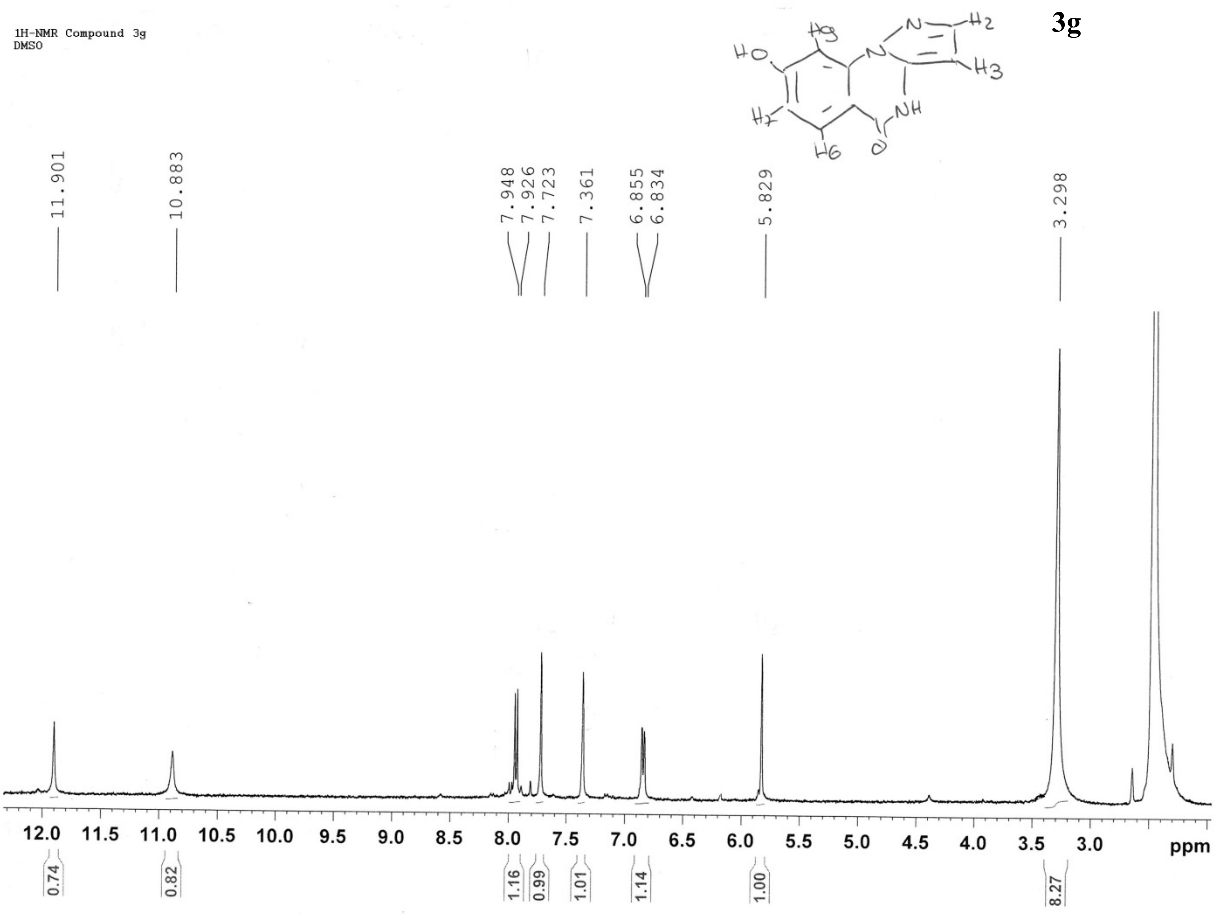
1H-NMR Compound 3g
DMSO
11.901
10.883
7.948
7.926
7.723
7.361
6.855
6.834
5.829
3.298
12.0 11.5 11.0 10.5 10.0 9.5 9.0 8.5 8.0 7.5 7.0 6.5 6.0 5.5 5.0 4.5 4.0 3.5 3.0 ppm
0.74
0.82
1.16
0.99
1.01
1.14
1.00
8.27

4b

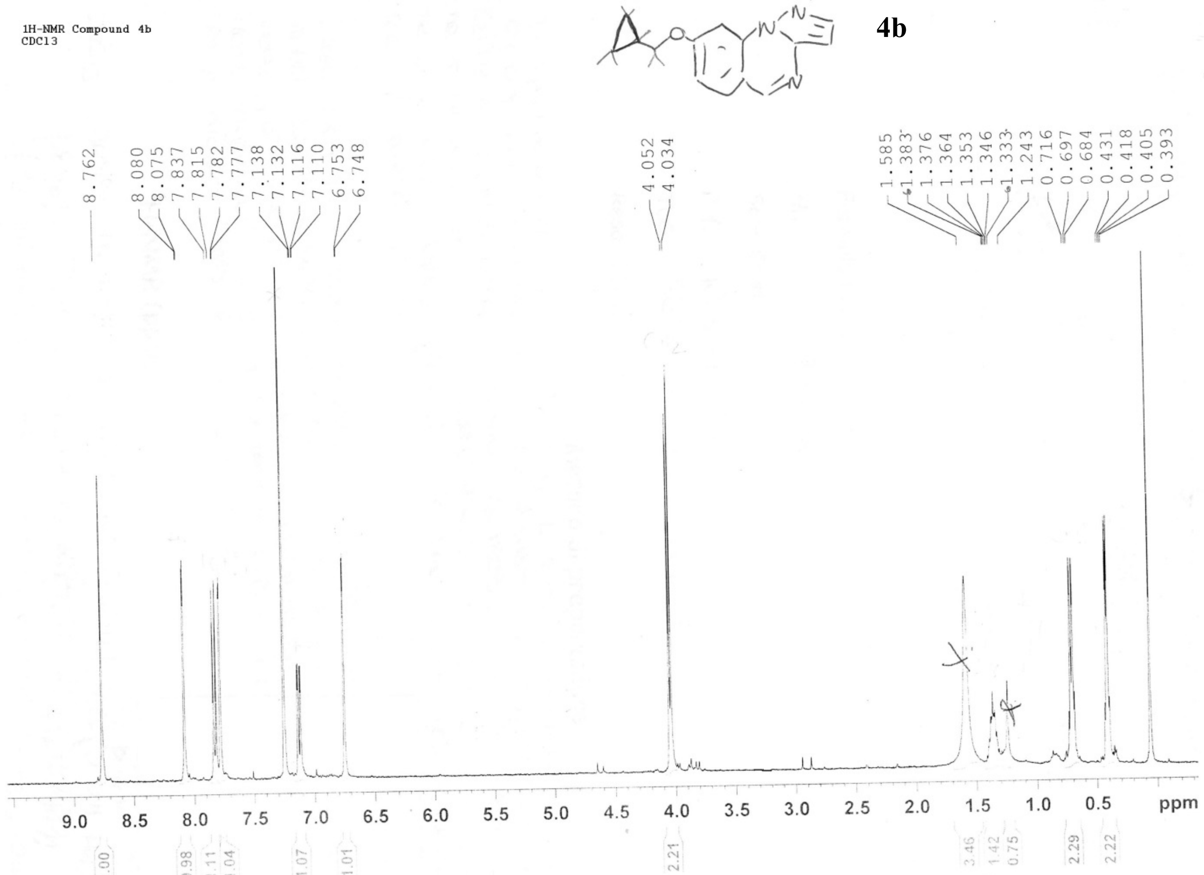
1H-NMR Compound 4b
CDCl3
8.762
8.080
8.075
7.837
7.815
7.782
7.777
7.138
7.132
7.116
7.110
6.753
6.748
4.052
4.034
1.585
1.383
1.376
1.364
1.353
1.346
1.333
1.243
0.716
0.697
0.684
0.431
0.418
0.405
0.393
9.0 8.5 8.0 7.5 7.0 6.5 6.0 5.5 5.0 4.5 4.0 3.5 3.0 2.5 2.0 1.5 1.0 0.5 ppm
1.00
0.98
1.11
1.04
1.07
1.01
2.21
3.46
1.42
0.75
2.29
2.22

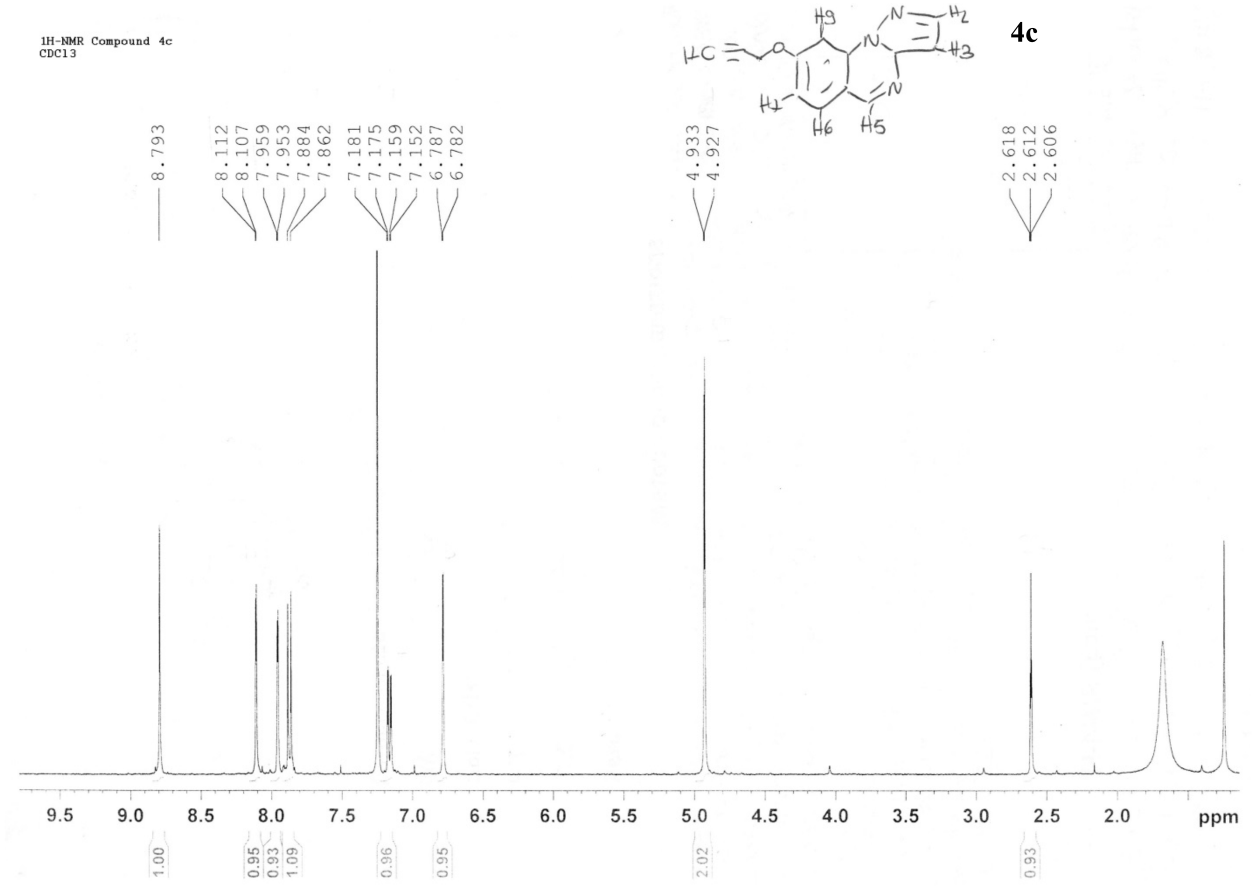
1H-NMR Compound 4c
CDC13
8.793
8.112
8.107
7.959
7.953
7.884
7.862
7.181
7.175
7.159
7.152
6.787
6.782
4.933
4.927
2.618
2.612
2.606
9.5 9.0 8.5 8.0 7.5 7.0 6.5 6.0 5.5 5.0 4.5 4.0 3.5 3.0 2.5 2.0 ppm
1.00 0.95 0.93 1.09 0.96 0.95 2.02 0.93

4c

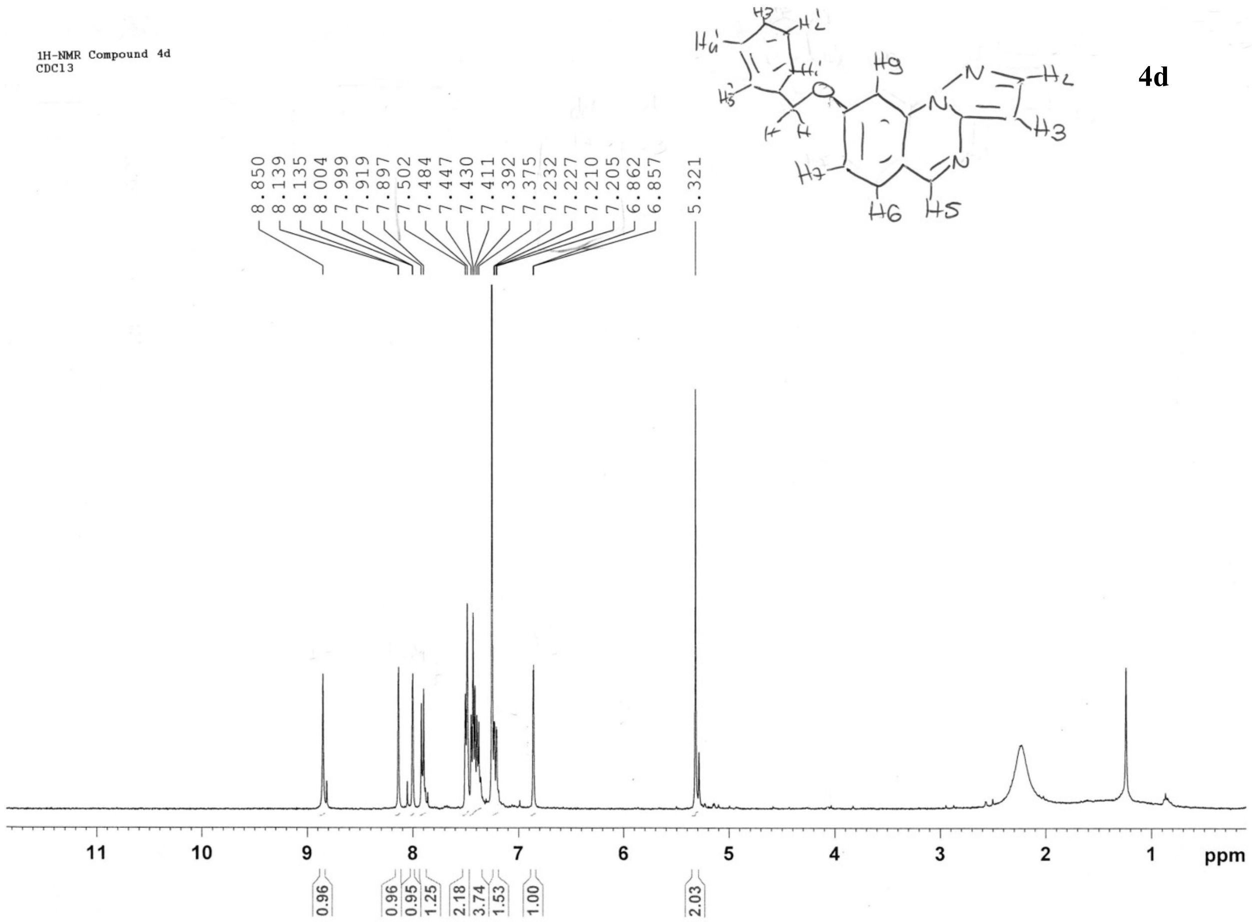
1H-NMR Compound 4d
CDC13
8.850
8.139
8.135
8.004
7.999
7.919
7.897
7.502
7.484
7.447
7.430
7.411
7.392
7.375
7.232
7.227
7.210
7.205
6.862
6.857
5.321
11 10 9 8 7 6 5 4 3 2 1 ppm
0.96 0.96 0.95 1.25 2.18 3.74 1.53 1.00 2.03

4d

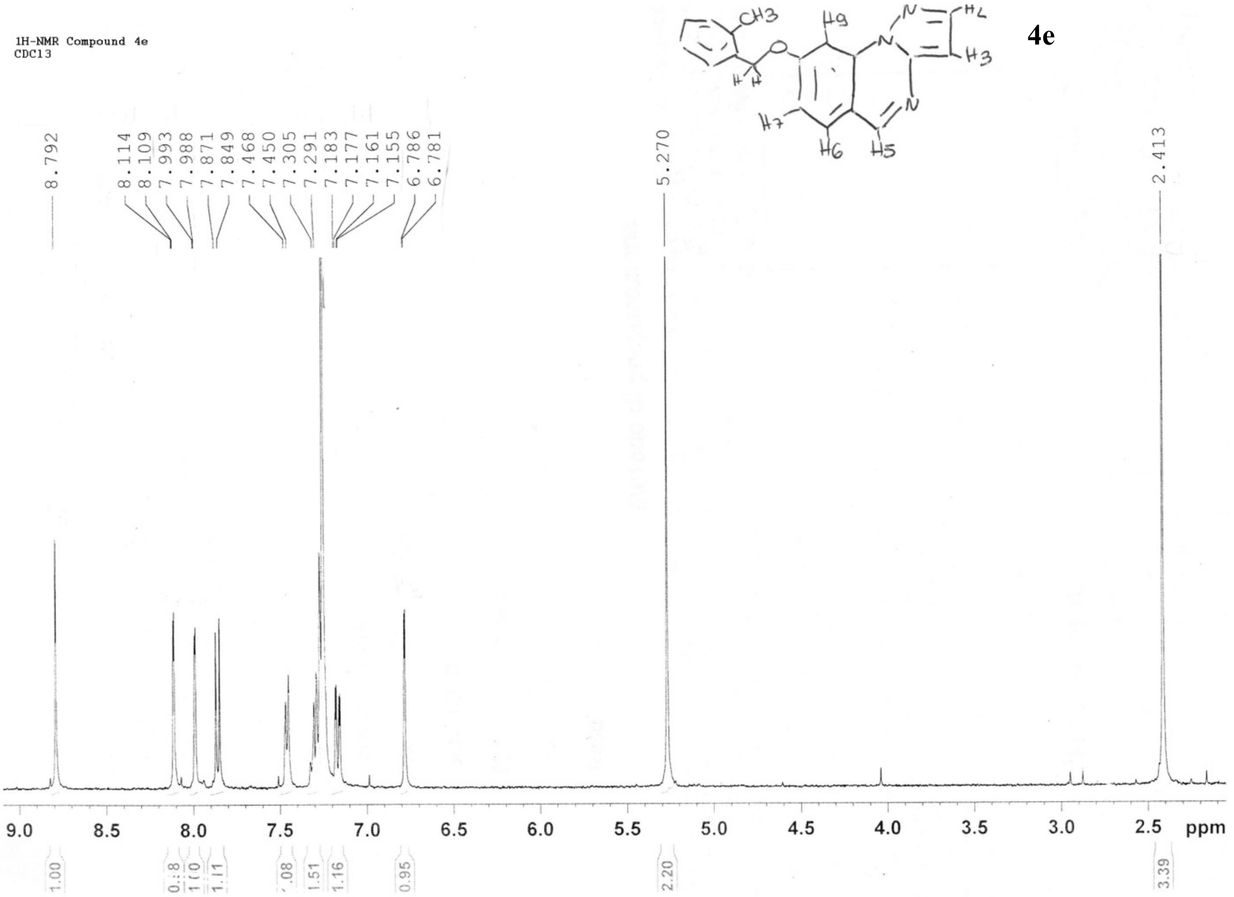
1H-NMR Compound 4e
CDC13
8.792
8.114
8.109
7.993
7.988
7.871
7.849
7.468
7.450
7.305
7.291
7.183
7.177
7.161
7.155
6.786
6.781
5.270
2.413
9.0 8.5 8.0 7.5 7.0 6.5 6.0 5.5 5.0 4.5 4.0 3.5 3.0 2.5 ppm
1.00
[illegible]
[illegible]
1.11
[illegible]
1.51
1.16
0.95
2.20
3.39
CH3
H9
H2
H3
H7
H6
H5

4e

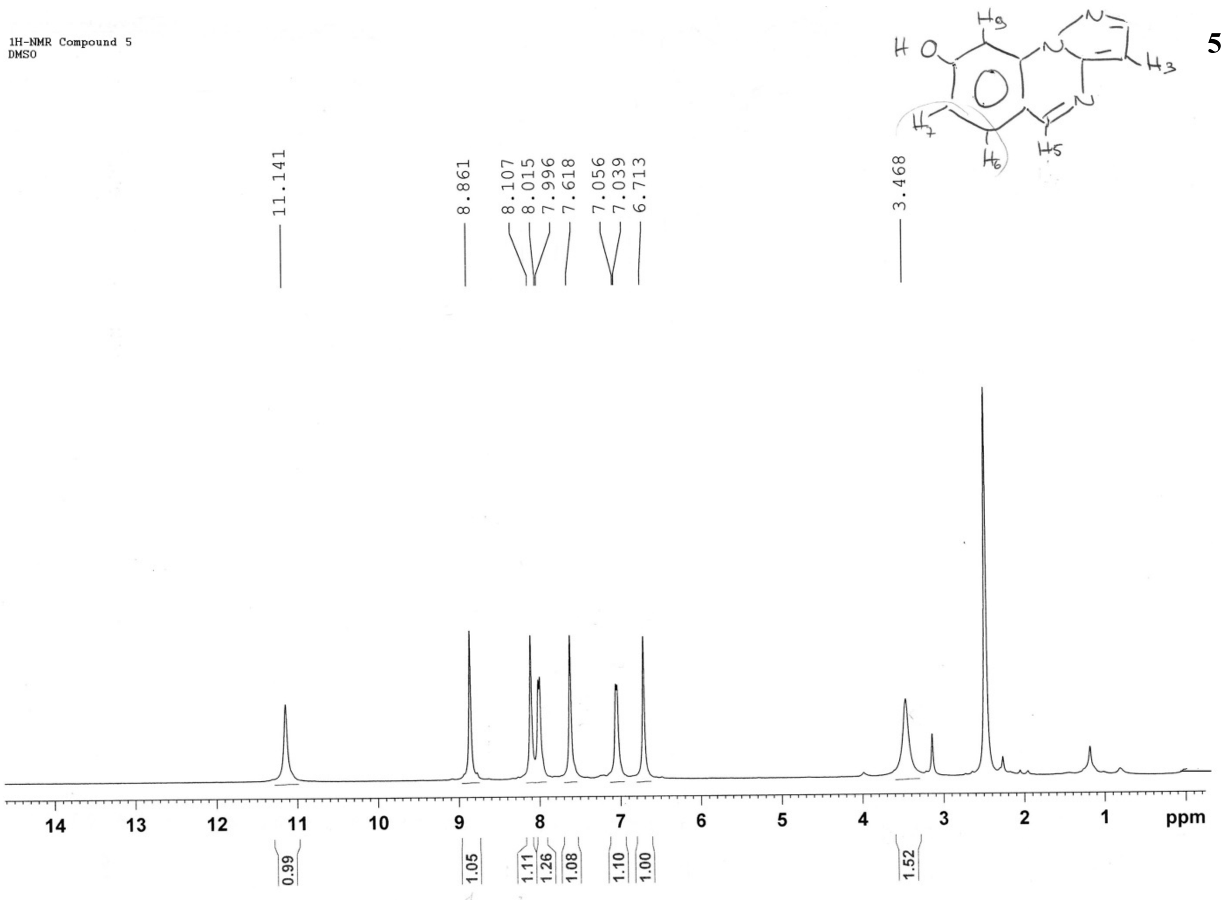
1H-NMR Compound 5
DMSO
11.141
8.861
8.107
8.015
7.996
7.618
7.056
7.039
6.713
3.468
14 13 12 11 10 9 8 7 6 5 4 3 2 1 ppm
0.99
1.05
1.11
1.26
1.08
1.10
1.00
1.52
HO
H9
H3
H7
H6
H5

5

1H-NMR Compound 6b
CDCl3

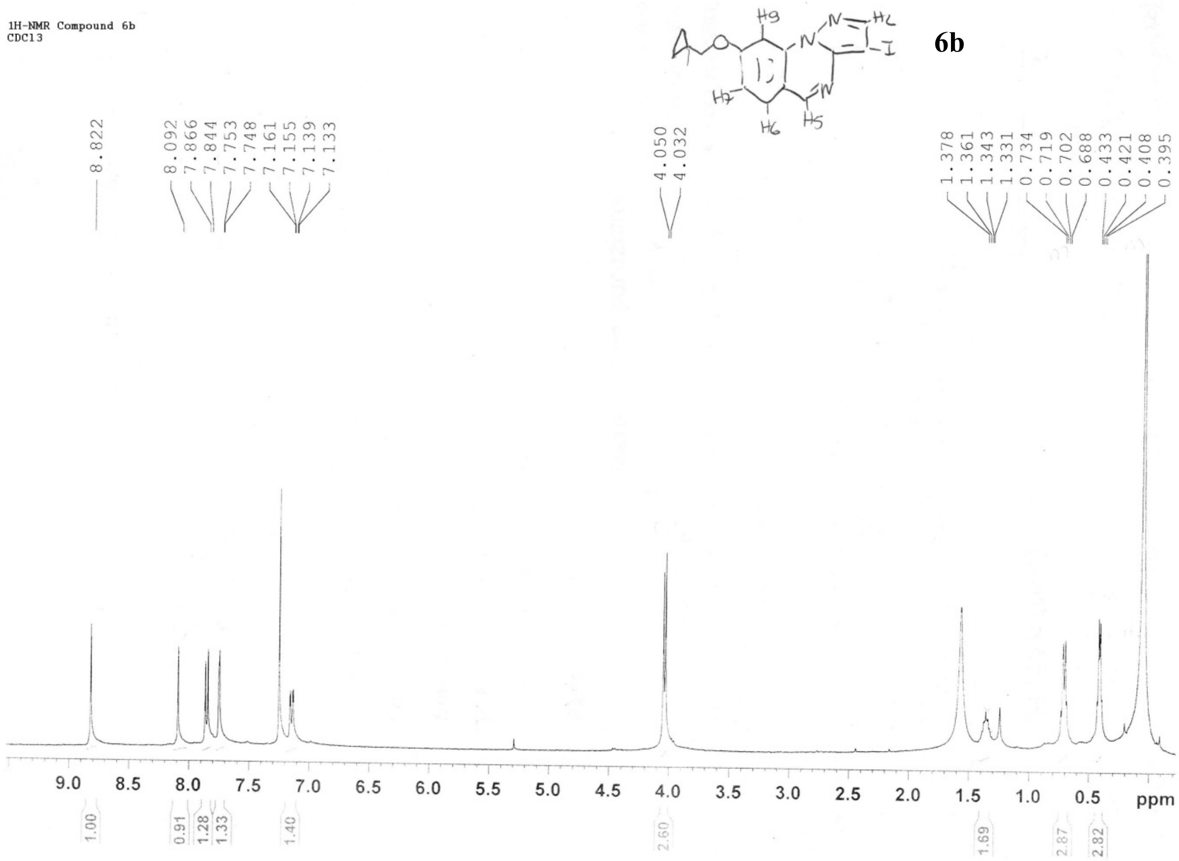

6b

1H-NMR Compound 6c
CDCl3

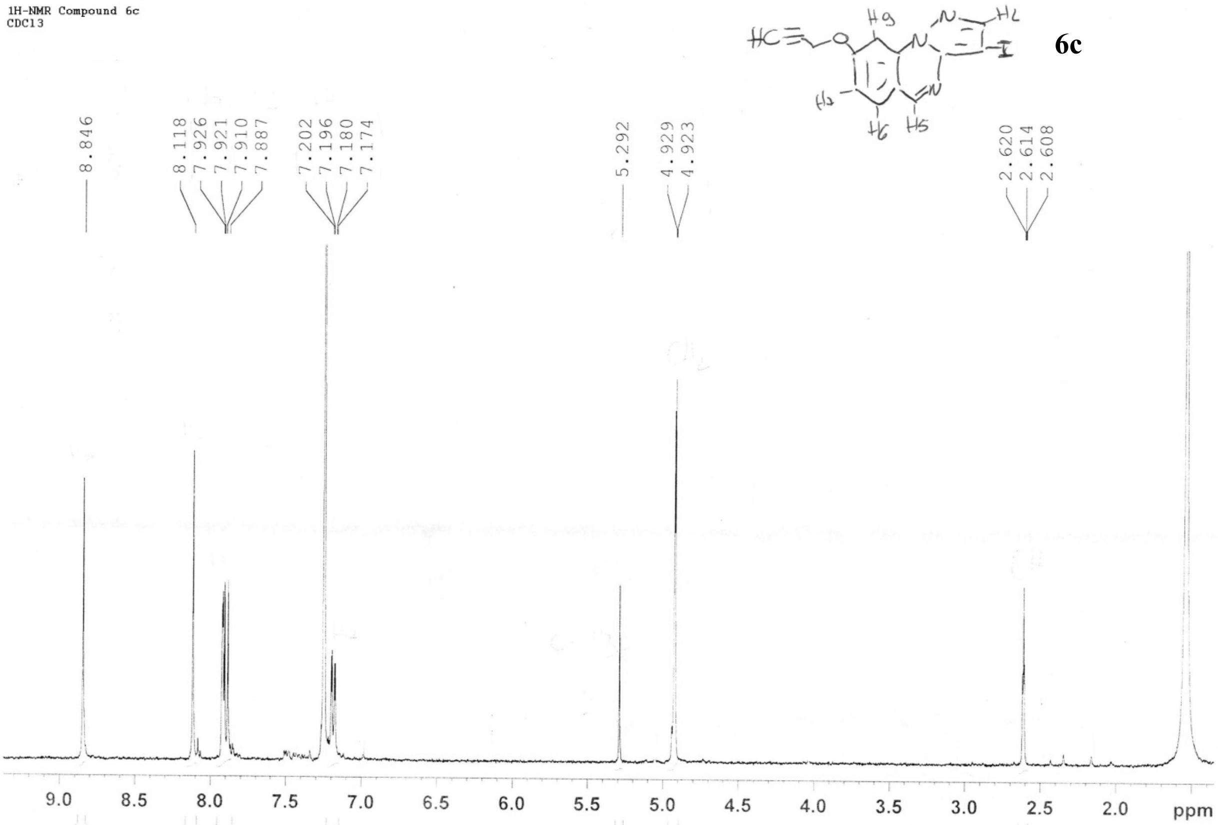

6c

6d

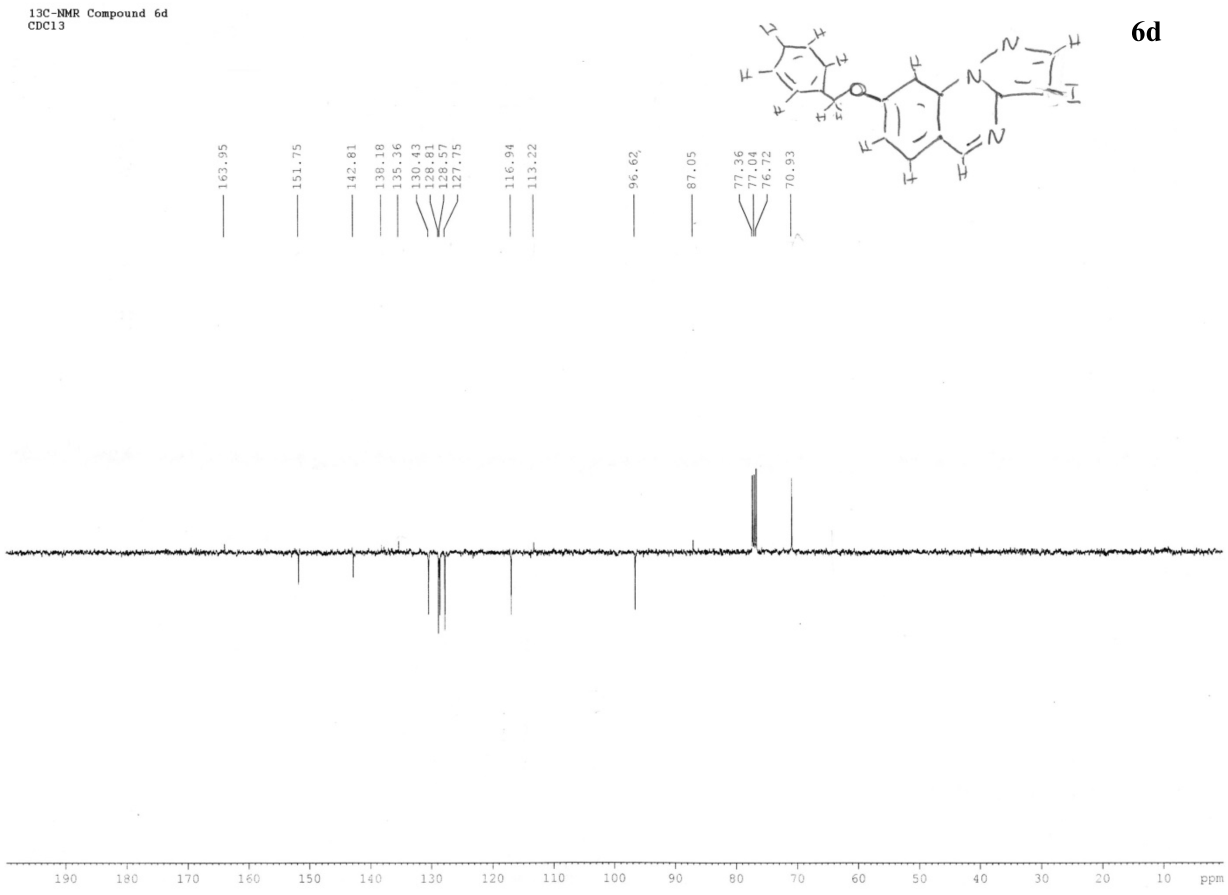
13C-NMR Compound 6d
CDC13
163.95
151.75
142.81
138.18
135.36
130.43
128.81
128.57
127.75
116.94
113.22
96.62
87.05
77.36
77.04
76.72
70.93
190 180 170 160 150 140 130 120 110 100 90 80 70 60 50 40 30 20 10 ppm

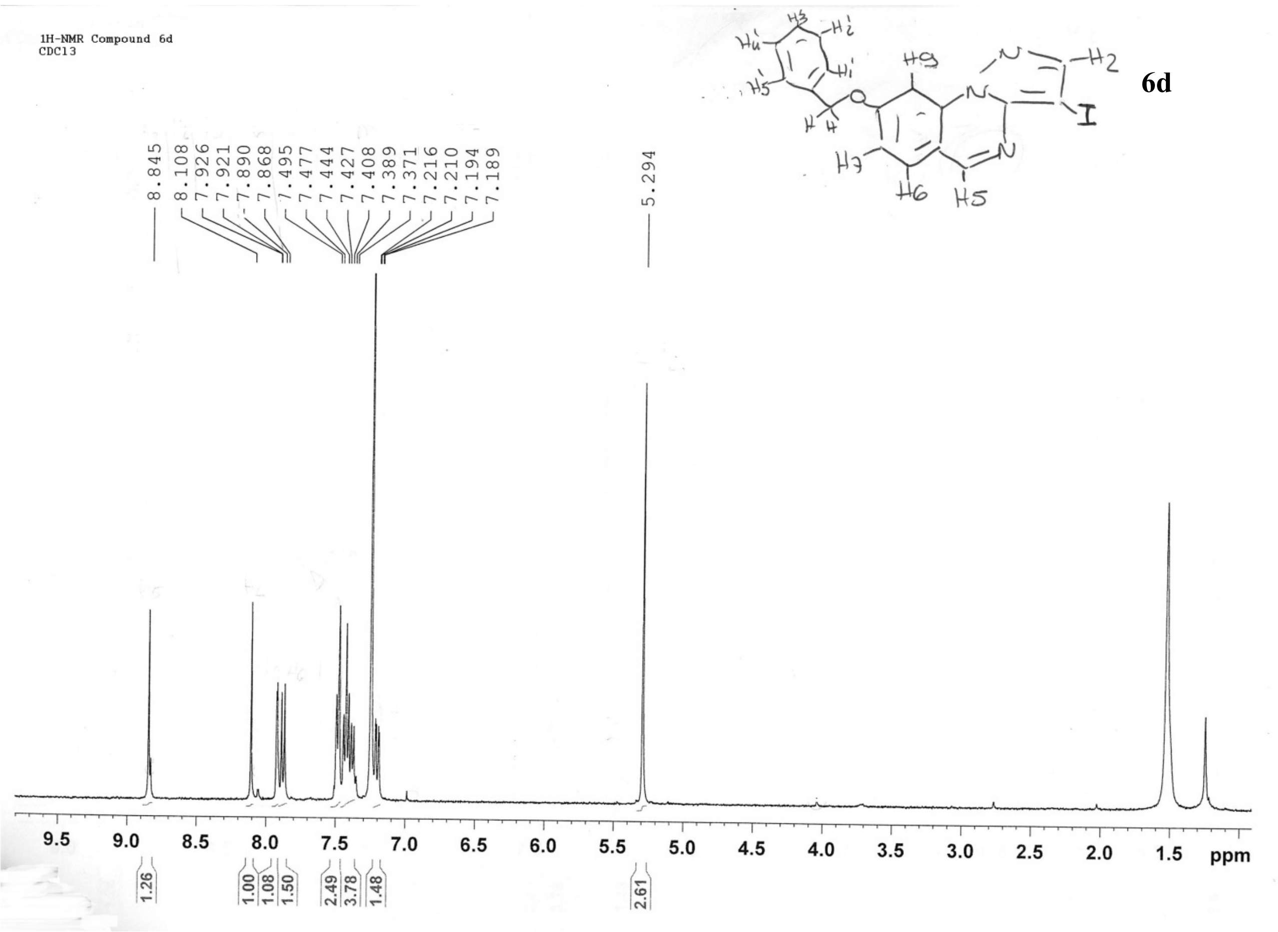
1H-NMR Compound 6d
CDCl3
8.845
8.108
7.926
7.921
7.890
7.868
7.495
7.477
7.444
7.427
7.408
7.389
7.371
7.216
7.210
7.194
7.189
5.294
9.5
9.0
8.5
8.0
7.5
7.0
6.5
6.0
5.5
5.0
4.5
4.0
3.5
3.0
2.5
2.0
1.5
ppm
1.26
1.00
1.08
1.50
2.49
3.78
1.48
2.61

6d

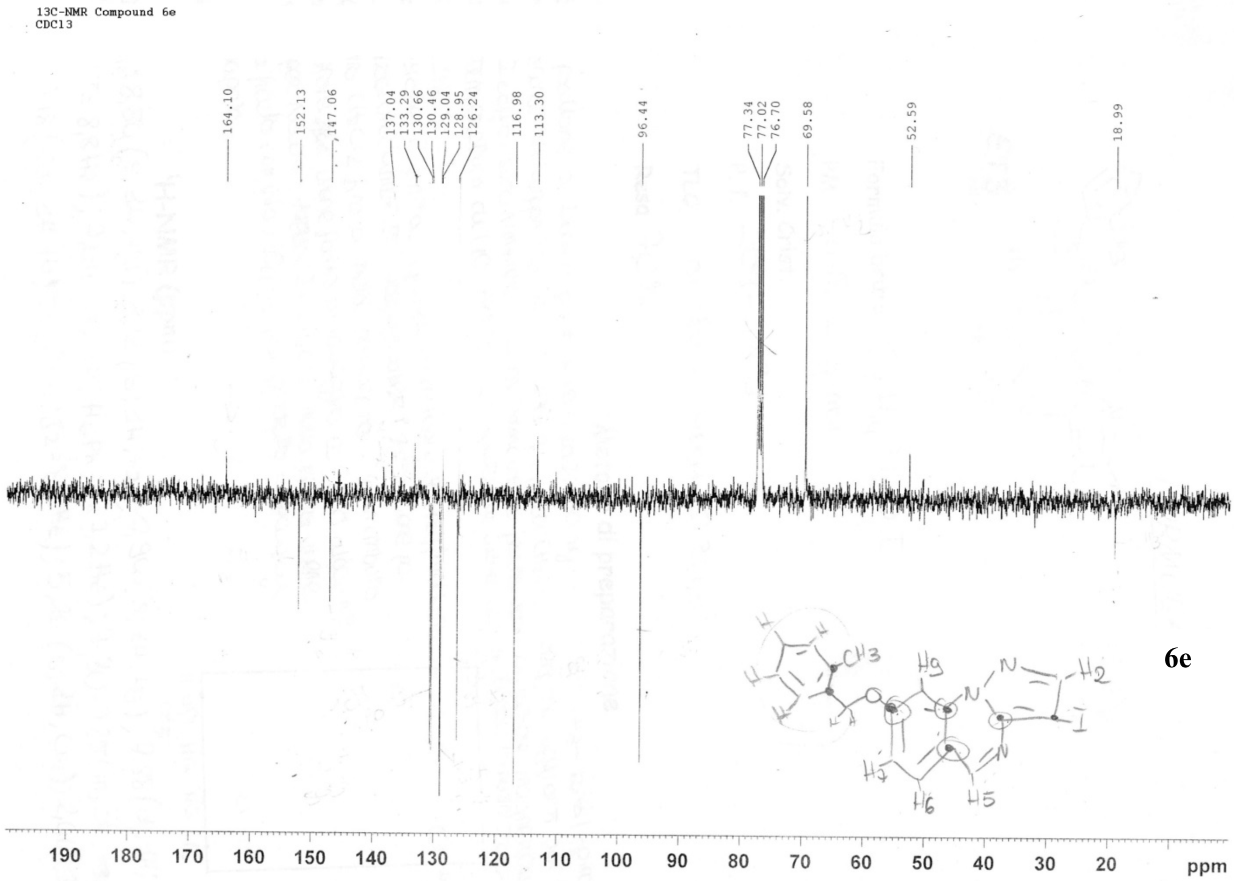
13C-NMR Compound 6e
CDC13
164.10
152.13
147.06
137.04
133.29
130.66
130.46
129.04
128.95
126.24
116.98
113.30
96.44
77.34
77.02
76.70
69.58
52.59
18.99
190 180 170 160 150 140 130 120 110 100 90 80 70 60 50 40 30 20
ppm

6e

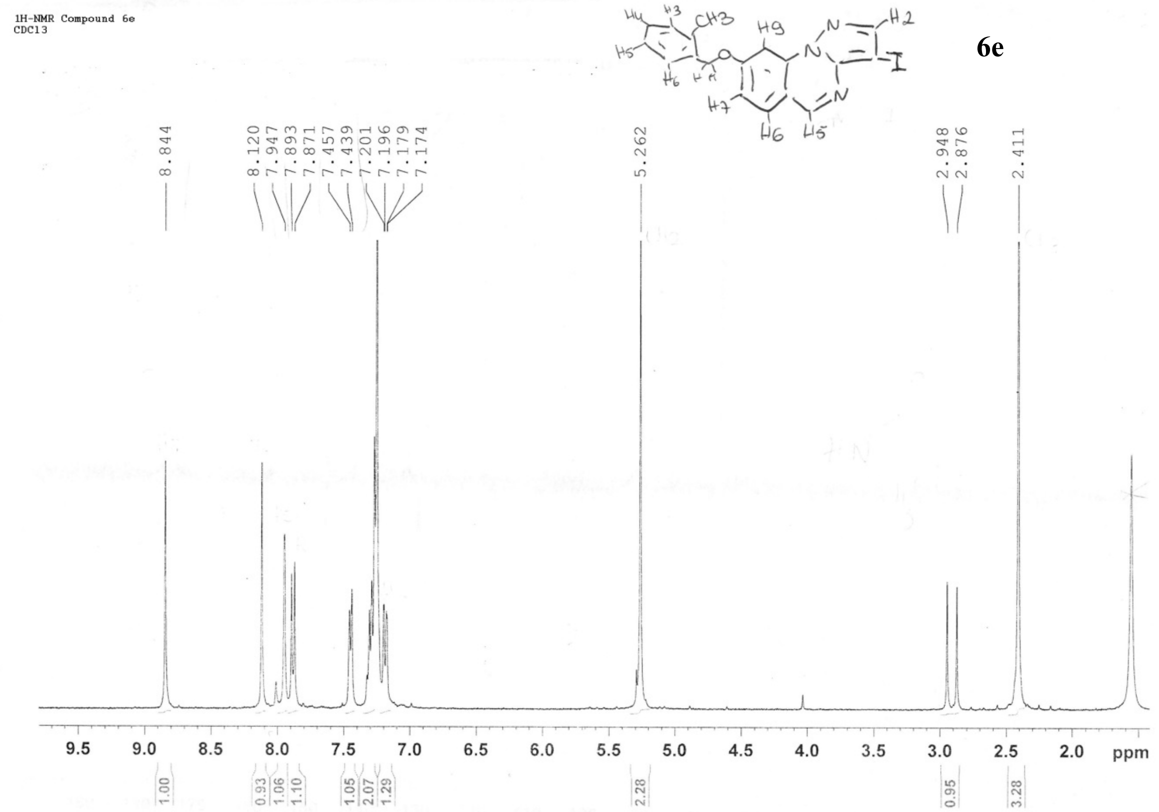
1H-NMR Compound 6e
CDC13
8.844
8.120
7.947
7.893
7.871
7.457
7.439
7.201
7.196
7.179
7.174
5.262
2.948
2.876
2.411
9.5 9.0 8.5 8.0 7.5 7.0 6.5 6.0 5.5 5.0 4.5 4.0 3.5 3.0 2.5 2.0
ppm
1.00
0.93
1.06
1.10
1.05
2.07
1.29
2.28
0.95
3.28

6e

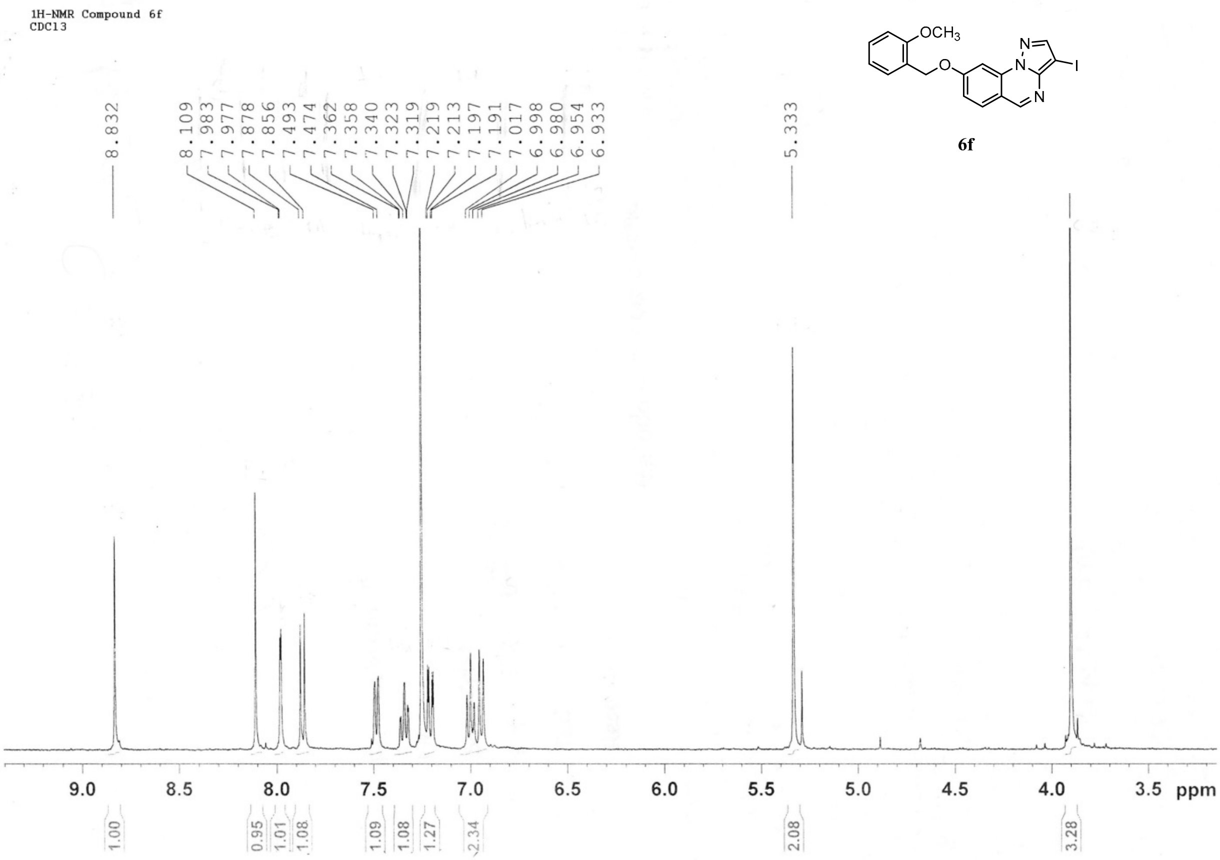
1H-NMR Compound 6f
CDCl3
OCH3
O
N
N
N
I
6f
8.832
8.109
7.983
7.977
7.878
7.856
7.493
7.474
7.362
7.358
7.340
7.323
7.319
7.219
7.213
7.197
7.191
7.017
6.998
6.980
6.954
6.933
5.333
9.0 8.5 8.0 7.5 7.0 6.5 6.0 5.5 5.0 4.5 4.0 3.5 ppm
1.00
0.95
1.01
1.08
1.09
1.08
1.27
2.34
2.08
3.28

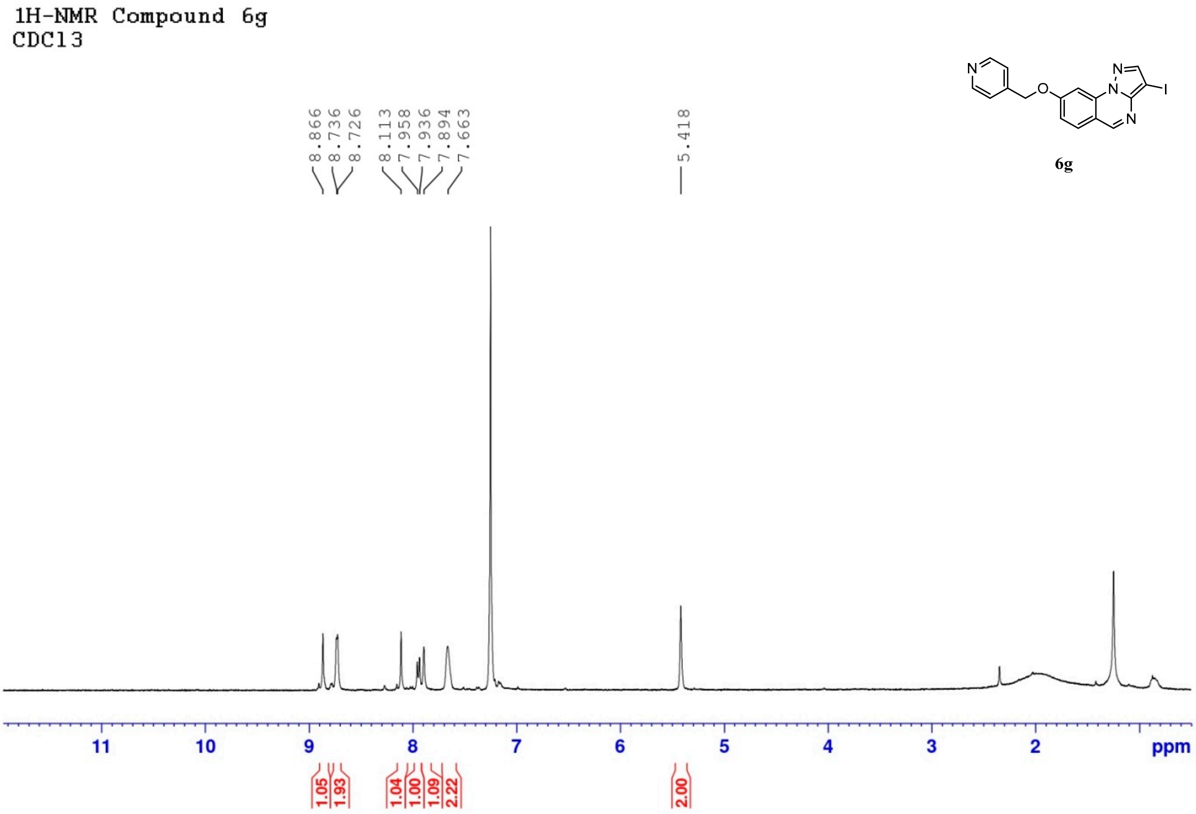
1H-NMR Compound 6g
CDCl3
N
O
N
N
N
I
6g
8.866
8.736
8.726
8.113
7.958
7.936
7.894
7.663
5.418
11 10 9 8 7 6 5 4 3 2 ppm
1.05
1.93
1.04
1.00
1.09
2.22
2.00

13C-NMR Compound 8a
DMSO

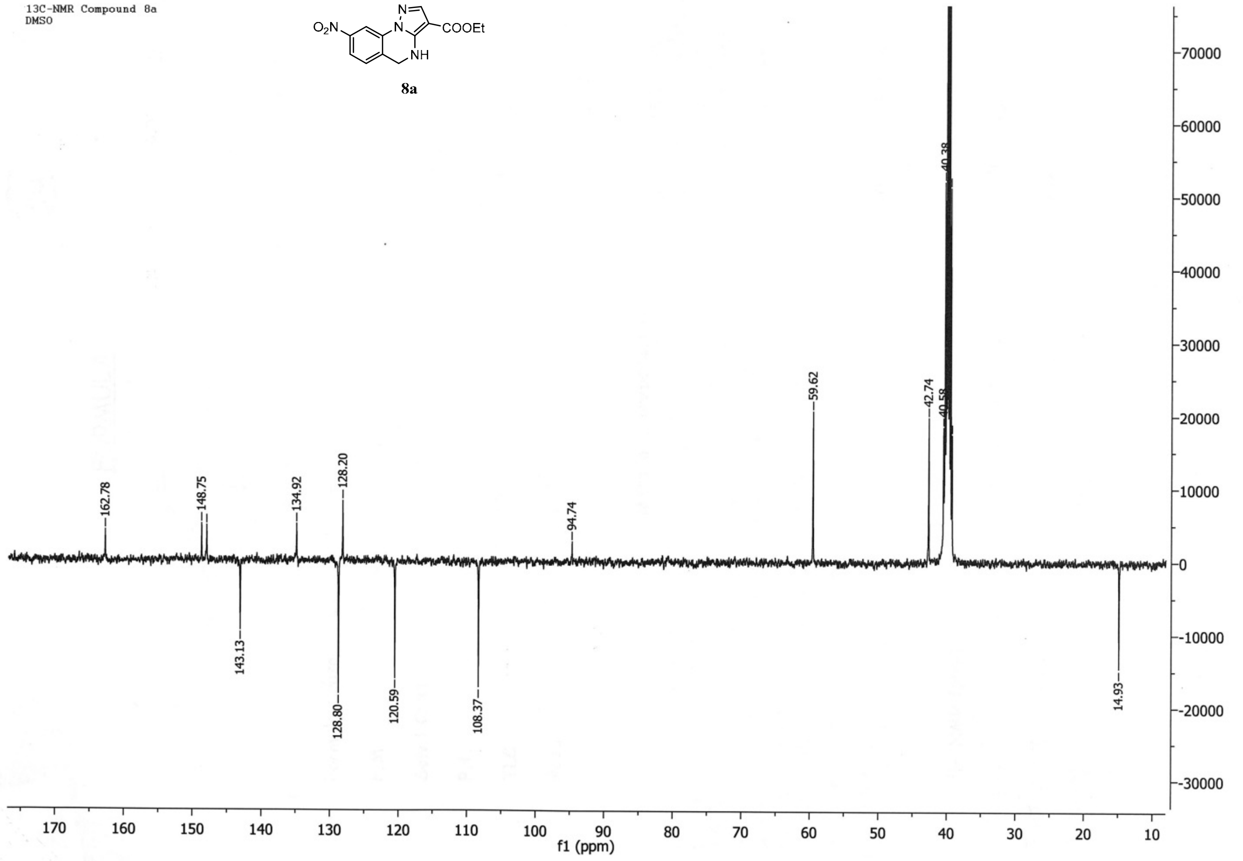

1H-NMR Compound 8a
DMSO

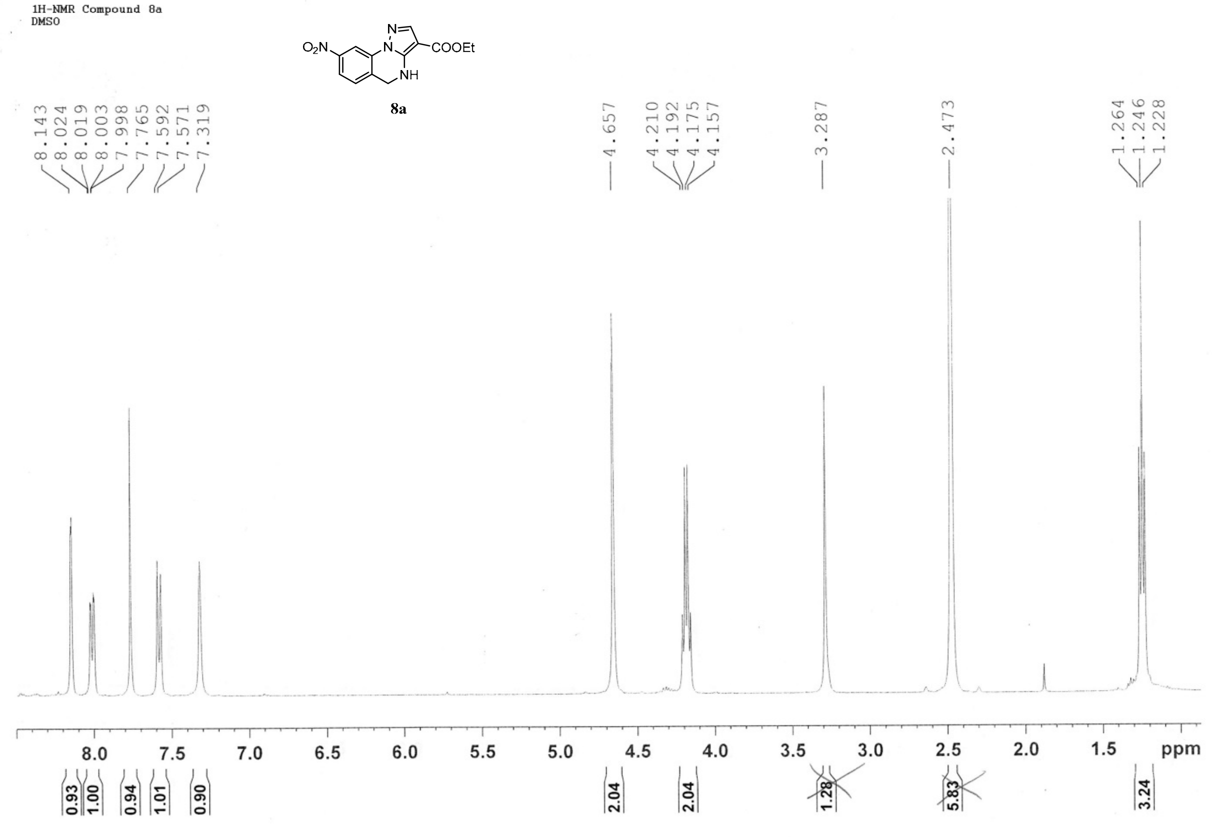

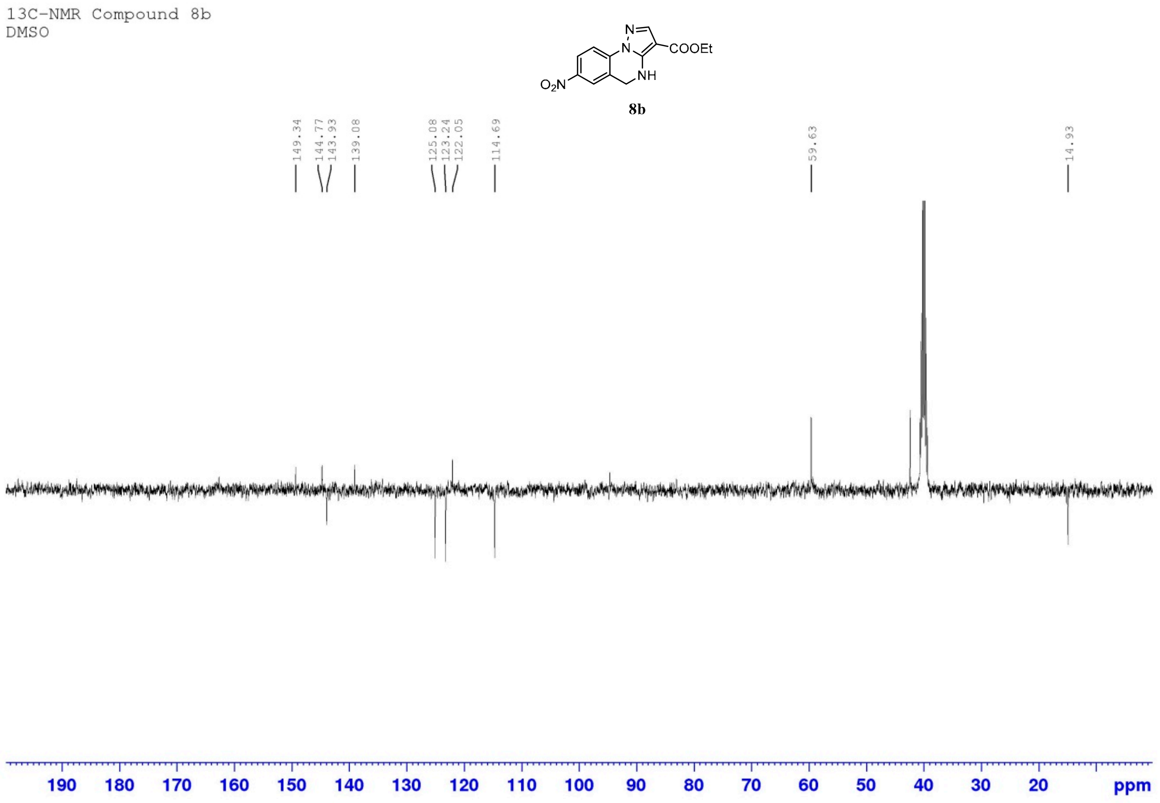
13C-NMR Compound 8b
DMSO
COOEt
NH
O_2N
8b
149.34
144.77
143.93
139.08
125.08
123.24
122.05
114.69
59.63
14.93
190 180 170 160 150 140 130 120 110 100 90 80 70 60 50 40 30 20 ppm

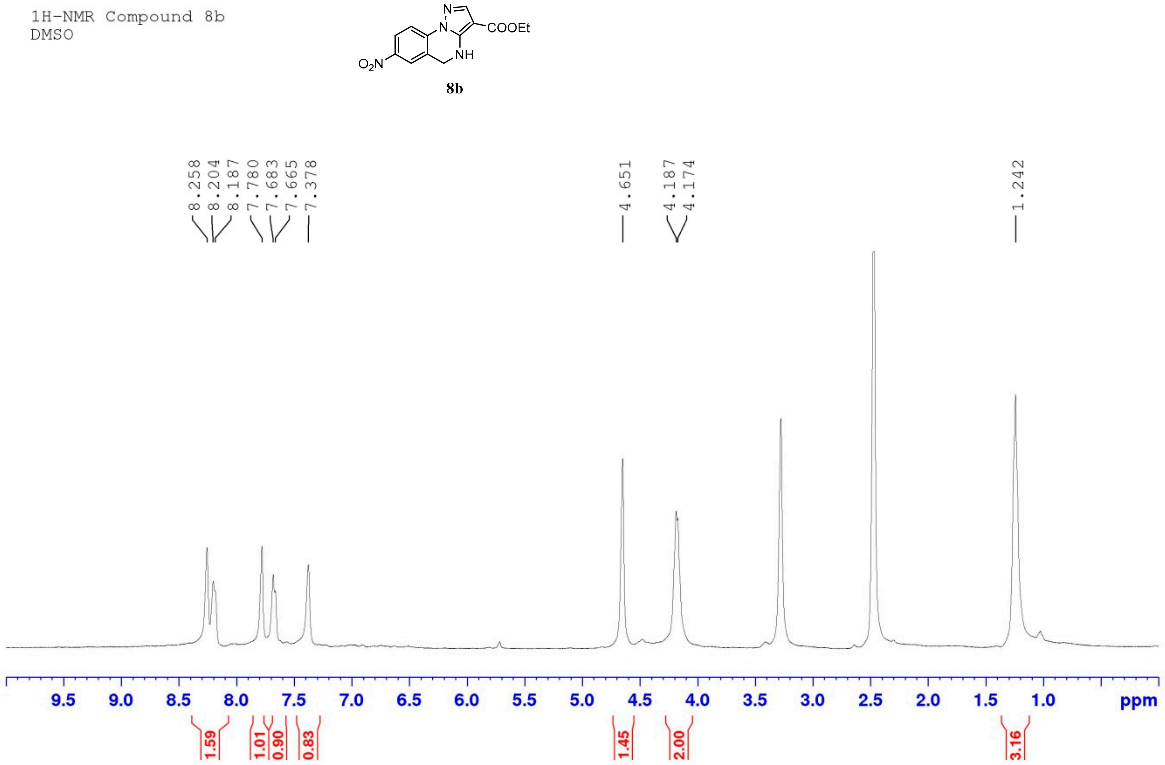
1H-NMR Compound 8b
DMSO
COOEt
NH
O_2N
8b
8.258
8.204
8.187
7.780
7.683
7.665
7.378
4.651
4.187
4.174
1.242
9.5 9.0 8.5 8.0 7.5 7.0 6.5 6.0 5.5 5.0 4.5 4.0 3.5 3.0 2.5 2.0 1.5 1.0 ppm
1.59
1.01
0.90
0.83
1.45
2.00
3.16

1H-NMR Compound 9a
DMSO

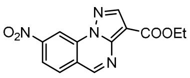

9a

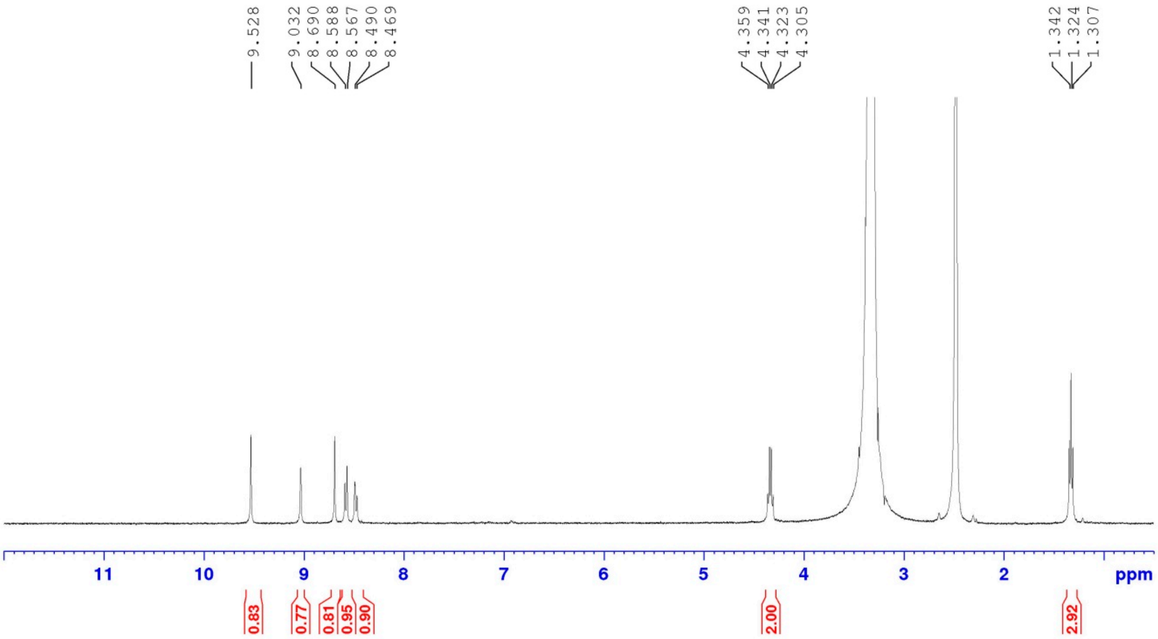

13C-NMR Compound 9b
DMSO

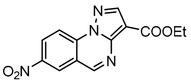

9b

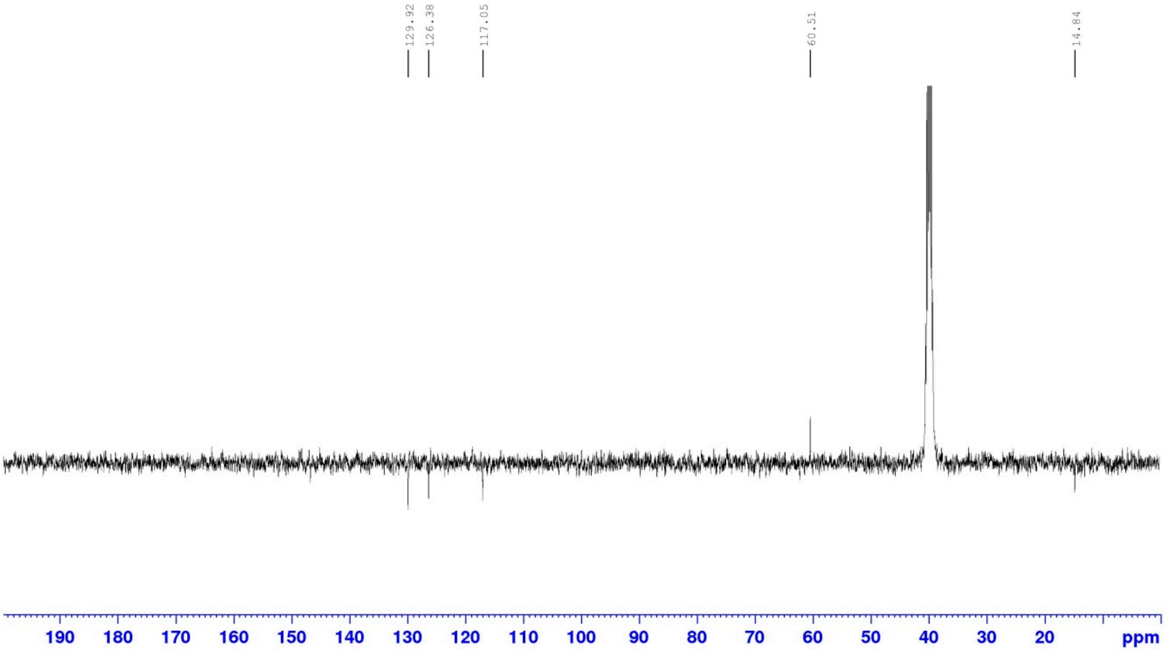

1H-NMR Compound 9b
DMSO

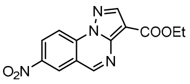

9b

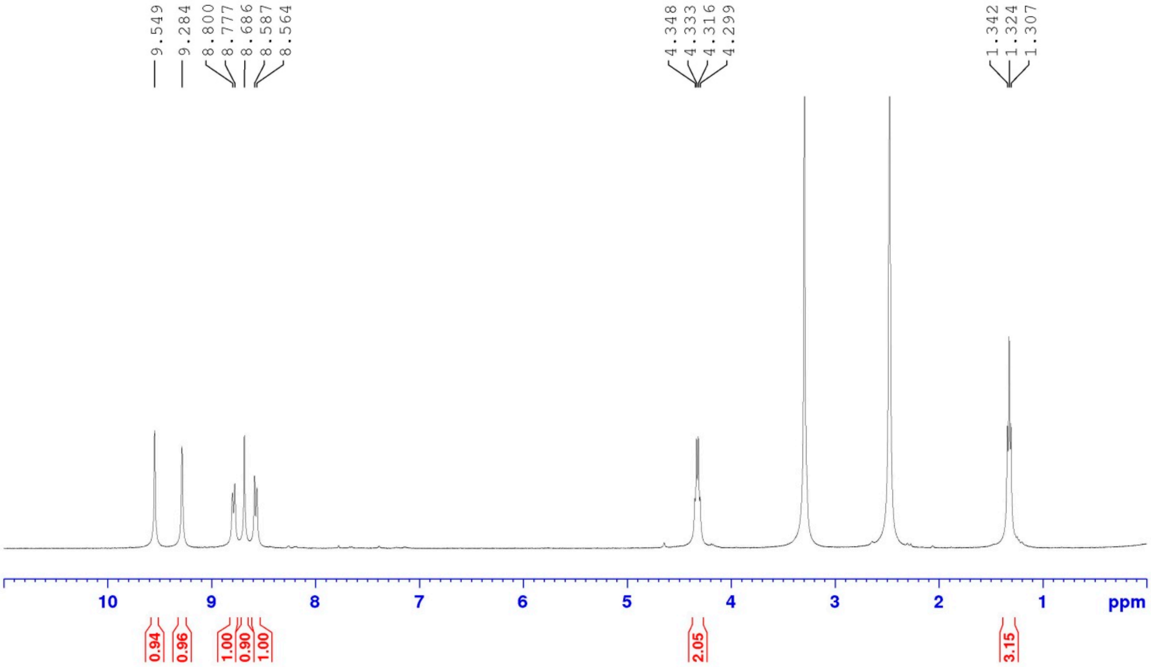

1H-NMR Compound 10a
DMSO

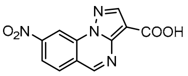

10a

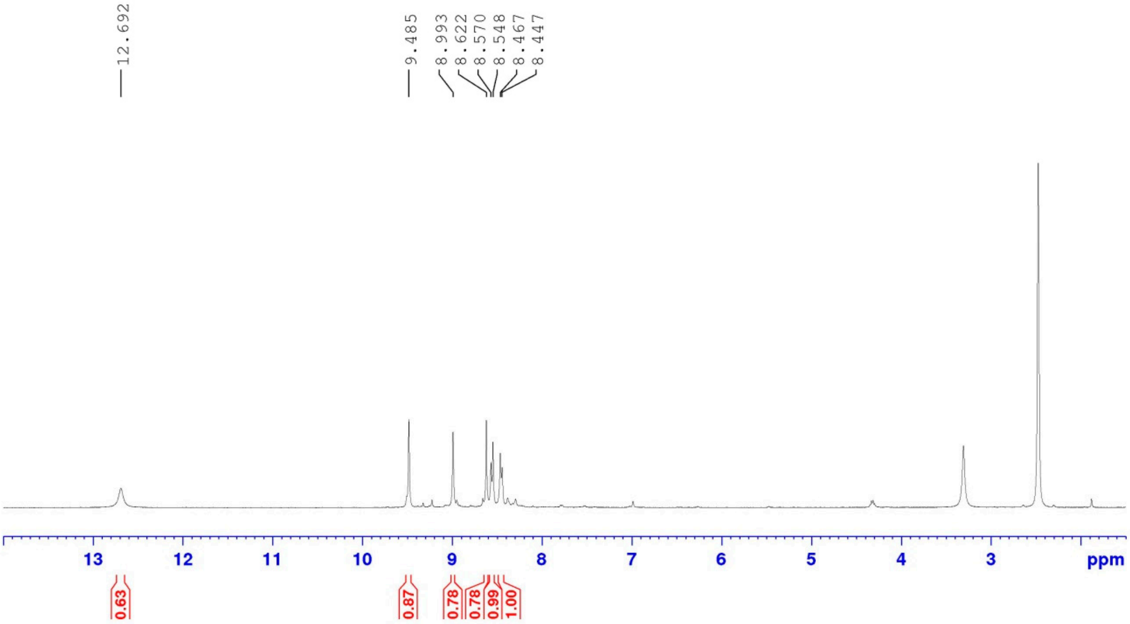

1H-NMR Compound 11a
DMSO

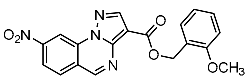

11a

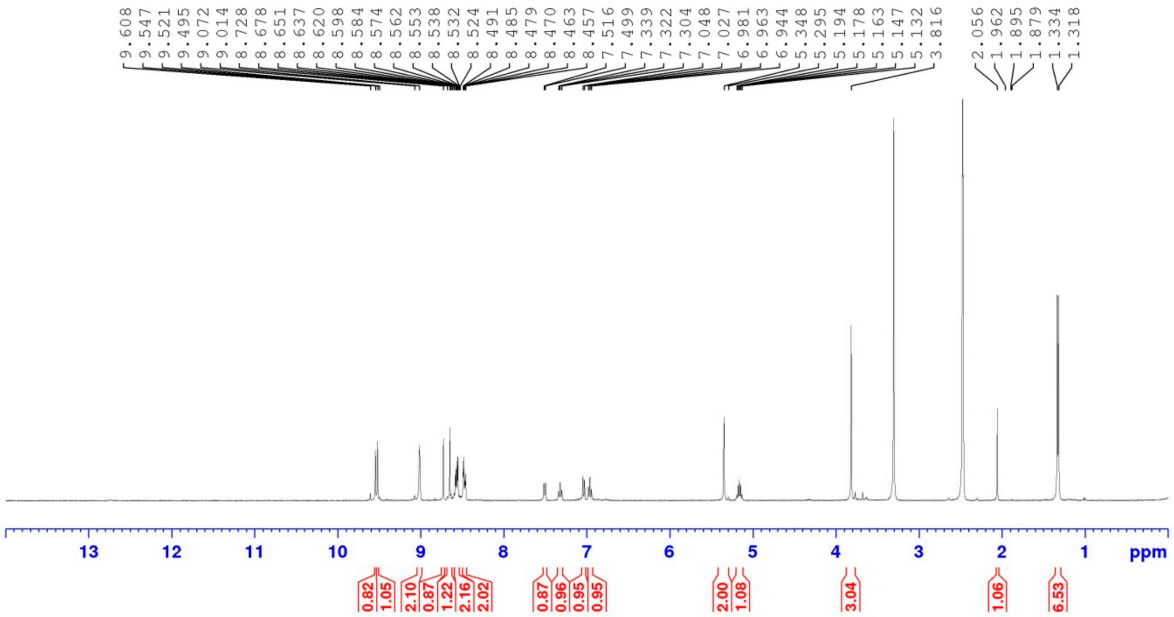

13C-NMR Compound 11b
DMSO

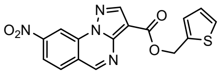

11b

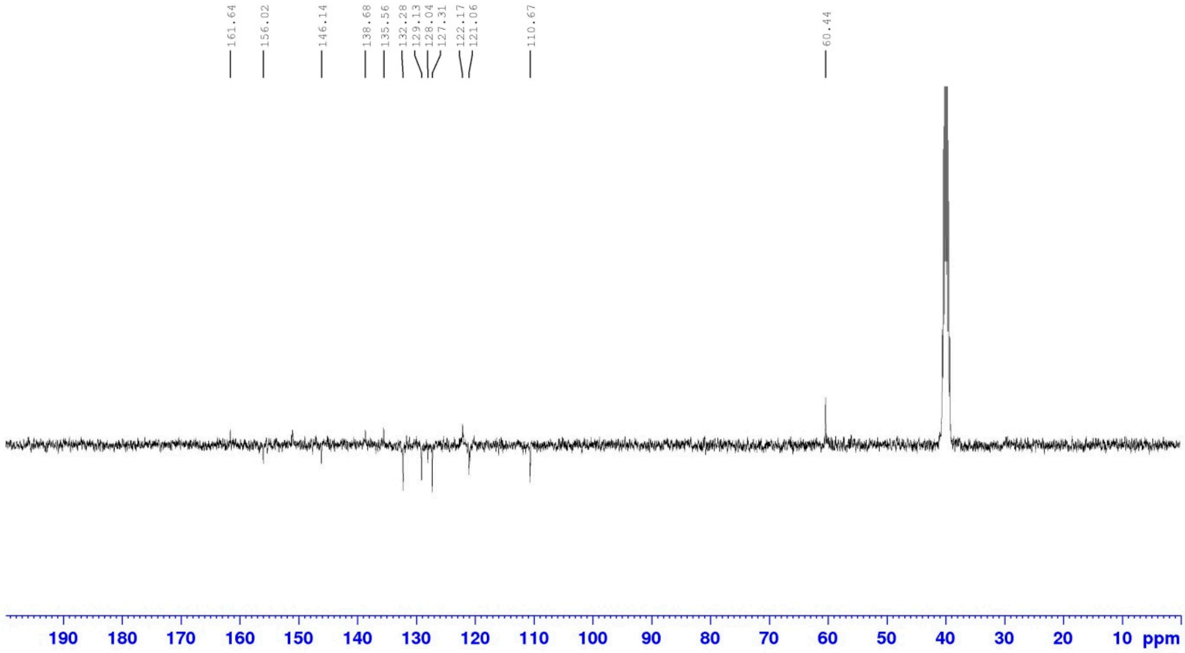

1H-NMR Compound 11b
DMSO

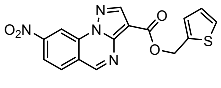

11b

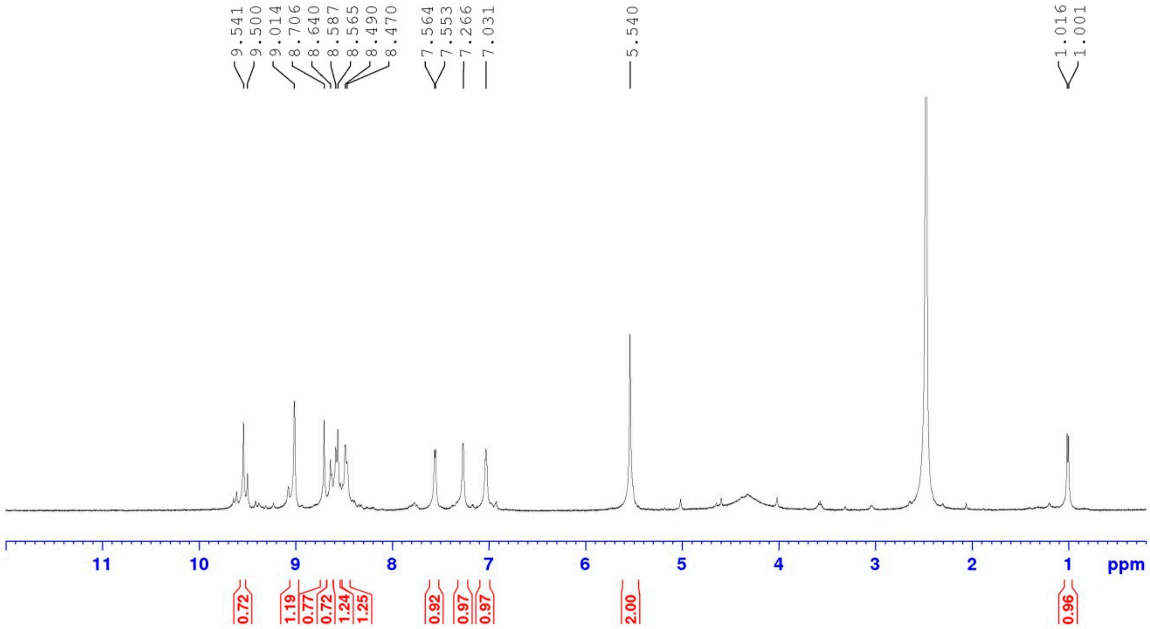

13C-NMR Compound 13
DMSO

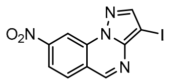

13

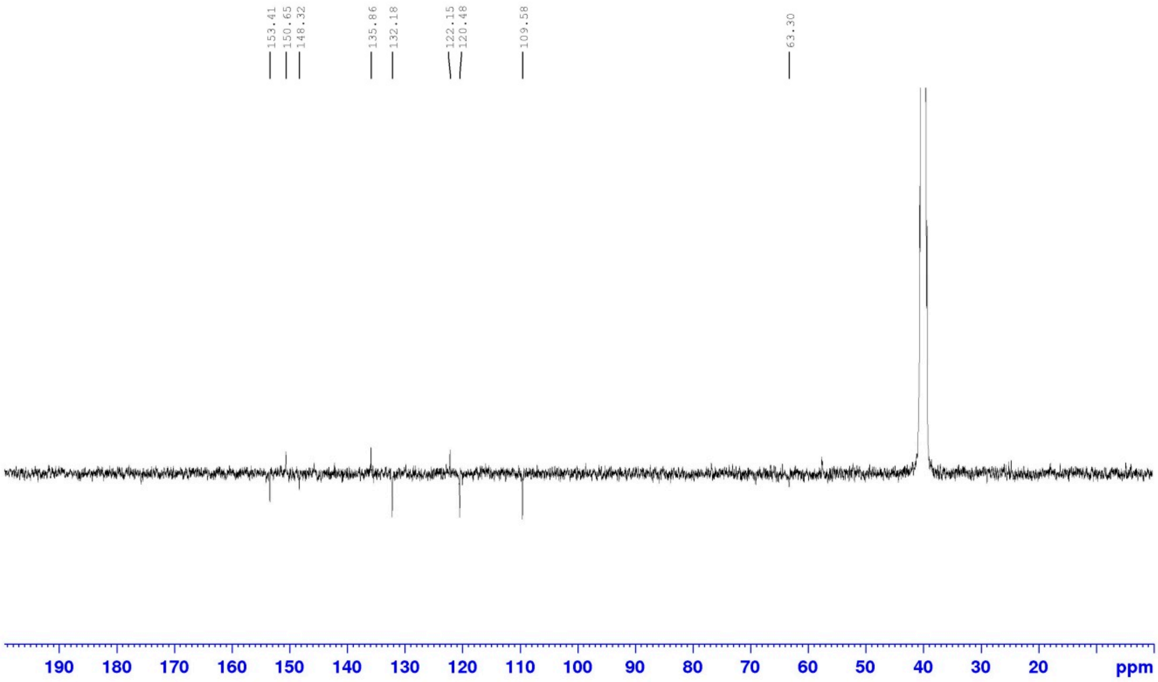

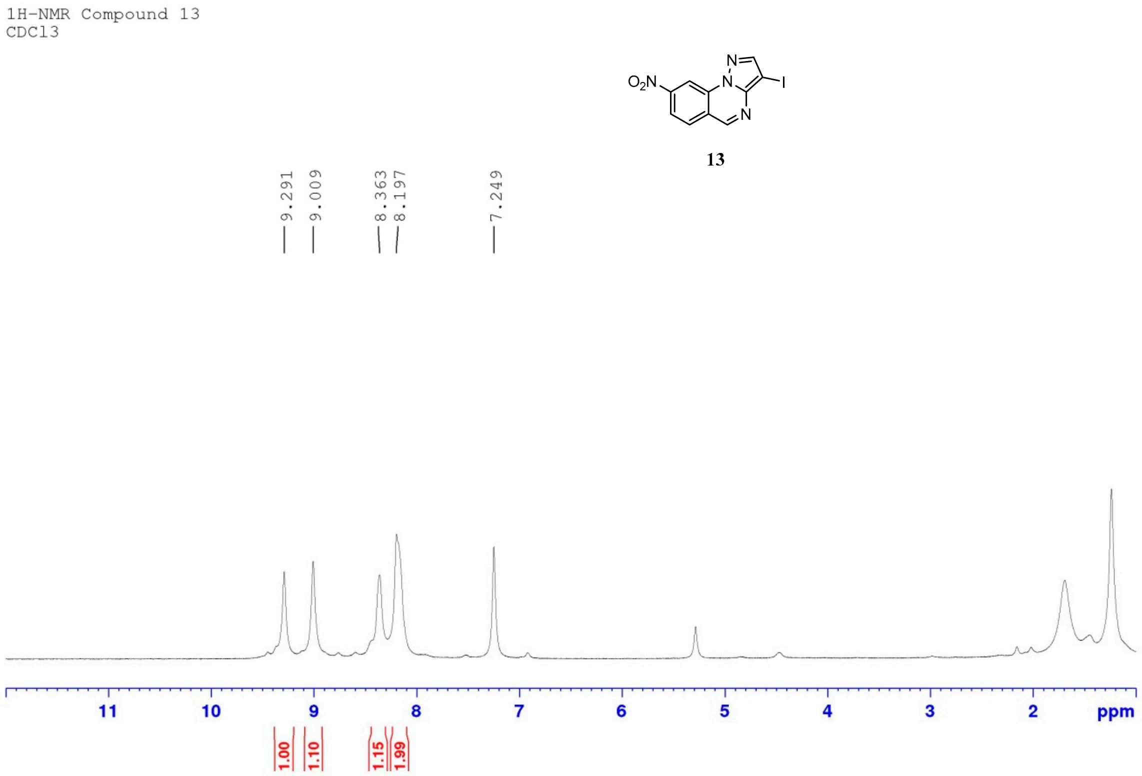
1H-NMR Compound 13
CDCl3
O_2N
N
N
I
N
13
9.291
9.009
8.363
8.197
7.249
11
10
9
8
7
6
5
4
3
2
ppm
1.00
1.10
1.15
1.99

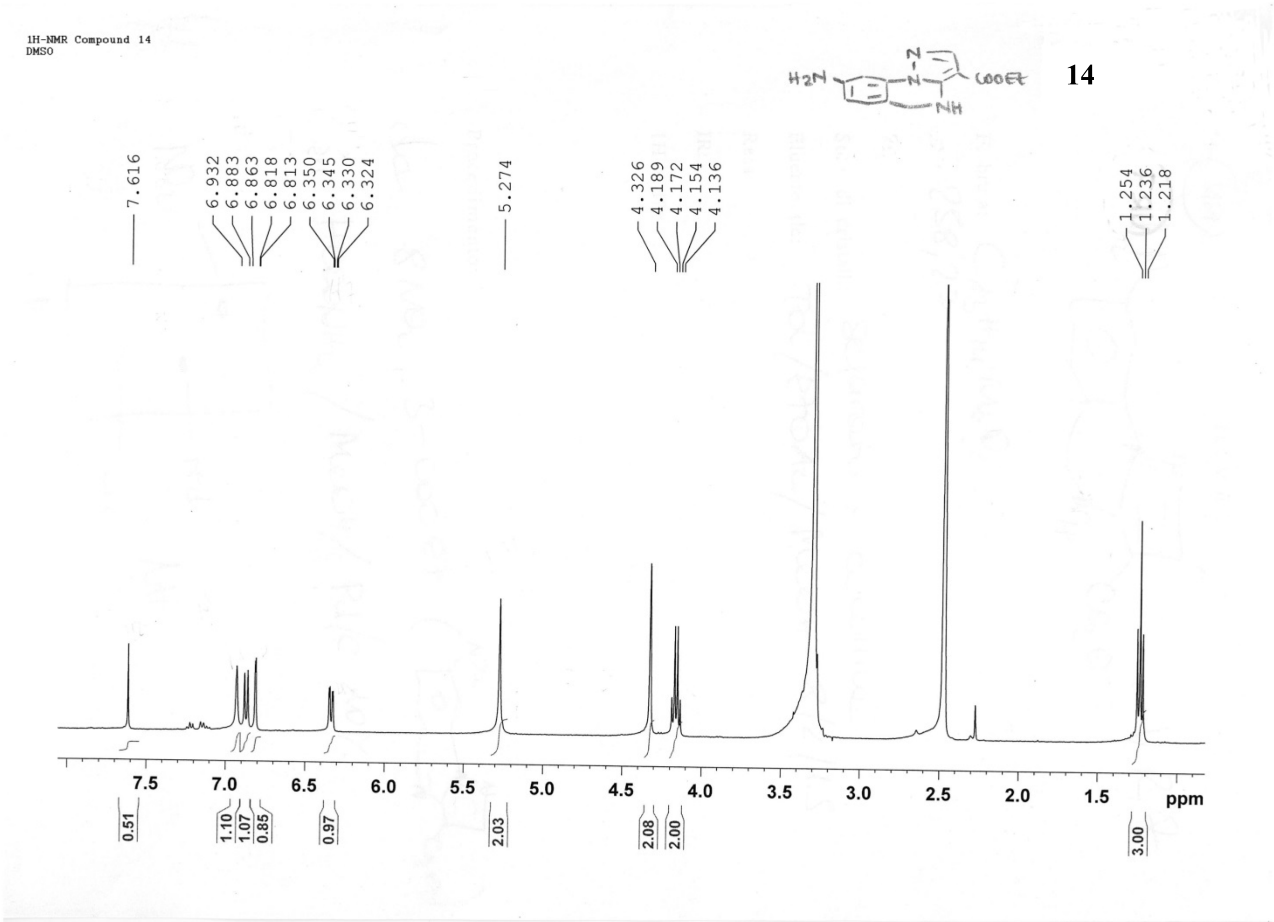
1H-NMR Compound 14
DMSO
14
7.616
6.932
6.883
6.863
6.818
6.813
6.350
6.345
6.330
6.324
5.274
4.326
4.189
4.172
4.154
4.136
1.254
1.236
1.218
7.5
7.0
6.5
6.0
5.5
5.0
4.5
4.0
3.5
3.0
2.5
2.0
1.5
ppm
0.51
1.10
1.07
0.85
0.97
2.03
2.08
2.00
3.00

13C-NMR Compound 15
DMSO

15

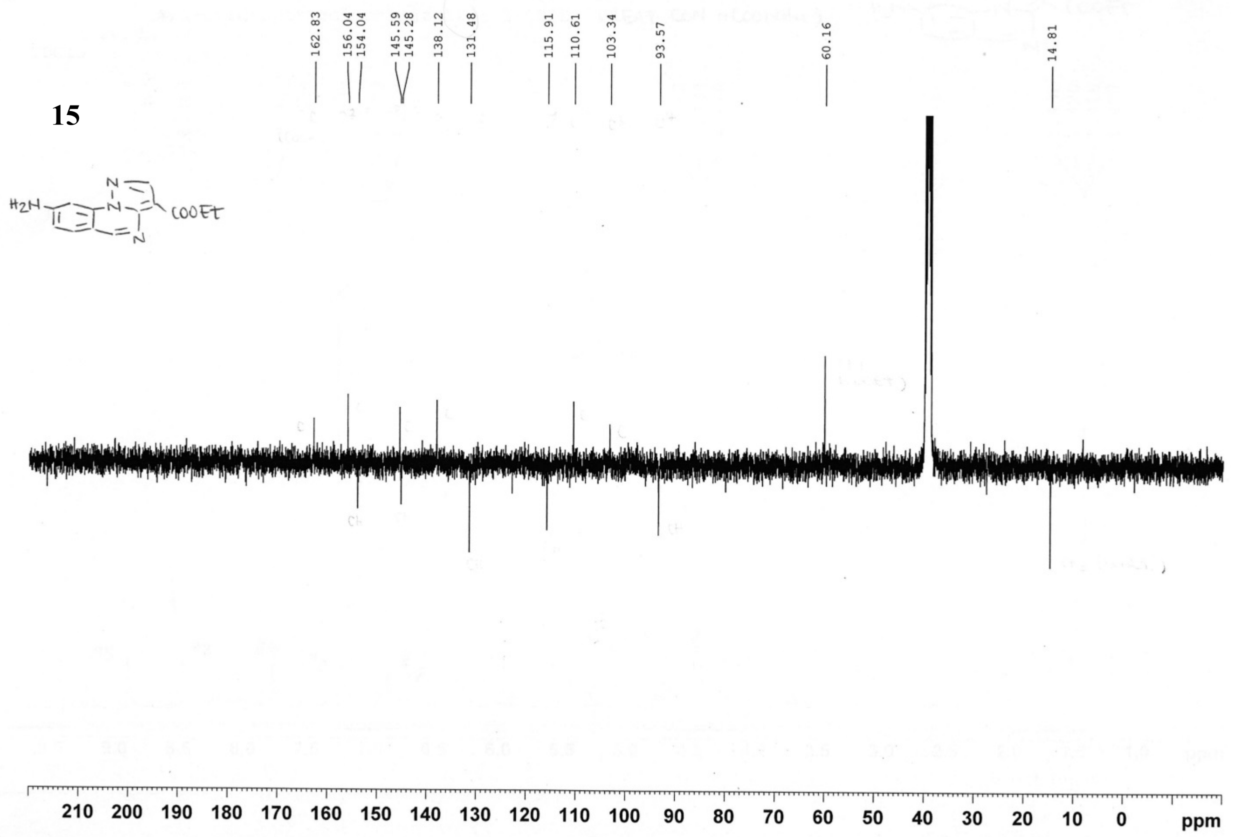

1H-NMR Compound 15
DMSO

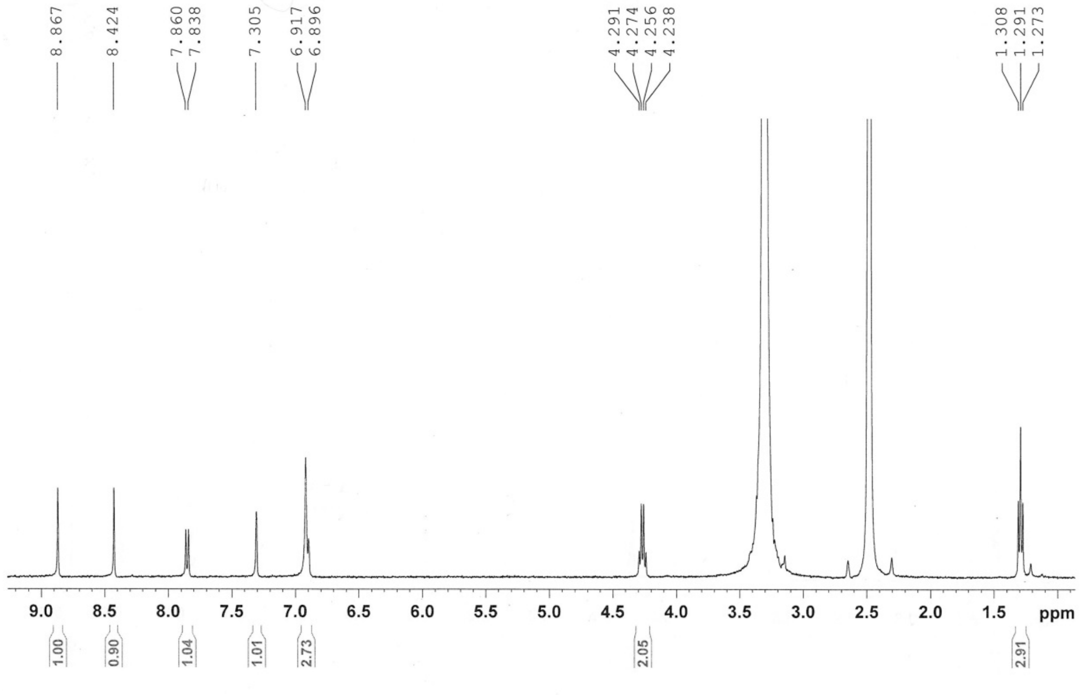

1H-NMR Compound 16
DMSO

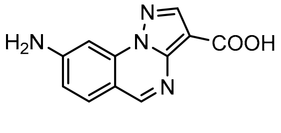

16

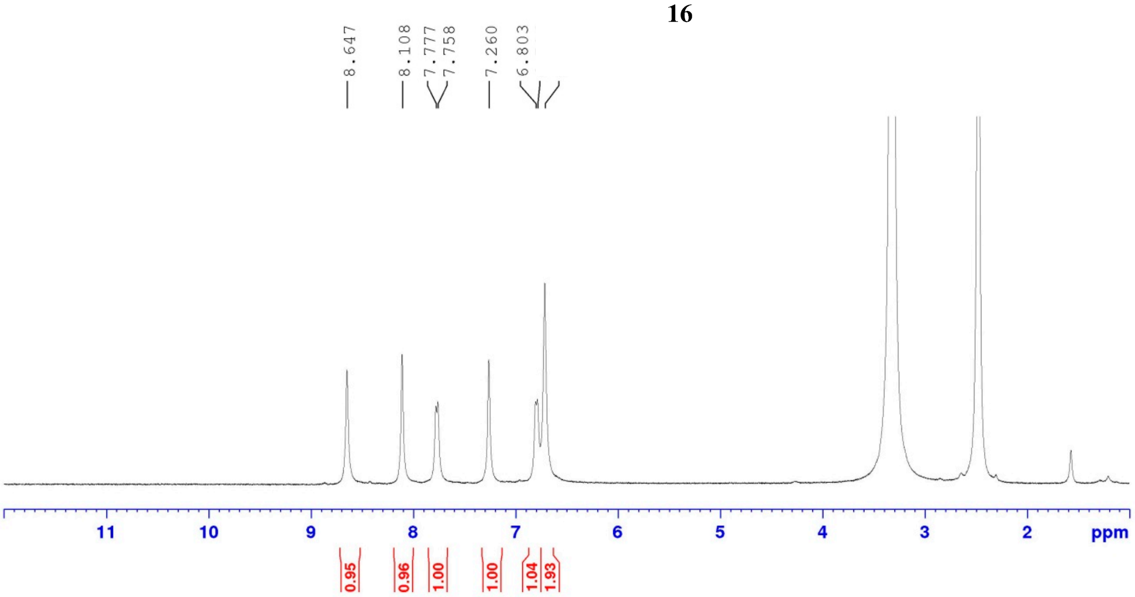

1H-NMR Compound 17a
CD3CN

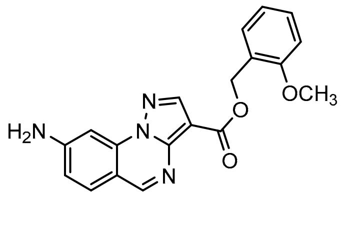

17a

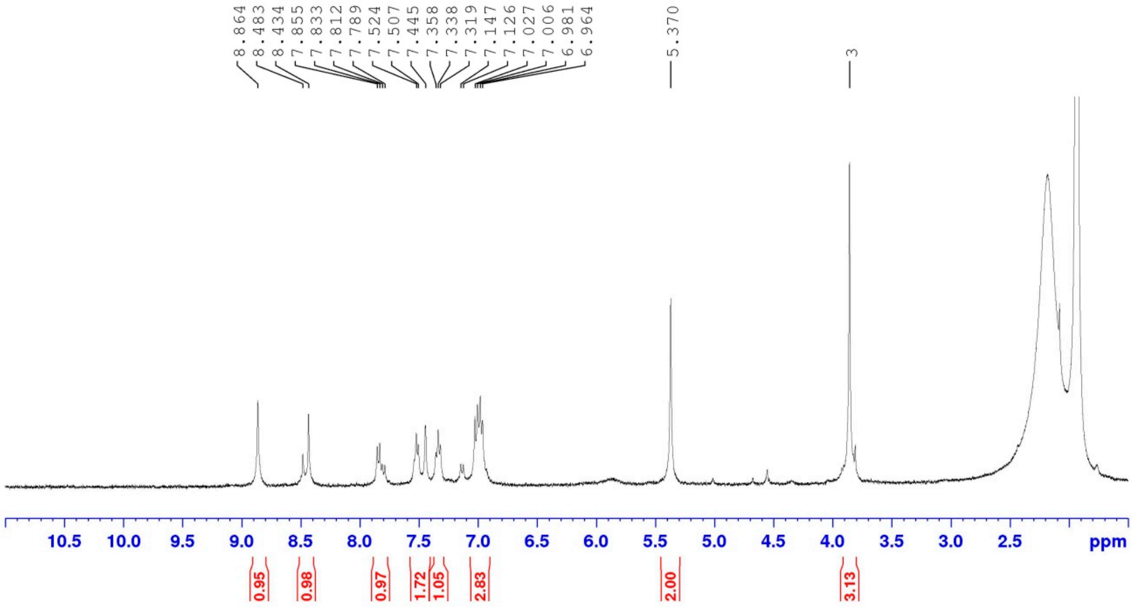

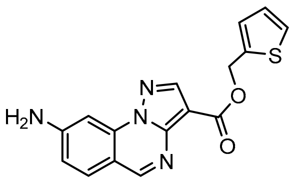

17b

13C-NMR Compound 17b
DMSO

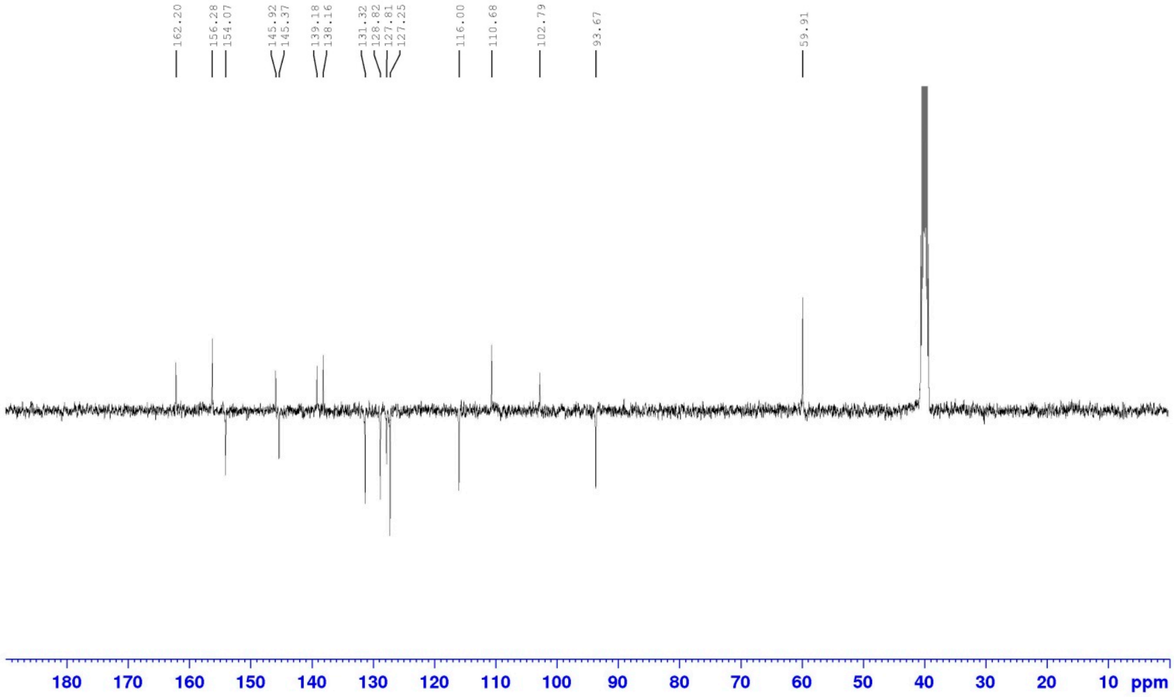

1H-NMR Compound 17b
CD3CN

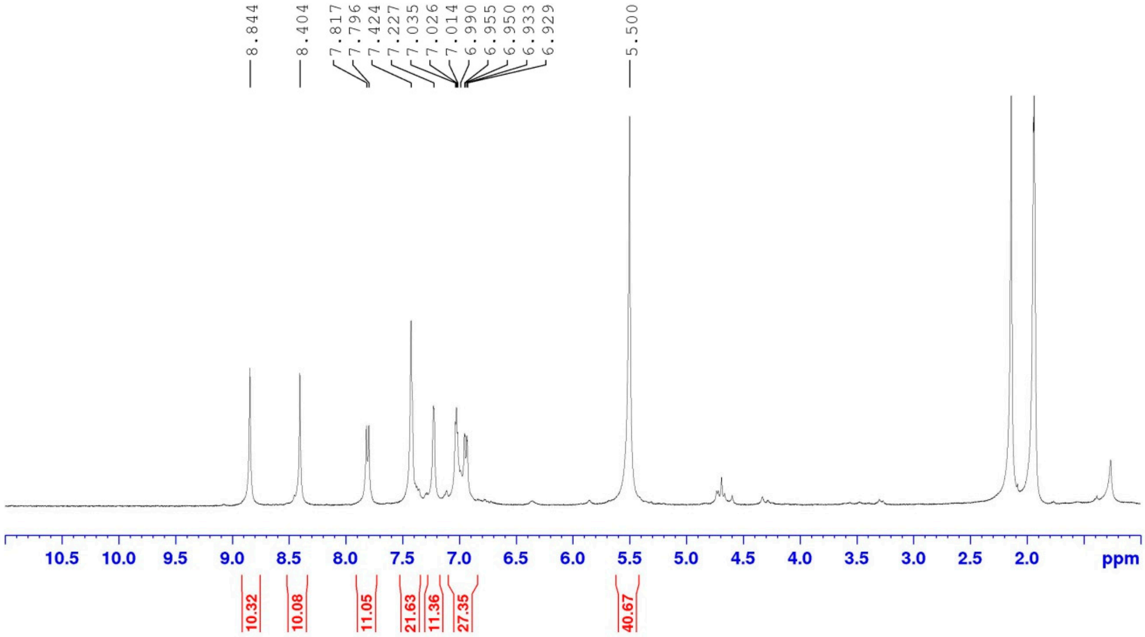

2. Electrophysiological results

A)

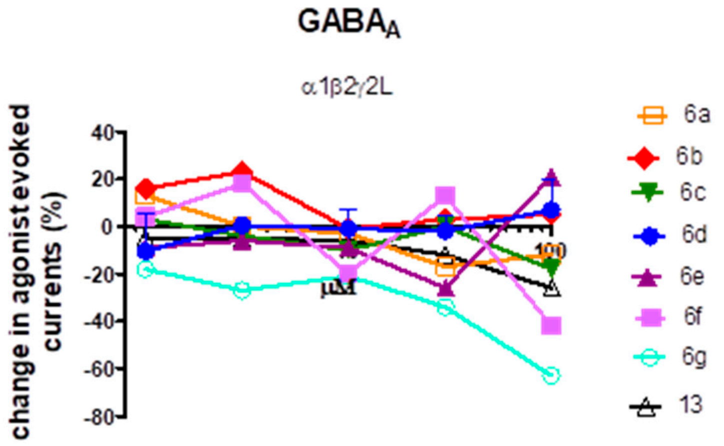

B)

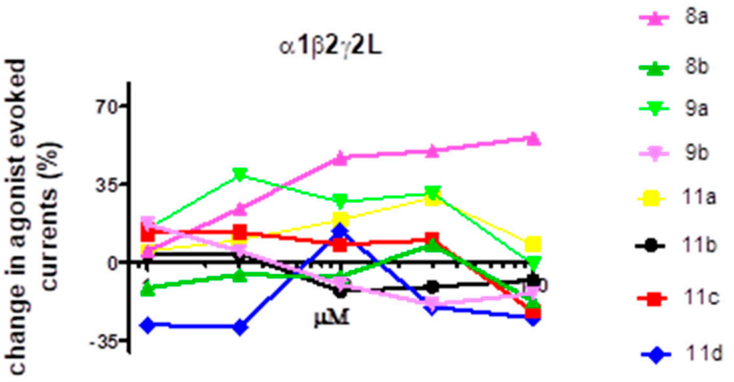

C)

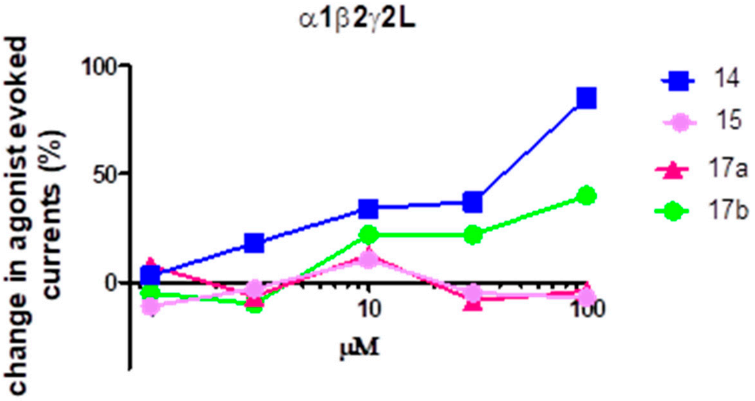

Figure S1. Concentration-response curves of compounds on GABA-induced Cl^- currents in *Xenopus laevis* oocytes expressing recombinant α1β2γ2L-GABA$_A$ receptors. Data are expressed as the percentage modulation of the response induced by GABA at EC5-10 values and are the mean ± SEM of values obtained from two to nine oocytes.

A) 3-iodo derivatives; B) 3-ester derivatives; C) 8-amino derivatives

3. Proximity Frequencies evaluation

(References number are referred to the manuscript)

The evaluation of the 'Proximity Frequencies', during the molecular dynamic simulation [13], were calculated on 19 final representative compounds (**6a-g**, **8a**, **b**, **9a**, **b**, **11a-d**, **14**, **15**, **17a**, **b**) to predict their profile on the α1β2γ2L-GABA$_A$R.

The 'Proximity Frequencies' are the frequencies with which a ligand intercepts two or more amino acid during the dynamic simulation. This frequency, expressed in percentage (%), was calculated just in the case that two or three amino acids were at a distance <0.25 nm from the ligand contemporaneously.

The value of Proximity Frequencies (PFs), used in a linear discriminant function (LDA), was able to correctly collocate 70.6 % of agonists and 72.7% of antagonists by combining a double PFs (αVal203-γThr142) with a triple PFs (αHis102-αTyr160-γTyr58). The predictive capacity was evaluated on an appropriate training set of molecules with a cross-validation 'leave one out' (LOO) procedure.

During a molecular dynamic simulation (60 ns), the agonist compounds were simultaneously close to the αVal203 and γThr142 amino acids, with a frequency of 37% compared to the frequency of 16% found by the antagonist compounds, while the antagonist compounds were simultaneously close to the αHis102, αTyr160 and γTyr58 amino acids, with a frequency of 35% against a frequency of 13% for agonist compounds. All the 3D structures of the molecules, as a training set and new final compounds, were designed [28] and placed in the binding site of the BDZs with the AUTODOCK 4.2 [18] docking program. The structure of the BDZ binding site was obtained from the recently solved GABA$_A$R structure (PDB ID 6D6T) [2]. The docking program performed on all selected compounds gave clusters of conformation(s) for each compound (rmsd 2.0).

The evaluation of trajectories in the dynamic simulation was performed on the best complex ligand-binding site conformation. The binding site is an isolated portion of the protein between the α and γ chains comprising all amino acids within a radius of 2 nm from the centre of the benzodiazepine binding site.

**Compound**	proximity frequency (%)	
	αVal203-γThr142	αHis102-αTyr160-γTyr58
**6a**	0	1
**6b**	3	39
**6c**	0	7
**6e**	37	11
**6f**	45	3
**6g**	48	1
**8a**	7	1
**8b**	1	24
**9a**	2	30
**9b**	6	2
**11a**	62	2
**11b**	3	17
**11c**	12	0
**11d**	0	1
**13**	0	1
**14**	48	44
**15**	16	2
**17a**	0	11
**17b**	4	4

Table S1. Proximity frequency values for compounds with αVal203-γThr142 and αHis102-αTyr160-γTyr58.

The proximity frequency values for compounds **8a** and **8b** are consistent with what was previously stated, in fact the agonist 8a shows a proximity frequency with αVal203-γThr142 higher than the antagonist 8b (7% and 1% respectively) (Table S1).
